# Bio‐Inspired Micro‐Fin‐Assisted Multi‐Modal Vascular Intervention

**DOI:** 10.1002/advs.202515119

**Published:** 2025-11-27

**Authors:** Xu Liu, Qiang Luo, Zhuoqun Cao, Hongde Li, Xi Chen, Hong Wang, Mao Chen, Ziyu Ren, Wenqi Hu

**Affiliations:** ^1^ Department of Mechanical and Aerospace Engineering The Hong Kong University of Science and Technology Clear Water Bay Hong Kong Kowloon P. R. China; ^2^ Cheng Kar‐Shun Robotics Institute The Hong Kong University of Science and Technology Clear Water Bay Hong Kong Kowloon P. R. China; ^3^ School of Mechanical Engineering and Automation Beihang University Beijing 100191 P. R. China; ^4^ Department of Cardiology Laboratory of Cardiac Structure and Function at Institute of Cardiovascular Diseases and Cardiac Structure and Function Research Key Laboratory of Sichuan Province West China Hospital Sichuan University No.37 Guoxue Street Chengdu 610041 P. R. China

**Keywords:** flow‐asisted, magnetic micro‐fins‐integrated tip, multi‐modal, vascular intervention

## Abstract

Interventional procedures are essential in vascular medicine, but precise navigation through tortuous vasculature remains difficult due to the limited steerability of guidewires in complex anatomical regions. Magnetic guidewires have been developed to address this limitation, yet they typically depend on accurate field control using robotic‐arm‐mounted magnets or electromagnets, which constrains their use in space‐limited intervention room. Here, a magnetic guidewire with a micro‐fin‐integrated tip for multimodal vascular intervention is introduced. The micro‐fins respond to external magnetic fields and generate additional torques through fluid drag and vessel wall contact forces, supplementing the magnetic torque and gradient forces of conventional designs. This combination enables the guidewire to pass bifurcations under lower magnetic field strength, reduced from 34 to 15 mT in the trials, and to tolerate external field misalignments up to 45 degrees. The micro‐fin structure also permits bidirectional crawling and self‐correction from buckling. In vivo testing in rabbit vascular models shows the device reduced time by ≈50% compared to a commercial guidewire, even when the magnetic field is manually applied, and allows full access to major arteries within 1 min. These findings demonstrate that micro‐fins integration provides advantages that improve the control and efficiency of magnetic guidewires in complex vascular environments.

## Graphical abstract

A magnetic guidewire with a micro‐fin–integrated tip is introduced for multimodal cardiovascular navigation. The micro‐fins exploit fluid drag to augment magnetic torque, enabling bifurcation traversal at 15 mT, tolerating 45° misalignment, bidirectional crawling, and buckle self‐correction. During in vivo tests in rabbit models, procedures are ≈50% faster than using a commercial guidewire, enabling arterial access within a minute.

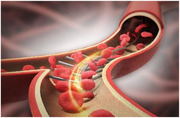

## 1 Introduction

The advancement of minimally invasive vascular procedures has generated growing demand for tools capable of safely and effectively navigating the intricate vascular system. Conventional vascular interventions rely on X‐ray fluoroscopy^[^ ^1^ ^]^ and flexible guidewires to access stenosed or occluded vessels.^[^ ^2^, ^3^ ^]^ For less complicated geometries, these procedures can also be supplemented by intravascular ultrasound (IVUS)^[^ ^4^, ^5^ ^]^ and optical coherence tomography (OCT)^[^ ^6^ ^]^ for a better view of the lesion. However, endovascular procedures can still be technically demanding, particularly in resource‐limited environments,^[^ ^7^ ^]^ among less‐experienced operators,^[^ ^8^ ^]^ and in complex vasculature.^[^ ^9^ ^]^ Under these conditions, inadvertent guidewire buckling, misplacement, or perforation can lead to severe complications, including vascular injury, bleeding, infection, and cardiac perforation.^[^ ^10^, ^11^ ^]^

To address these limitations, efforts have been made to endow the guidewire with active steerability at its tip. Techniques include shape memory alloys,^[^ ^12^, ^13^ ^]^ tendon‐driven,^[^ ^14^, ^15^, ^16^ ^]^ hydraulic^[^ ^17^, ^18^, ^19^, ^20^ ^]^ and magnetic‐controlled^[^ ^21^, ^22^, ^23^, ^24^ ^]^ guidewire tips. Among these, magnetically controlled guidewires offer notable advantages as they allow wireless control,^[^ ^25^ ^]^ rapid, precise modulation,^[^ ^26^ ^]^ and device miniaturization.^[^ ^27^ ^]^ To develop magnetic guidewires, further improvements in passability have been achieved by applying hydrophobic hydrogel coating^[^ ^28^, ^29^ ^]^ and constructing microstructures such as a dexterous helical pattern.^[^ ^30^, ^31^ ^]^ Fluidic field^[^ ^22^ ^]^ and actively actuated cilia^[^ ^23^ ^]^ are also utilized for navigation. However, steering these magnetic guidewires often requires precise control of the external magnetic field, generated by either electromagnets or permanent magnets, to ensure spatial uniformity,^[^ ^24^, ^32^ ^]^ sufficient field strength,^[^ ^33^, ^34^, ^35^ ^]^ and precise alignment.^[^ ^21^, ^29^, ^36^, ^37^, ^38^ ^]^ These requirements increase the cost associated with sensing and control. Moreover, the need to integrate large equipment such as coil arrays and robotic arms into already crowded interventional environments presents additional practical challenges, limiting the clinical translation of magnetic guidewires.

To reduce these constraints while preserving the benefits of magnetic actuation, we propose a magnetic guidewire design incorporating bio‐inspired micro‐fins that combine passive flow‐guided deformation with active magnetic control. The tilting angles of the micro‐fins can be modulated by external magnetic fields. This design adds new torque sources to the guidewire: the micro‐fins generate extra torque from blood flow drag and contact forces against the vessel wall, beyond the magnetic torque and gradient forces used in conventional designs. With the assistance of these additional torques, the guidewire can navigate vascular bifurcations under reduced magnetic field strength.

In medical phantom tests, a conventional magnetic guidewire we fabricated required 34 mT to access a branch with lower flow velocity, whereas the micro‐fin‐integrated version reduced this requirement to 15 mT and tolerated magnetic field misalignments of up to 45 degrees. The micro‐fin structure also enabled bidirectional crawling and allowed the guidewire to self‐correct buckling when entanglement occurred.

The performance of the micro‐fin‐integrated tip was further validated in vivo using rabbit vascular models. In interventional trials, it reduced traversal time by ≈50% compared to a commercial guidewire, even when the external magnetic field was manually controlled. The main arteries of the rabbit were successfully traversed within 1 min. These findings indicate that the incorporation of micro‐fins can enhance the adaptability of magnetic guidewires to complex vascular geometries while reducing external control requirements, offering a pathway toward more streamlined and effective interventional procedures.

## 2 Results

### 2.1 Design

**Figure 1:**
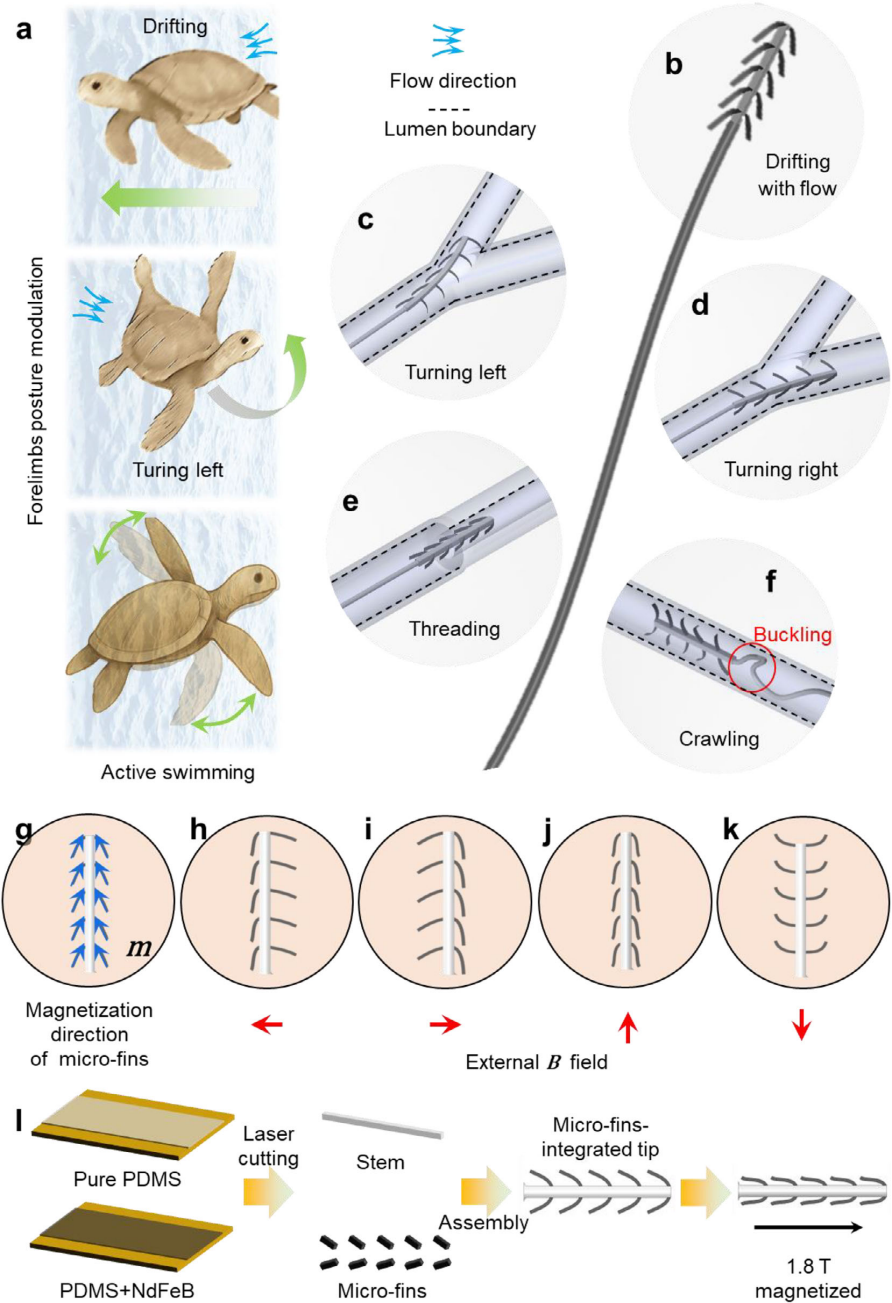
Design methodology of magnetically controlled bio‐inspired micro‐fins for flow‐assisted multi‐modal vascular intervention. Natural inspiration of body structure reconfiguration from a) sea turtles adjusting forelimb positions for drifting, turning, and active swimming in a fluidic environment. Magnetically controlled bio‐inspired micro‐fins achieving multiple functions: b) Drifting along flow direction, c,d) Turning left and right, e) Threading into tapered vessels with flow, and f) Crawling to unbuckle the soft guidewire. g) Magnetization direction of micro‐fins. Structural reconfiguration under different *B* field corresponding to function (c–f): h,i) single micro‐fins closed, j) both micro‐fins closed, and k) both micro‐fins open. l) Fabrication process of magnetically controlled bio‐inspired micro‐fins.

Many aquatic animals achieve functional versatility by dynamically reconfiguring their body structures to interact with surrounding fluid environments. For instance, sea turtles use their forelimbs not only for propulsion but also to adjust body pitch and limb extension, thereby modulating lift and enabling directional control while drifting with ocean currents during migration^[^ ^39^ ^]^ (**Figure** **1**a). Inspired by this biological strategy and aiming to improve the steerability of guidewire tips, we developed a magnetically controlled, bio‐inspired micro‐fin design (Figure 1b). The structure consists of a pair of magnetic micro‐fins laterally mounted on a soft, nonmagnetic central stem. These fins can passively respond to local flow dynamics while also being actively reconfigured via the external magnetic field.

With such a design, the micro‐fins‐integrated tip enables four functional modes: (1**) Passive drift** (Figure 1b). In the absence of a magnetic field, the micro‐fins are dragged by the flow, allowing the entire tip to drift along the fluid streamlines; **(2) Robust turning** (Figure 1c,d). When a magnetic field opens the micro‐fins on one side, the tip can turn. This turning is facilitated by magnetic torque, fluid drag‐induced torque, and wall‐contact reaction torque generated by the micro‐fins pressing against the vessel wall. As a result, turning is more efficient compared to conventional magnetic guidewires; **(3) Threading through narrow openings** (Figure 1e): When approaching tight regions, the micro‐fins on both sides can be closed to allow the tip to thread through restricted passages; **(4) Self‐unbuckling** (Figure 1f). In the event of buckling or entanglement, a rotating magnetic field can activate a crawling motion at the tip, enabling it to self‐unbuckle and recover its orientation. The magnetization directions of the micro‐fins are shown in Figure 1g, and the configurations corresponding to each functional mode under different magnetic fields are shown in Figure 1h–k. The tip is fabricated through a combination of laser cutting and manual assembly (Figure 1l; Note S1, Supporting Information).

**Figure 2:**
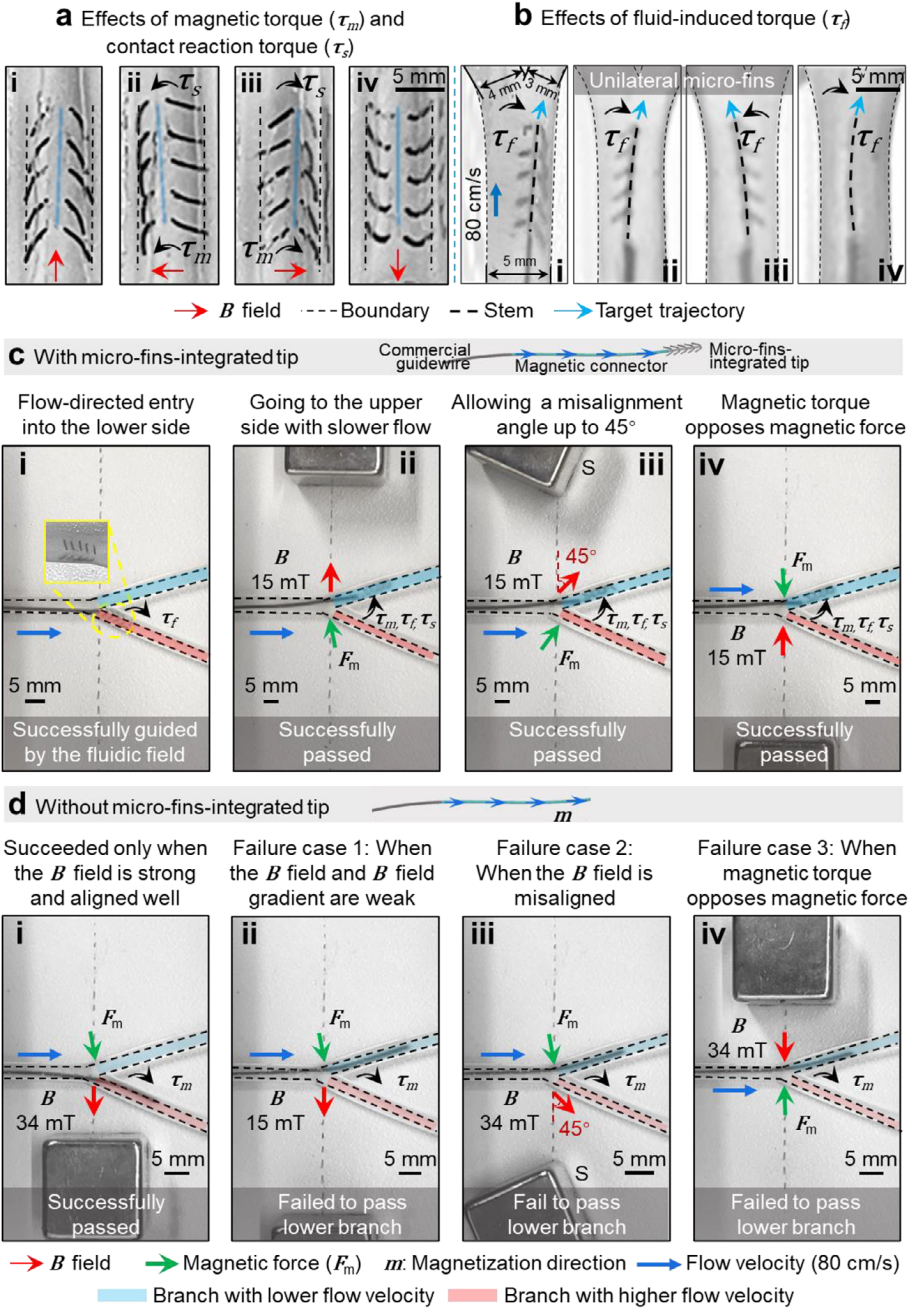
Comparisons of the requirements on ***B*** field between conventional magnetic guidewires and the magnetic guidewires with proposed micro‐fins. a) Effects of magnetic torque and contact reaction torque. Four shape morphing modes of the micro‐fin‐integrated tip in a phantom under a static ***B*** field. The magnetic torque (***τ*** *_m_*) and contact reaction torque from the boundary (***τ*** *_s_*) are clearly marked. b) Effects of fluid‐induced torque. Micro‐fin‐integrated tip (i–iii) and a straight guidewire (iv) were placed in a Y‐shaped phantom. Torque attributed to fluidic field (***τ*** *_f_*) is clearly marked. c) Guidewire with micro‐fin‐integrated tip exhibits low requirements on magnetic field conditions. i) Proposed guidewire enters the lower branch with higher flow velocity solely with fluidic guidance. ii) The proposed guidewire can enter the lower branch with lower flow velocity under a lower ***B*** field strength (15 mT). iii) The proposed guidewire can tolerate a misalignment angle up to 45°. iv) The proposed guidewire can still go to the upper branch when the magnetic force acts in opposition to the magnetic torque. d) Guidewire without the micro‐fin‐integrated tip requires a more stringent external ***B*** field. i) The guidewire without micro‐fins can only go to the lower branch when the magnetic field is strong (34 mT) and well‐aligned. If no ***B*** field is applied, the guidewire goes to the upper branch. ii) Failure case 1: when the ***B*** field is weak (15 mT). iii) Failure case 2: when the ***B*** field is misaligned. iv) Failure case 3: when the magnetic torque opposes the magnetic force. Phantom 4 in Table S8 (Supporting Information) is used for (b–d) (*Φ_Intlet_* = 2.4 mm, *Φ_Outlet1_* = 2 mm, *Φ_Outlet2_* = 1.5 mm, these diameters are selected based on the DSA images of the rabbit's femoral arteries). According to the ultrasound evaluation of the rabbit femoral artery, the flow rate was distributed as 42.8% for the upper branch and 57.2% for the lower branch. The flow velocities were controlled to be ***v*** *_Inlet_* = 80 cm s^−1^, ***v*** *_Outlet1_* = 51 cm s^−1^, ***v*** *_Outlet2_* = 118 cm s^−1^. To ensure the passability, the stiffness‐gradient magnetic connector is used in both (c,d). The guidewire in (c) is composed of 3 parts: commercial guidewire, stiffness‐gradient magnetic connector, and the micro‐fin‐integrated tip, while that in (d) contains only commercial guidewire and stiffness‐gradient magnetic connector.

**Figure** **2**a presents experimental results demonstrating four distinct structural modes of the proposed tip within a 2.4 mm‐diameter tube in static flow. When the micro‐fins open on one side and close on the other (Figure 2a‐ii,iii), the opened fins press against the vessel wall, generating a contact reaction force and its associated torque (τ_s_). This torque, together with the magnetic torque (τ_m_), induces directional deflection of the tip stem. The influence of boundary conditions, particularly the tube diameter, on micro‐fin deflection is shown in Figure S1 (Supporting Information). In extremely confined environments, deformation of the central connector may be limited. Nevertheless, the enhanced steerability described above remains effective.

Figure 2b shows experimental results of the micro‐fin structural modes under fluidic flow. The tip was tested in a Y‐shaped phantom under controlled conditions (Figure 2b; *Φ_Intlet_* = 2.4 mm, *Φ_Outlet1_* = 2 mm, *Φ_Outlet2_* = 1.5 mm; ***v*** *_Inlet_* = 80 cm s^−1^, ***v*** *_Outlet1_* = 51 cm s^−1^, ***v*** *_Outlet2_* = 118 cm s^−1^). In the undeformed configuration (Figure 2b‐i), the fluidic drag force acting on the micro‐fin‐integrated tip causes it to deflect toward the right branch, where the flow is faster. Next, we investigated the configuration in which the micro‐fins on one side are open while those on the opposite side are closed (Figure 2b‐ii,iii). To isolate the effect of fluidic drag, no magnetic field was applied during this experiment. Instead, one set of fins was fixed to the stem using adhesive, while the other side remained free to deform. This setup allowed us to focus exclusively on the influence of flow‐induced forces. Under this configuration, the tip deflects toward the side opposite the open fins, driven by an asymmetric fluidic drag torque (***τ_f_***). As a control, Figure 2b‐iv presents a conventional guidewire tip placed at the same bifurcation. While it also deflects toward the branch with stronger flow, the deflection magnitude is smaller than that observed with the micro‐fin design (Figure 2b‐i,ii). Similar deflection behavior is also reproduced in fluid–structure interaction (FSI) simulations, as detailed in Notes S2 and S3 (Supporting Information).

The above experiments examined each contributing torque in isolation, but the system is designed for these torques to act cooperatively. For example, when an external magnetic field is applied leftward, the micro‐fins on the left side close while those on the right side open (Figure 2a‐ii). In this configuration, the magnetic torque (***τ*** *_m_*) rotates the stem to the left, while the right‐side micro‐fins generate a wall‐contact reaction torque (***τ*** *_s_*) that also pushes the tip leftward, as shown in Figure 2a‐ii. Simultaneously, the asymmetric fluidic drag (***τ*** *_f_*) further contributes to leftward deflection (Figure 2b‐iii). These combined effects result in an amplified steering response. Through such a cooperative steering mechanism, the tip with micro‐fins can reach target locations with a less demanding external ***B*** field. To illustrate this performance improvement, we conducted a comparative study on magnetic guidewires with and without the proposed micro‐fins. A similar Y‐shaped bifurcation phantom as that in Figure 2a,b was used, as shown in Figure 2c,d and Movie S1 (Supporting Information).

Figure 2c‐i demonstrates that the magnetic guidewire with a micro‐fin‐integrated tip can naturally drift into the lower branch, where the flow velocity is higher, purely under fluidic influence, without external control. In contrast, Figure 2c‐ii shows that the guidewire can also be steered into the upper branch, which has a slower flow velocity, by placing a permanent magnet 23 mm away, generating a magnetic field strength of 15 mT at the bifurcation junction. Since a permanent magnet (18 mm × 18 mm × 18 mm) is used, an additional torque is generated by the magnetic field gradient (***F_m_***), which assists in pulling the guidewire upward in this case. The corresponding magnetic field distribution is shown in Figure S8 (Supporting Information). As a control, we also reversed the flow distribution, assigning a higher velocity to the upper branch and a lower velocity to the narrower lower branch (Figure S9, Supporting Information). Even under this condition, the guidewire successfully entered the low‐flow lower branch when the magnet was placed at the same distance, confirming the guidewire's ability to overcome flow bias through the proposed steering mechanism.

Figure 2c‐iii further demonstrates the robustness of the steering mechanism. The guidewire can be effectively directed even when the magnet is misaligned by up to 45°, positioned 15 mm away (*B*  =  15 mT), or placed on the opposite side of the bifurcation at a distance of 23 mm (*B*  =  15 mT), where the torque generated by the magnetic gradient force (***F_m_***) opposes the other steering torques (Figure 2c‐iv). This tolerance to misalignment is particularly advantageous in clinical settings, where the permanent magnet must remain outside the patient's body and cannot always be positioned on the preferred side of the blood vessel.

The misalignment tolerance was also verified under different geometry and ***B*** field strength, as shown in Figure S10 (Supporting Information). The flow velocity of inlets is set to be 80 cm s^−1^. The micro‐fins went into the branch with lower flow velocity under a misalignment tolerance of 45 degree regardless of the coherence of magnetic force in different vascular geometries, as shown in Figure S10a (Supporting Information). Figure S10b (Supporting Information) showed the misalignment tolerance under different ***B*** field strength. Here, we fixed the geometry as phantom 4 mentioned in Table S8 (Supporting Information), and the ***B*** field strength varied from 10 to 56 mT. It was presented that the lower ***B*** field strength of 10 mT is not adequate to guide the micro‐fins from branch with higher flow velocity to that with lower one. While when ***B*** field strength increased from 15 to 56 mT, all the cases succeeded, which indicated the threshold of the trigger ***B*** field to be 15 mT. Considering that this test is to validate the in vivo scenario of the femoral artery of the rabbit, and the flow velocity difference of the two branches is to be 60 cm s^−1^ (Figures S3–S5, Supporting Information). The required ***B*** field strength will decrease with the difference of the flow velocity declines, which is below 15 mT mentioned above, convincing the consistence of the misalignment tolerance across different vascular geometries, flow velocities, and magnetic field strengths.

To figure out the flow velocity threshold for activating the functionality the micro‐fins under fluidic field, we commit the following comparisons as shown in Figure S11 (Supporting Information). The flow velocity differences of the two branches varied from 0 to 60 cm s^−1^ while remaining that of inlet to be 80 cm s^−1^. When the flow velocity difference is below 20 cm s^−1^, the micro‐fins failed to enter the branch with higher flow velocity, illuminating that low flow velocity cannot provide sufficient fluidic torque for the micro‐fins to navigate. While the micro‐fins succeeded when the flow velocity differences increased from 20 to 60 cm s^−1^, indicating the flow velocity threshold to be 20 cm s^−1^.

Further, we committed experiments for comparisons between torques generated from fluidic field and magnetic field as shown in Figure S12 (Supporting Information). The fluidic torque rose from 0.18 to 0.61 µN·m with difference of flow velocity increased (from 0 to 60 cm s^−1^, the flow velocity difference of 60 cm s^−1^ is the condition of femoral artery in the rabbit model), indicating that the increasing uneven distributed fluidic drag would result in larger torque on the micro‐fins for deflection toward bifurcation entry (Figure S12a, Supporting Information). The magnetic torque was measured using a rheometer (Anton Paar MCR302) as shown in Figure S12b (Supporting Information). The magnetic field strength was varied from 7 to 115 mT when the distance between magnet and micro‐fins decreased from 40 mm to 5 mm. Further, the corresponding magnetic torque rose from 0.33 to 2.21 µN·m. What's more, the magnetic torque reached 0.85 µN·m when the external ***B*** field strength was 15 mT, which was applied during the calibration section in Figure 2c,d. This higher magnetic torque over fluidic torque ensures that the micro‐fins could be navigated into the branch with lower flow velocity by the external ***B*** field, validating the maneuverability and passability of the proposed micro‐fins.

Note that the micro‐fins are the only variable between the experiments described above. All other components of the magnetic guidewires remain identical, as shown in the upper panels of Figure 2c,d. In summary, the addition of micro‐fins significantly enhances the effective torque available for steering, while reducing the requirements for the external magnetic field in terms of strength, spatial uniformity, and allowable misalignment angle.^[^ ^24^, ^26^, ^28^, ^29^, ^30^, ^31^, ^32^, ^33^, ^34^, ^35^, ^40^, ^41^, ^42^, ^43^, ^44^, ^45^, ^46^ ^]^ Detailed comparisons with previous work are provided in Table S2 (Supporting Information).

### 2.2 Characterization of Micro‐Fin‐Integrated Tip and Stiffness‐Gradient Magnetic Connector

**Figure 3:**
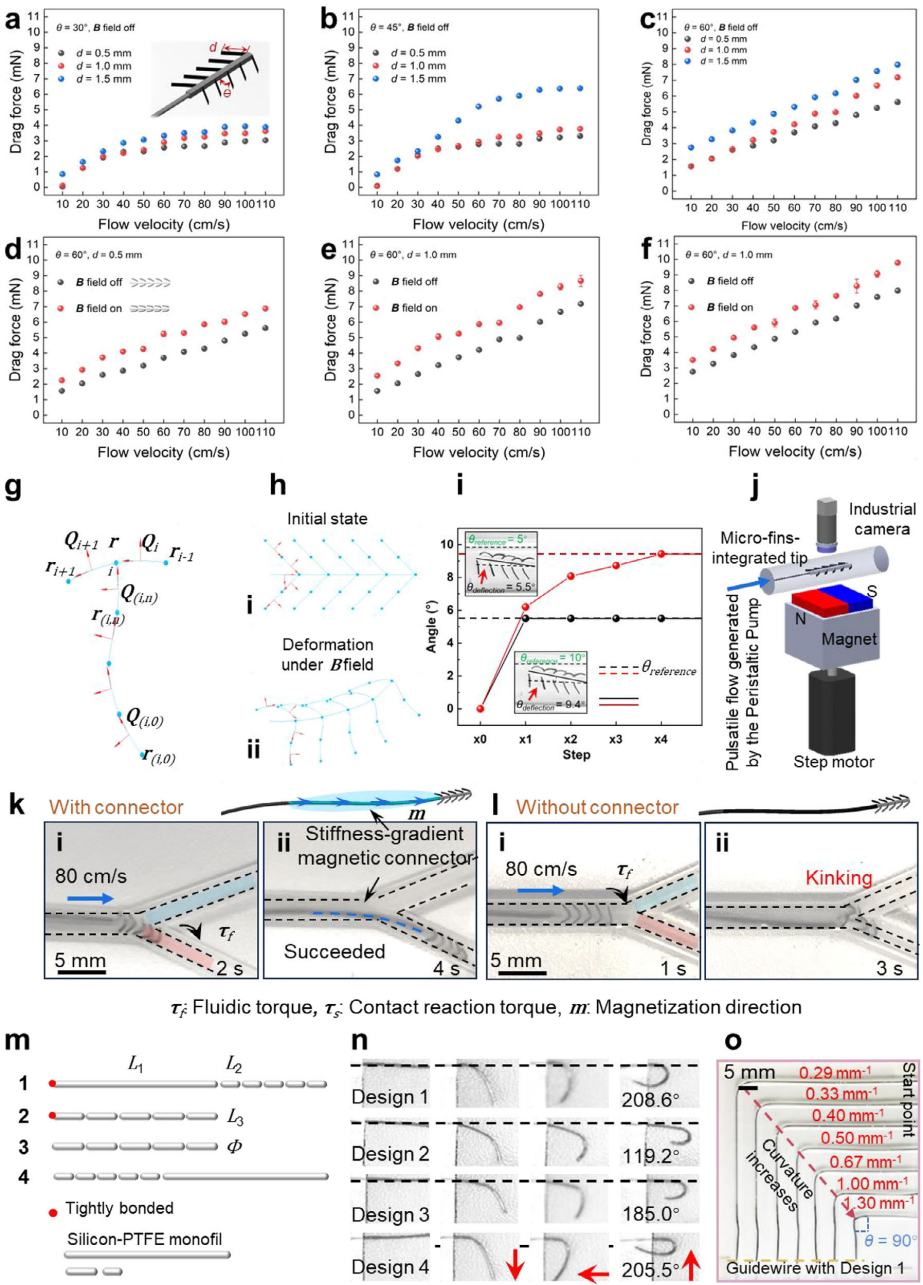
Characterization of micro‐fin‐integrated tip and stiffness‐gradient magnetic connector. Characterization of the drag force under different flow velocities for different design parameters: a) *θ* = 30°, *d* varies from 0.5 to 1.5 mm, b) *θ* = 45°, *d* varies from 0.5 to 1.5 mm, c) *θ* = 60°, *d* varies from 0.5 to 1.5 mm. Characterization of the drag force with and without the ***B*** field under different flow velocities d) *θ* = 60°, *d* = 0.5 mm, e) *θ* = 60°, *d* = 1.0 mm, f) *θ* = 60°, *d* = 1.5 mm. Data are presented as mean ± standard deviation with *n* = 5. The *θ* in (a–f) refers to the original fabrication angle rather than the dynamic angle under ***B*** field (*θ_d_*). g,h) Dynamic modeling of the micro‐fin‐integrated tip. The tip could be discretized into N vertices ***r*** *_i,i = 0,…,N_* (blue segments) and N‐1 material frames ***Q*** *_i,i = 0,…,n‐1_* (arrows in red) from the base to the tip. i) Dynamic control of the deflection angle of the micro‐fin‐integrated tip under an external magnet. The deflection angle gradually converged to the reference angle after 4 control steps. j) Experimental setup for dynamic control of the deflection angle of the magnetic micro‐fins under an external magnet. The stiffness‐gradient magnetic connector is essential for connecting the micro‐fin‐integrated tip and commercial guidewires. Comparison of guidewires going through Y‐shaped branches k) with and l) without a stiffness‐gradient magnetic connector. (Phantom 4 in Table S8 (Supporting Information) is used; *Φ_Intlet_* = 2.4 mm, *Φ_Outlet1_* = 2 mm, *Φ_Outlet2_* = 1.5 mm; ***v*** *_Inlet_* = 80 cm s^−1^, ***v*** *_Outlet1_* = 51 cm s^−1^, ***v*** *_Outlet2_* = 118 cm s^−1^). The guidewire without a connector kinks at the bifurcation, while the one with a connector can successfully pass entry point under fluidic guidance. m) Four different designs of a stiffness‐gradient magnetic connector. The parameters are *L_1_* = 10 mm, *L_2_* = 1 mm, *L_3_* = 2 mm, and *Φ* = 0.2 mm. n) Maximum deflection angle of the four designs. o) The design 1 can pass bending tubes in 90° with different curvatures (*κ* = 0.33 to 1.3 mm^−1^) (Phantom 6 in Table S8, Supporting Information).

The key design parameters of the micro‐fin‐integrated tip are summarized in Table S3 (Supporting Information), with the fin diameter (*d*) and initial angle (*θ*) identified as the two primary variables influencing device performance (Note S4, Supporting Information). Here, *θ* refers to the original fabrication angle and is distinct from the dynamic deflection angle (*θ_d_*) induced by magnetic actuation. To assess their effects on hydrodynamic behavior, three discrete values were selected for each parameter. All characterizations were performed in a straight phantom tube with a sufficiently large diameter to eliminate contact and friction between the micro‐fins and the sidewalls (Figure S13 and Note S5, Supporting Information). The measured drag forces, shown in **Figure** **3**a–f, exhibit a positive correlation with increasing flow velocity, as expected. Additionally, increasing either *d* or *θ* results in higher drag forces (Figure 3a–c). When *θ* was fixed at 60°, applying an external magnetic field to open the micro‐fins further enhanced the drag force (Figure 3d–f), indicating stronger hydrodynamic interaction and, consequently, improved steerability under magnetic actuation.

As the diameter of the tube decreases, the micro‐fins tend to come into contact with the sidewalls. This contact introduces friction that resists forward motion and can cause the micro‐fin‐integrated tip to kink under flow drag (Figure S14 and Note S6, Supporting Information). To avoid contact‐induced friction and ensure smooth navigation through confined vascular pathways, the geometric parameters of the micro‐fin‐integrated tip should satisfy the condition 2*d*sin θ < Φ_*Inlet*_. Considering the phantom size (*Φ_Intlet_* = 2.4 mm, selected based on the abdominal aorta artery size of the rabbit) and the goal of minimizing boundary friction, a configuration with *θ* = 45° and *d* = 1.5 mm was selected for further characterization and experimentation.

To further investigate the mechanism of the micro‐fin‐integrated tip, we performed dynamic modeling. We developed a simplified model (Figure 3g) for the micro‐fin‐integrated tip to present its initial state and deformed shape under ***B*** field (Figure 3h) based on the two assumptions. First, the entire tip deforms only within a 2D plane, and second, the elongation of material during the deformation is negligible. The micro‐fin‐integrated tip is discretized into N vertices ***r*** *_i,i = 0,…,N_* and N‐1 material frames ***Q*** *_i,i = 0,…,n‐1_*, defined from the base to the distal end. A discrete micro‐fins on the left side are represented by a set of vertices ***r*** *_(i, j),j = 0,…,N_* and frames ***Q*** *_(i, j),j = 0,…,n‐1_*, defined from the tip to the junction with the stem. Similarly, the right‐side micro‐fins are represented by vertices ***r^’^*** *_(i, j),j = 0,…,N_* and frames ***Q^’^*** *_(i, j),j = 0,…,n‐1_*. Here, *i* denotes the index of the attached vertex, and the governing equations are identical on both sides of the tip stem. The target vector ***t*** is defined as:

(1) $$\begin{array}{c} t_{\left(i,j\to j+1\right)}=\left\{\begin{array}{c} r_{\left(i,j+1\right)}-r_{\left(i,j\right)}j\in \left[0,n-1\right]\\ r_{i}-r_{\left(i,n\right)}j=n \end{array}\right\}\;\mathrm{in}\mathrm{fin}\\ t_{i\to i+1}=r_{i+1}-r_{i}i\in \left[0,N-1\right]\mathrm{in}\mathrm{tip}\mathrm{stem}. \end{array}$$

To better characterize the deformation of the micro‐fins‐integrated tip, we define the normal strain between two adjacent segments of the tip as ***σ*** *_(i, j)_*, as shown below:

(2) $$\sigma_{\left(i,j\right)}=\left\{\begin{array}{c} t_{\left(i,j\to j+1\right)}-d_{\left(i,j+1\right)}j\in \left[0,n-1\right]\\ t_{\left(i,j\to j+1\right)}-d_{i}j=n \end{array}\right\}$$

In this 2D configuration, ***d*** *_(i,j)_* represents the *x‐*axis of transformation matrix ***Q*** *_(i, j)_* in the micro‐fins, and ***d*** *_i_* denotes the initial direction vector of the micro‐fins segment, rotated by the stem matrix ***Q*** *_i_*, as:

(3) $$\sigma_{i}=t_{i\to i+1}-{d^{\prime }}_{i+1}i\in \left[0,N-1\right].$$

In the tip stem region, ***d^’^*** *_i+1_* corresponds to the *y‐*axis in micro‐fins, used in calculating the normal strain along the stem. Based on Kirchhoff–Love theory,^[^ ^47^ ^]^ the governing equation at each vertex of the micro‐fin‐integrated tip can be discretized as:

(4) $$m_{\left(i,j\right)}\frac{dr_{\left(i,j\right)}}{dt}=A_{H}\left({Q_{\left(i,j\right)}}^{T}S_{\left(i,j\right)}Q_{\left(i,j\right)}\sigma_{\left(i,j\right)}\right)+F_{\left(i,j\right)}$$

where *m_(i, j)_* is the mass, ***S*** *_(i, j)_* is the shear/stretch matrix and ***F*** *_(i, j)_* represents the external force acting on segment *(i, j)*. This external force is assumed to originate from fluid thrust, defined as:

(5) $$F_{\left(i,j\right)}=C_{\mathrm{pf}}h_{\left(i,j\right)}v_{f}\left\|v_{f}\times t_{\left(i,j\right)}\right\|$$

Here, *h_(i, j)_* denotes the thickness of each segment, *C_pf_* is the water flow coefficient. *A_H_* is a function used to define the governing equation for each transformation matrix.

(6) $$A_{H}\left(y_{\left(i,j\right)}\right)=\left\{\begin{array}{c} y_{\left(i,0\right)}\mathrm{if}j=0\\ y_{\left(i,j\right)}-y_{\left(i,j-1\right)}j\in \left[1,n\right] \end{array}\right\}$$

And the governing equation of each matrix's rotation is:

(7) $$J_{\left(i,j\right)}\frac{dw_{\left(i,j\right)}^{L}}{dt}=A_{H}\left(B_{\left(i,j\right)}\kappa_{\left(i,j\right)}\right)+\left(Q_{\left(i.j\right)}t_{\left(i,j\to j+1\right)}\times S_{\left(i,j\right)}Q_{\left(i,j\right)}\sigma_{\left(i,j\right)}\right)+C_{\left(i,j\right)}$$

*J_(i, j)_* donates the second moment of inertia of the part, *w^L^_(i, j)_* is the angular velocity of the matrix in the body coordinate system, which equals that in the natural coordinate system under 2D conditions. *B_(i, j)_* is the bending coefficient and *κ _(i, j)_* is the curvature, which can be simplified as:

(8) $$\kappa_{\left(i,j\right)}=\left\{\begin{array}{c} \frac{\theta_{\left(i,j+1\right)}-\theta_{\left(i,j\right)}}{\left\|t_{\left(i,j\to j+1\right)}\right\|}j\in \left[0,n-1\right]\\ \frac{\theta_{i}-\theta_{\left(i,j\right)}}{\left\|t_{\left(i,j\to j+1\right)}\right\|}j=n \end{array}\right\}$$

*θ_(i, j)_* donates the rotation angle of *Q_(i, j)_*, *θ_i_* represents the rotation angle of *Q_i_*. *C_(i,j)_* is the external torque, which corresponds to the magnetic torque in our model, as defined below:

(9) $$C_{\left(i,j\right)}=M_{\left(i,j\right)}\times B$$

In the 2D case, the cross product of vectors reduces to a scalar representing the sign of the torque. At regular vertices of the micro‐fin‐integrated tip, the governing equations are similar to those within micro‐fins itself. However, at connection vertices and transformation matrices, they are formulated as:

(10) $$\begin{array}{ccc} m_{i}\frac{dr_{i}}{dt} & = & A_{H}\left({Q_{i}}^{T}S_{i}Q_{i}\sigma_{i}\right)+{{Q^{\prime }}_{\left(i,n\right)}}^{T}{S^{\prime }}_{\left(i,n\right)}{Q^{\prime }}_{\left(i,n\right)}{\sigma^{\prime }}_{\left(i,n\right)}\\ & & -{Q_{\left(i,n\right)}}^{T}S_{\left(i,n\right)}Q_{\left(i,n\right)}\sigma_{\left(i,n\right)}+F_{i} \end{array}$$

(11) $$\begin{array}{ccc} J_{i}\frac{dw_{i}^{L}}{dt} & = & A_{H}\left(B_{i}\kappa_{i}\right)+{B^{\prime }}_{\left(i,n\right)}{\kappa^{\prime }}_{\left(i,n\right)}-B_{\left(i,n\right)}\kappa_{\left(i,n\right)}\;+(Q_{i}t_{i}\times (S_{i}Q_{i}\sigma_{i}\\ & & +\;Q_{i}\left({{Q^{\prime }}_{\left(i,n\right)}}^{T}{S^{\prime }}_{\left(i,n\right)}{Q^{\prime }}_{\left(i,n\right)}{\sigma^{\prime }}_{\left(i,n\right)}-{Q_{\left(i,n\right)}}^{T}S_{\left(i,n\right)}Q_{\left(i,n\right)}\sigma_{\left(i,n\right)}\right)))+C_{i} \end{array}$$

Based on the above dynamic model, the deflection angle of the micro‐fin‐integrated tip can be controlled (Figure 3i). The experimental setup and detailed description can be found in Figure 3j and Note S7 (Supporting Information). When the reference deflection angles of the tip stem were set to be 5° and 10°, the actual deflection angles of the tip reached 5.5° and 9.4° after 4 steps of the dynamic control period with less than 1° steady state error. The full manipulation process is illustrated in Figure 3i and detailed in Movie S2 (Supporting Information). While the primary focus of this study is on the device design, the above modeling provides valuable insight into its dynamics. The control model can be further refined in future work, particularly when the research shifts toward automation.

The proposed micro‐fins can be modularly docked onto the distal end of standard guidewires to enhance steerability. To address the mechanical mismatch between the micro‐fin‐integrated tip and the relatively stiff commercial guidewire core, a stiffness‐gradient magnetic connector was introduced to ensure a smooth mechanical transition (Figures S18, S13, Note S8, and Table S4, Supporting Information). To demonstrate the necessity of this connector, we compared the performance of micro‐fin‐integrated guidewires with (Figure 3k) and without (Figure 3l) the connector. The results clearly show that the connector facilitates smoother navigation through bifurcations.

Four connector designs were proposed, as shown in Figure 3m and discussed in detail in Notes S8,S9, and Figure S17 (Supporting Information). The mechanical behavior of the four designs under external magnetic fields was simulated using Cosserat rod theory^[^ ^47^, ^48^ ^]^ (Figure S18 and Note S10, Supporting Information), and the results showed good agreement with experimental observations (Figure 3n; Figure S19, Supporting Information). Among them, Design 1 exhibited the best performance in turning maneuvers^[^ ^49^ ^]^ (Figure 3o) and successfully navigated the tortuous M4 segment of a human cerebrovascular phantom (Figure S20, Supporting Information). Consequently, Design 1 was selected for subsequent experiments.

### 2.3 Characterization in Vascular Phantom

**Figure 4:**
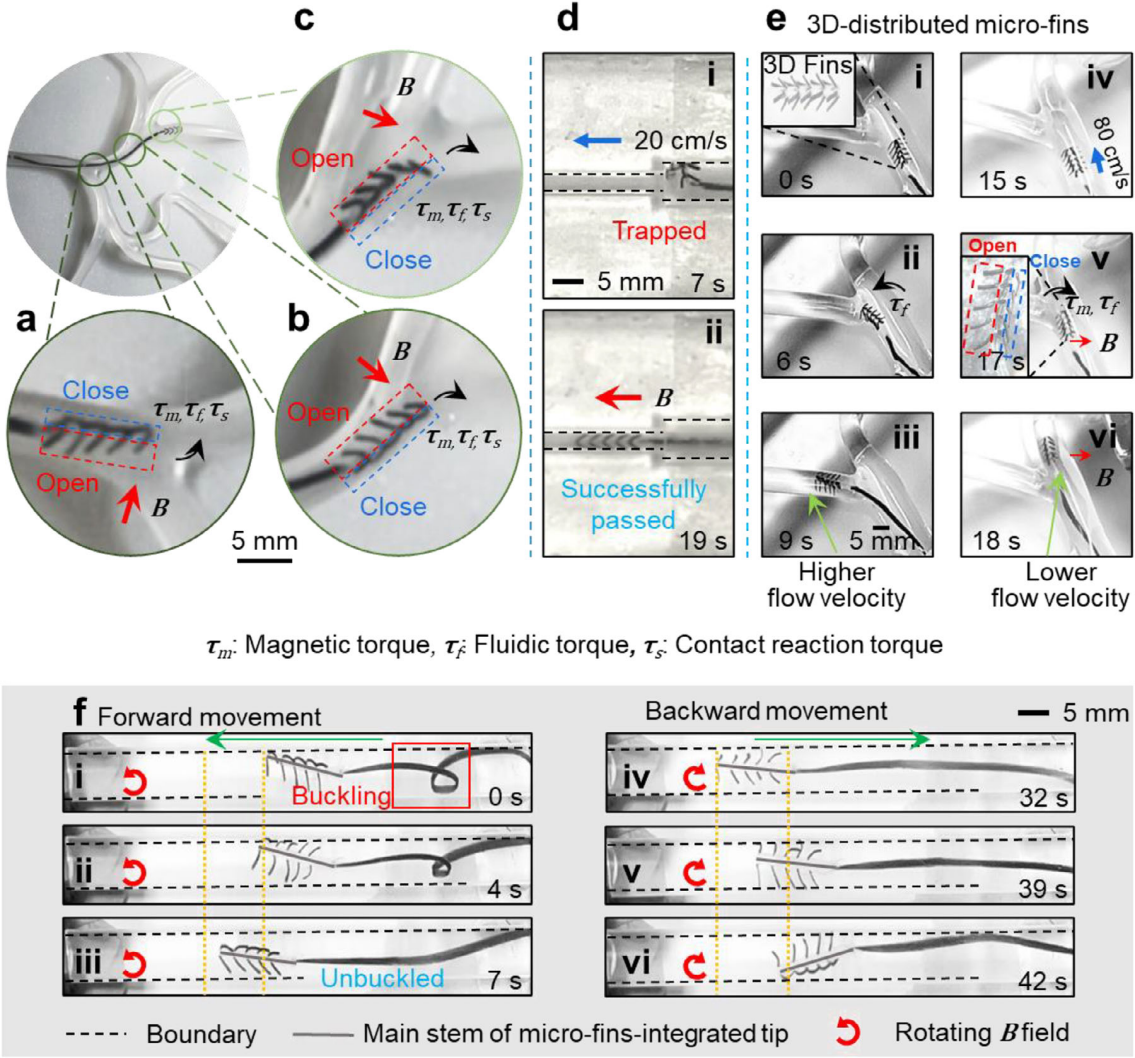
Demonstration of the proposed device in various conditions. a–c) Intervention through tortuous branches in M4 segment of a cerebrovascular model. The proposed device can pass through branches by both the fluidic and the magnetic field. d) The proposed device can go through a tapered tube by closing bilateral micro‐fins. e) 3D micro‐fin‐integrated tip enters the branch with i–iii) higher and iv–vi) lower flow velocity under the guidance of the fluidic field and the magnetic field. f) The proposed device can unbuckle itself by crawling. The detailed illustration of phantoms used is shown in Table S8 (Supporting Information). Phantom 3 is used for (e), and Phantom 7 is used for (f).

Passability of the micro‐fin‐integrated tip was further verified in various conditions. **Figure** **4**a–c shows the experiment in the M4 segment of a cerebral vascular phantom. Micro‐fins were selectively opened or closed to configure the tip into distinct actuation modes at each junction, maximizing the utilization of local flow fields for precise branch selection (Movie S3, Supporting Information). In contrast, commercial guidewires with soft tip failed to traverse tortuous bifurcations due to insufficient structural stiffness and lack of active steering capability (Figure S21, Supporting Information).

Figure 4d‐i,ii shows that the proposed device successfully traversed a tapered tube narrowing from 4.8 mm to 2.4 mm in diameter, with an inlet flow velocity of 20 cm s^−1^. Initially, the micro‐fin‐integrated tip became trapped at the tapered junction due to friction between the fins and the sidewalls (Figure 4d‐i). Upon application of a magnetic field to close both fins into the threading mode, the tip readily passed through the narrowing, as shown in Figure 4d‐ii and Movie S4 (Supporting Information). This result aligned with our previous work on a guidewire equipped with passive, non‐magnetic bristles.^[^ ^40^ ^]^ In that study, long bristles provided better guidance through tapering but tended to get stuck, whereas short bristles reduced friction but lacked sufficient fluidic drag for effective guidance. The bristle length thus had to be carefully tuned beforehand to the narrowing geometry. In contrast, the present design resolves this trade‐off by enabling dynamic adjustment of the effective fin length using a magnetic field. This adaptability allows the guidewire to navigate through tapered regions of varying dimensions without requiring physical modification of the tip.

While a tip equipped with two arrays of micro‐fins can be rotated to align with the bifurcation plane in 3D vascular environments,^[^ ^50^ ^]^ it is also feasible to increase the number of micro‐fins on each side of the tip. As shown in Figure 4e, we fabricated a micro‐fin‐integrated tip with four micro‐fin arrays arranged on four sides. In the absence of an external magnetic field, the tip is guided into the left branch, which has a higher flow velocity (Figure 4e‐i–iii). Upon application of an external magnetic field directed toward the right, the horizontal micro‐fins deform similarly to the 2D case, enabling the tip to steer into the right branch (Figure 4e‐iv–vi).

To fully exhibit the functionality and validate the reproducibility of the proposed micro‐fins on reducing traversal time compared to commercial guidewires, we made the comparisons among micro‐fins, conventional magnetic guidewire, and commercial guidewire (operated without catheters) in cerebrovascular and cardiovascular phantoms as shown in Figures S19–S26 (Supporting Information). The flow velocity of inlets was all set to be 80 cm s^−1,^ and all the comparisons were conducted by Xu Liu (X.L.) under the same conditions, ensuring the guidewire type is the sole variable. The experiments in the cerebrovascular phantom provide the proof of passability under different vascular geometry (Figures S22 and S23, Supporting Information) while the cardiovascular phantom validates the passability and steerability under tortuous vascular pathways (Figures S24 and S25, Supporting Information). A sum of 13 trails was selected for verification. It can be seen that both the micro‐fins and the magnetic guidewire can pass all the trails while the commercial guidewire could only finish 4 out of 13 trails without the assistance of extra pre‐shaped catheters. Meanwhile, owing to the coherence between the magnetic and fluidic actuation for the micro‐fins, it required lower ***B*** field strength and less traversal time compared to a conventional magnetic guidewire. Considering that the commercial guidewire hasn't finished all the trails, the traversal time was evaluated between the micro‐fins and the magnetic guidewire for reference, as shown in Figure S26 (Supporting Information). The traversal time by using the micro‐fins for passing different vascular branches varies from 20.9% to 75.2% of that by using the magnetic guidewire. And in average, the ratio of required time by using micro‐fins to magnetic guidewire is 55.3%, presenting the steerability and ease of use of the micro‐fins.

Finally, the proposed device demonstrates the ability to crawl forward and straighten a buckled soft guidewire (Figure 4f‐i–iii; Figure S27, Supporting Information). In clinical scenarios, once a guidewire becomes buckled within tortuous vasculature, simple retraction followed by reinsertion often fails to restore its trajectory, as the deformed segment may remain geometrically constrained or mechanically anchored within the vessel, preventing successful re‐advancement.^[^ ^51^, ^52^ ^]^ In contrast, the magnetic micro‐fins enable localized and directional buckling correction through active tip advancement. Furthermore, by reversing the direction of the rotating magnetic field, the tip can move backward (Figure 4f‐iv–vi), enabling bidirectional locomotion (Movie S5).

### 2.4 In Vivo Test in a Rabbit Model

With the assistance of an interventional cardiologist, we tested the proposed guidewire in eight New Zealand white rabbits under digital subtraction angiography (DSA) guidance. All procedures were approved by the Animal Care and Use Committee and the Ethics Committee of West China Hospital, Sichuan University (Approval No. 20240103008). The experimental setup is shown in Figure S28 (Supporting Information). An atherosclerosis model was established (Figure S30 and Note S11, Supporting Information), and representative computed tomography angiography (CTA) and DSA images are provided in Figure S31 (Supporting Information). Six vascular pathways, the left subclavian artery, aorta, superior mesenteric artery, left renal artery, and right and left femoral arteries (Trials 1–6), were selected to evaluate guidewire performance.

The device consists of a micro‐fin‐integrated tip, a stiffness‐gradient magnetic connector, and a commercial guidewire (Terumo Medical Corporation, New Jersey, USA; diameter: 460 µm). Navigating against the blood flow is essential during intervention. In rabbits, blood flows from the heart to distal vessels such as the radial arteries, femoral artery, and cerebrovasculature, as shown in the DSA image of rabbit vasculature (Figure S32, Supporting Information). Clinically, guidewires are typically inserted via the femoral or radial artery and advanced through the aorta to reach the coronary arteries.^[^ ^53^ ^]^ Thus, they must traverse both upstream and downstream without buckling. The proposed guidewire's ability to move against flow was validated in Figure S33, Note S12, and Table S5 (Supporting Information). In one representative case, it completed Trials 2–6, which corresponds to all main arteries of the rabbit, in just 57 s (Movie S6, Supporting Information).

**Figure 5:**
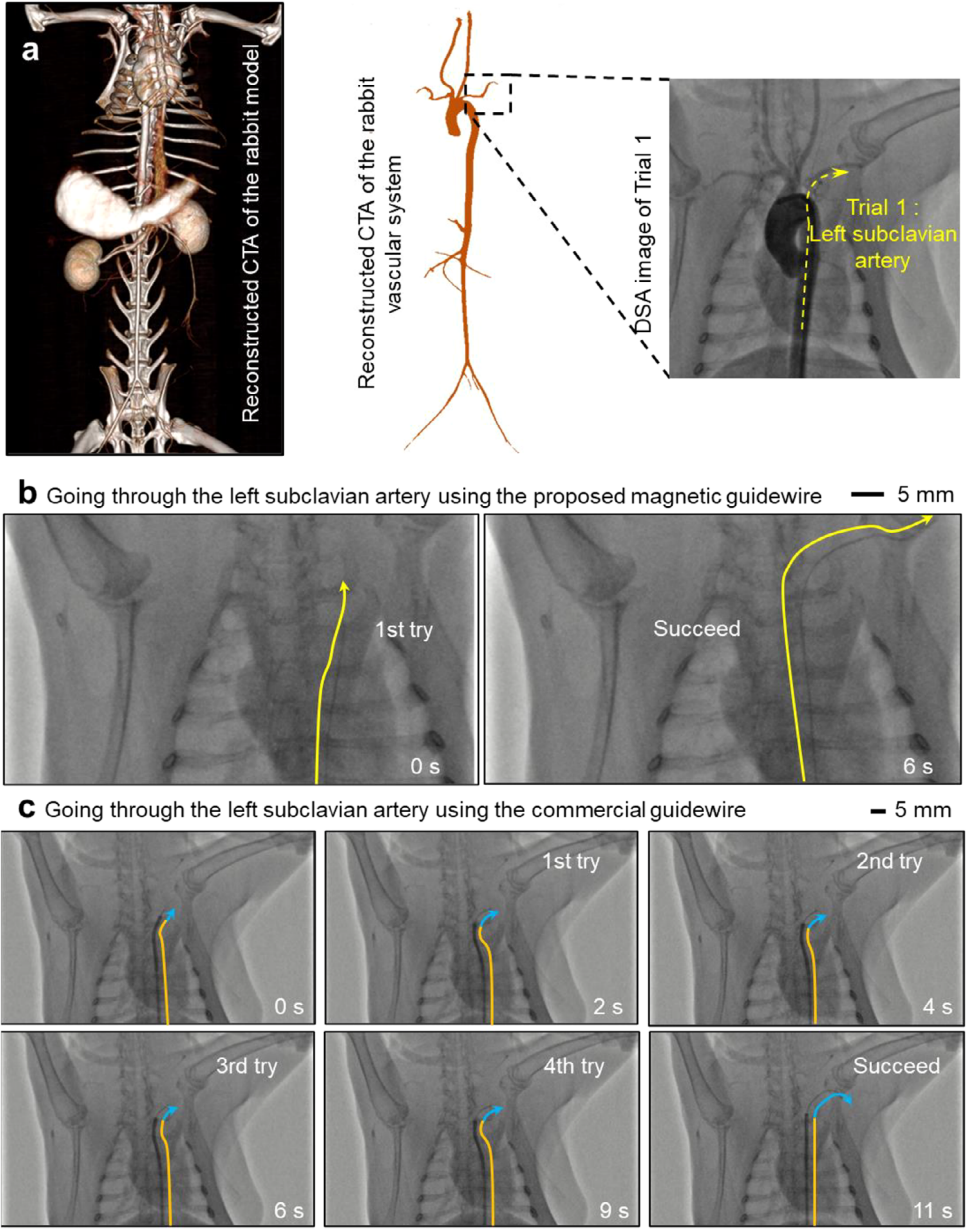
Comparison between the magnetic guidewire with micro‐fin‐integrated tip and commercial guidewire on intervening rabbit's left subclavian artery (Trial 1). a) Reconstructed CTA and DSA images for the left subclavian artery of the rabbit. b) The proposed device had 1 attempt and a total time of 6s. c) The commercial guidewire had 4 attempts and a total time of 11 s.

In Trial 1 (**Figure** **5**; Movie S7, Supporting Information), the proposed guidewire advanced from the femoral artery to the left subclavian artery (Figure 5a). We compared this with the conventional approach using a pre‐shaped catheter and commercial guidewire. The proposed device autonomously located the branch through fluidic guidance and succeeded on the first attempt, completing the task in 6 s (Figure 5b). In contrast, the conventional method required manual rotation by an experienced cardiologist to align the catheter with the subclavian artery. Despite the use of contrast agents, identifying the entry point took multiple attempts. The catheter succeeded only on the fourth attempt, taking 11 s (Figure 5c)—nearly twice the time required by the proposed approach.

**Figure 6:**
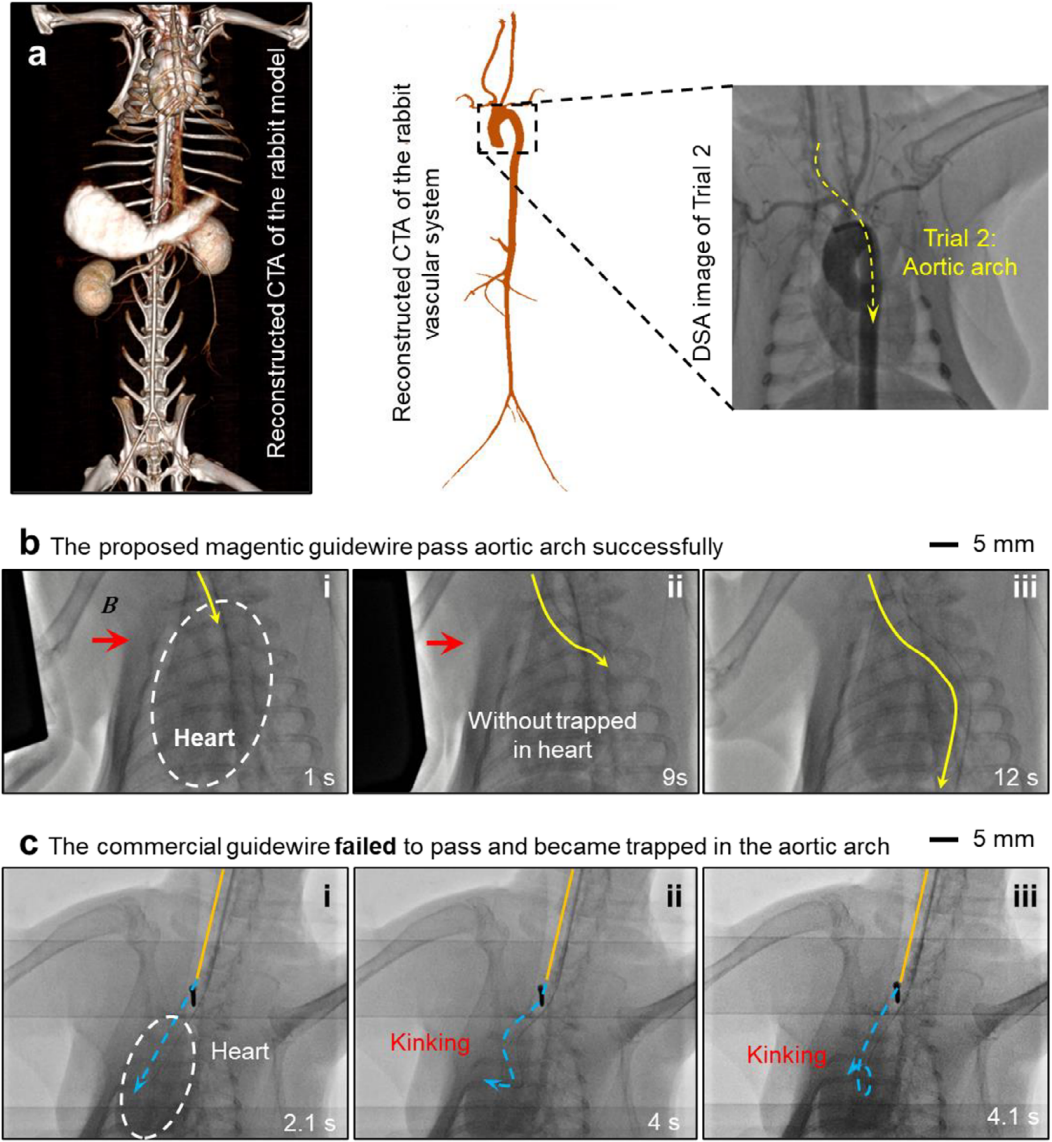
Comparison between the magnetic guidewire with micro‐fin‐integrated tip and the commercial guidewire on passing the rabbit's aortic arch (Trial 2). a) DSA images of the aortic arch of the rabbit. b) The proposed device successfully passed the aortic arch and went along the abdominal aorta under the guidance of the ***B*** field. c) Commercial guidewire failed in passing the aortic arch and was trapped in the rabbit's heart.

In Trial 2 (**Figure** **6**; Movie S8, Supporting Information), the guidewire advanced downward through the aortic arch (Figure 6a). The proposed device successfully traversed the arch under combined magnetic actuation and fluidic guidance in just 12 s (Figure 6b). In contrast, the commercial guidewire became trapped at the arch and failed to advance further (Figure 6c). Its buckled tip impinged on the heart, inducing an artificial heart attack and a sudden spike in heart rate during the attempt.

**Figure 7:**
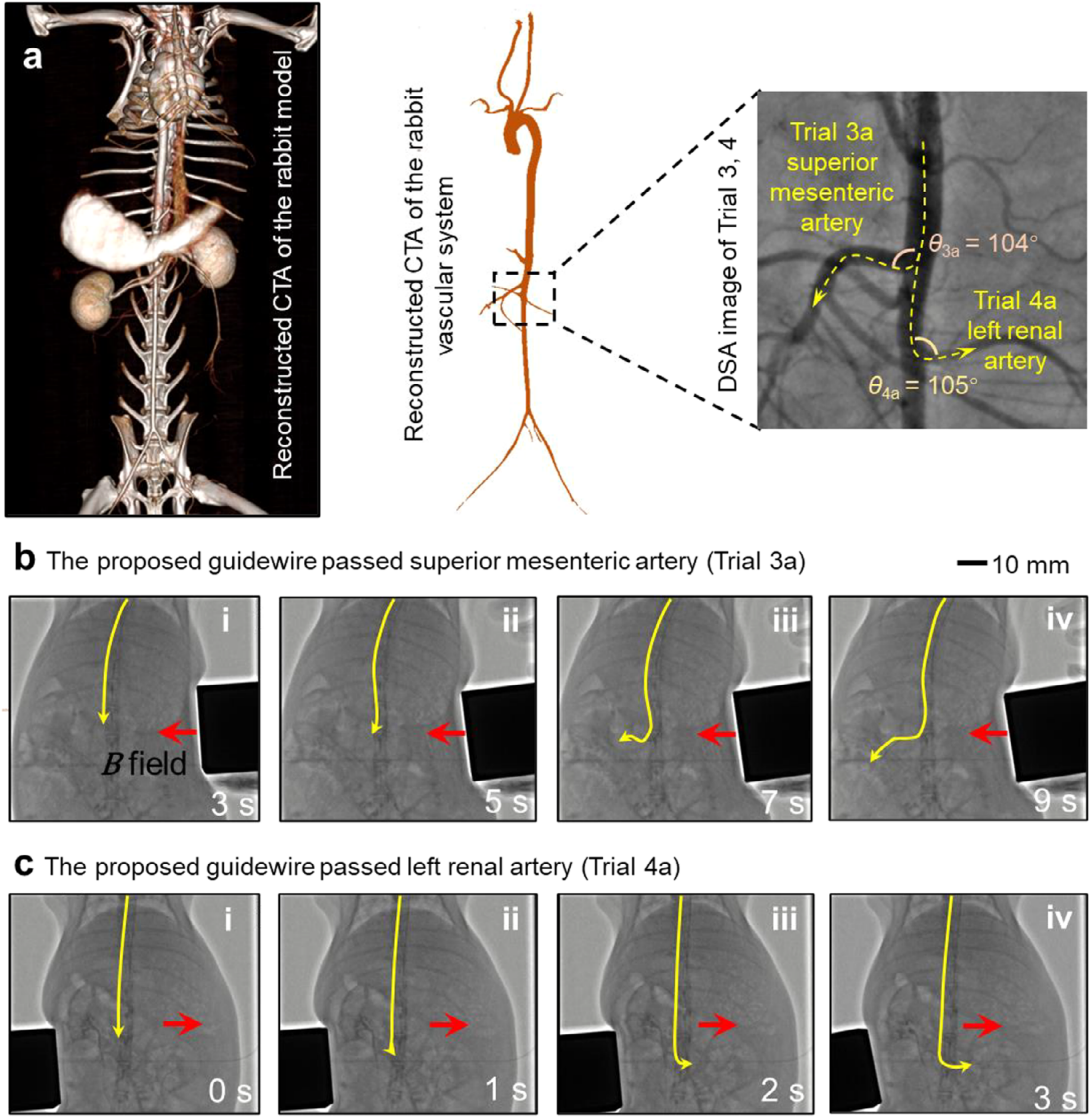
Reaching the superior mesenteric artery and the left renal artery (Trial 3, 4) using a micro‐fin‐integrated tip. a) Original morphology and schematic diagram of the superior mesenteric artery (Trial 3) and the left renal artery (Trial 4) under DSA. The proposed guidewire going through b) superior mesenteric artery (deflection angle: 104°; total time: 9 s) and c) left renal artery (deflection angle: 105°; total time: 3 s).

In Trials 3 and 4 (**Figure** **7**; Movie S9, Supporting Information), the proposed guidewire advanced along the abdominal aorta to the superior mesenteric artery and the left renal artery in the kidney (Figure 7a), after passing the aortic arch. The proposed guidewire successfully reached both the superior mesenteric artery (Figure 7b) and the left renal artery in the kidney (Figure 7c) in 9 and 3 s. It also demonstrates better steerability than conventional magnetic guidewires (Figure S35a,b and Note S13, Supporting Information).

**Figure 8:**
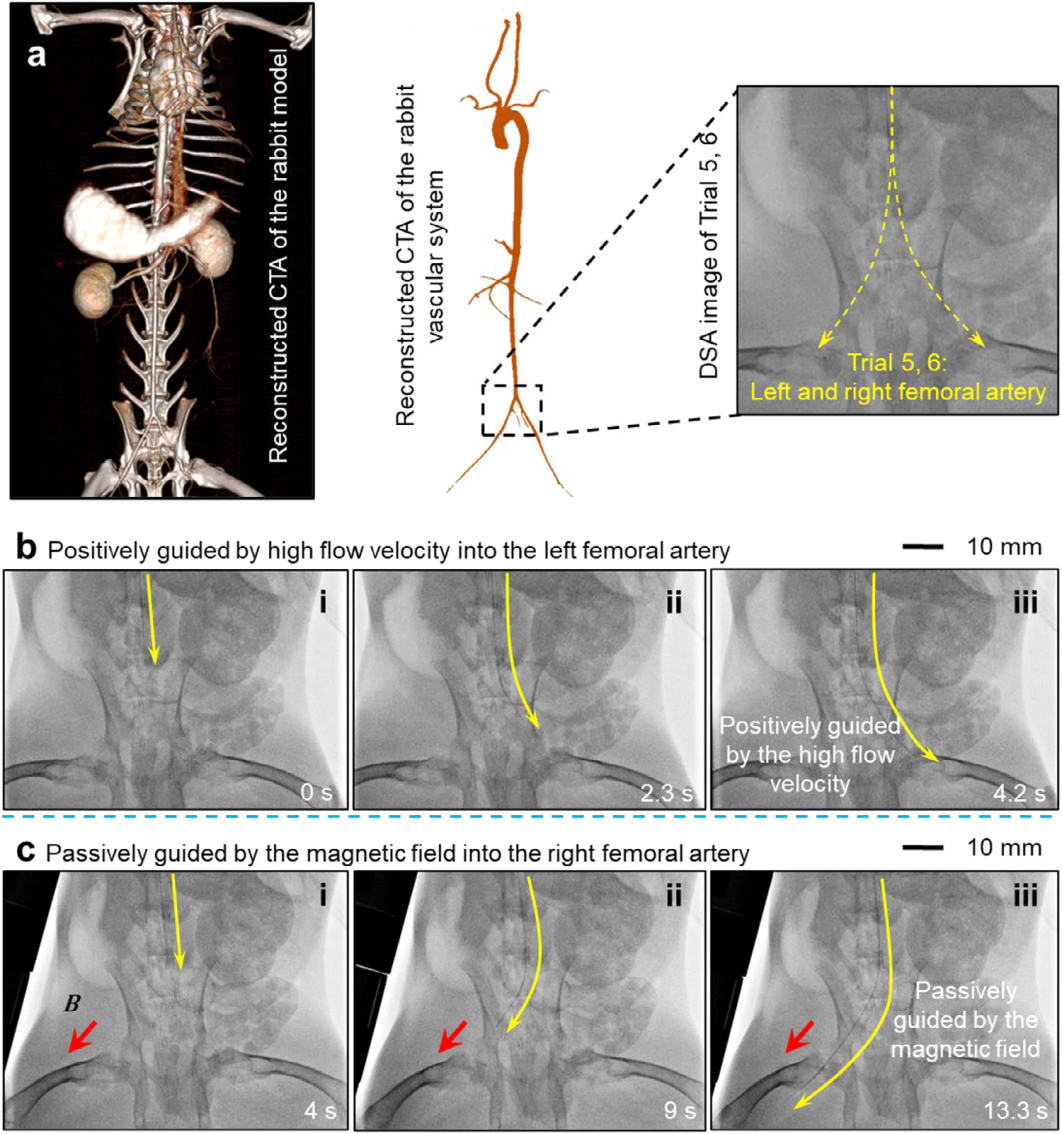
Reaching the femoral arteries (Trial 5, 6) using a micro‐fin‐integrated tip. a) Original morphology and schematic diagram of the rabbit right (Trial5) and left (Trial6) femoral arteries under DSA. b) The proposed device is guided by the fluidic field into the left femoral artery with a higher flow velocity. c) The proposed device is guided by the magnetic field to the right femoral artery with lower flow velocity.

In Trials 5 and 6 (**Figure** **8**, also shown after 1:05 of Movie S6, Supporting Information), the proposed guidewire reached both the left and right femoral arteries. In this experiment, the rabbit model has atherosclerosis in the left femoral artery (note it is the right branch on the Figure 8a; Figure S34 and Table S6, Supporting Information). Ultrasound measurements indicated a significant increase in blood flow velocity (from 50 to 119 cm s^−1^, Figures S3–S5, Supporting Information) in the atherosclerotic vessel compared to the normal vessel. Consequently, at the bifurcation, the proposed guidewire automatically detected the pathway with higher blood flow velocity and entered the right branch, the one with atherosclerosis (Figure 8b). By applying an external magnetic field, the proposed guidewire could also be easily steered into the left branch with lower blood flow velocity, Figure 8c. To further verify the exteroception of micro‐fin‐integrated‐tip on flow velocity, a hemostatic clamp was applied to the midsection of the right branch, reducing its blood flow to zero in the atherosclerotic artery at the bifurcation, while maintaining normal flow in the right femoral artery (Figure S36, Supporting Information). In this scenario, the proposed guidewire automatically went into the right femoral artery. The evaluation of the vascular injury, biocompatibility, and hemocompatibility is shown in Note S14 and Figures S38–S41 (Supporting Information).

## 3 Conclusion and Discussion

In this study, we introduce a bio‐inspired micro‐fin‐integrated tip for multimodal vascular intervention. Unlike conventional magnetic guidewires, the proposed design requires less stringent external magnetic field conditions, due to the additional torques generated by fluidic drag and contact forces against the vessel wall. We characterized and validated the performance of the proposed device using both a medical phantom and a rabbit model.

The limitations of the micro‐fins go to the following two points. First of all, the micro‐fins can utilize both magnetic actuation and fluidic guidance, while this combined effect won't be activated when advance against the flow direction. Second, we mainly focus on the passability and ease of use during interventions, hence, the magnet was manipulated manually during the in vivo test, and the control system can be further developed. Several other strategies can be employed to enhance the performance of the proposed system. First, the current assembly‐based fabrication process can be replaced with precision micro‐molding to form the micro‐fins,^[^ ^54^ ^]^ and the stiffness‐gradient connector can be produced via continuous extrusion of magnetic material with tunable particle loading.^[^ ^28^, ^55^, ^56^ ^]^ Second, the visual sensing potential of the micro‐fins remains underutilized. Notably, the fins are clearly visible under DSA during intervention experiments (Figures 5, 6, 7). Incorporating contrast agents into the fabrication process, as demonstrated in Figure S42 (Supporting Information), can further improve visibility in imaging. This would enable quantitative analysis of micro‐fin deformation as a proxy for local hemodynamic conditions. We have previously developed a physics‐informed neural network (PINN) framework for reconstructing flow fields in complex domains.^[^ ^57^ ^,^ ^58^ ^]^ Using this method, the deformation of micro‐fins could be used to infer local hemodynamics. Finally, although only basic dynamic control was demonstrated in Figure 3i,j, the current modeling framework supports closed‐loop control. The micro‐fin‐integrated tip could be actuated automatically using medical imaging as feedback and a model‐based controller. Thanks to its improved mechanical robustness and reduced reliance on precise external magnetic fields, the device is inherently more stable. This is exemplified by successful navigation using a handheld magnet in a rabbit model (Figures 5, 6, 7, 8). Together, these enhancements could improve the accuracy and reliability of magnetic guidewire‐based vascular access and manipulation during surgery.

## Conflict of Interest

The authors declare no conflict of interest.

## Author Contributions

X.L., Q.L., and Z.C. contributed equally to this work. X.L., Q.L., Z.R., and W.H. proposed and designed the research. X.L. performed the experiments and analyzed the data with the help of W.H. Q.L. performed the in vivo experiments in a rabbit model. Q.L. and H.L. performed the evaluation of the biocompatibility and hemocompatibility. X.C., Z.C., H.W., and Z.R. performed simulations. M.C. and W.H. supervised the research. The manuscript was written by X.L., Q.L., and W.H. with input from all authors. All authors discussed the results and commented or edited the manuscript.

## Supporting information



Supporting Information

Supplemental Movie 1

Supplemental Movie 2

Supplemental Movie 3

Supplemental Movie 4

Supplemental Movie 5

Supplemental Movie 6

Supplemental Movie 7

Supplemental Movie 8

Supplemental Movie 9

## Data Availability

The data that support the findings of this study are available from the corresponding author upon reasonable request.
