# Supplementary material for: Bio‐Inspired Micro‐Fin‐Assisted Multi‐Modal Vascular Intervention
Source: Adv Sci (Weinh). 2025 Nov 27;13(8):e15119. doi: 10.1002/advs.202515119 (PMC12884816; doi:10.1002/advs.202515119)
Supplement: Supplementary file 1 — Supporting Information [file ADVS-13-e15119-s010.docx]

Supplementary Information for

**Bio-inspired Micro-fins-Assisted Multi-modal Vascular Intervention**

Xu Liu^1^*, Qiang Luo^4^*, Zhuoqun Cao^1^*, Hongde Li^4^, Xi Chen^1^, Hong Wang^1^, Mao Chen^4^†, Ziyu Ren^3^†, Wenqi Hu^1,2^†

*Equally contributed; †Corresponding author

**The PDF file includes:**

Notes S1 to S13

Figs. S1 to S30

Tables S1 to S8

Legends for Movies S1 to S9

# Supplementary Note S1. Fabrication of the micro-fins-integrated tip

The design of the magnetically controlled bio-inspired micro-fins enables four shape-morphing modes under the magnetic field, facilitating maneuverability through complex vascular pathways by leveraging both fluidic and magnetic guidance.

The fabrication process of the micro-fins-integrated tip is illustrated in Fig. 1l. First, two mixtures are prepared: mixture A, composed of PDMS and the curing agent in a 10:1 weight ratio, and mixture B, composed of PDMS, curing agent and ferromagnetic NdFeB microparticles in a weight ratio of 10:1:10. Both mixtures are thoroughly mixed to ensure homogeneity. Subsequently, mixture A and B are poured into the central region of the acrylic substrate, respectively. After curing at 80 ℃ for one hour, a 150 µm-thick film of the matrix material is obtained. Laser cutting is employed to fabricate stems and micro-fins in different sizes (Table S3). The micro-fins and stem are integrated into micro-fins tips using silicone paste. Finally, the micro-fins-integrated tip is uniformly magnetized along its stem axis using a vibrating sample magnetometer (VSM) in a consistent magnetic field of 1.8 T, ensuring optimal magnetic responsiveness for its medical applications.

# Supplementary Note S2. FSI simulation of the micro-fins-integrated tip

CTA data and specific clinical records of rabbits were provided by West China Hospital of Sichuan University. The 3D Y-shaped phantom model was constructed based on the CTA data of rabbits’ femoral arteries by using SolidWorks (version 2024, *Φ_Intlet_* = 2.4 mm, *Φ_Outlet1_* = 2.0 mm, *Φ_Outlet2_* = 1.5 mm). The original state of the micro-fins and two morphing modes (left open and right open) under ***B*** field, along with a straight rod for comparison as the commercial guidewire, referred to as mode (i) to (iv) were simulated in the tube with 0.70 mm in front of the bifurcation.

The fluid domain mesh was constructed using unstructured elements in the ANSYS Mesh component (version 2024 R1). The element size was set to be 0.15 mm. The boundary layer mesh consisted of 10 layers, with a growth rate of 1.1 and a transition ratio of 0.3. The model mesh for the original state consists of 791,538 unstructured elements. In this FSI analysis, several assumptions were made to simplify the computational model without compromising the accuracy of the results. The flow within the model was considered incompressible, laminar and Newtonian^1,2^. The governing flow equations are as follows:

$\rho(\frac{\partial u_{f}}{\partial t}+(u\cdot\nabla)u)+\nabla p-\mu\Delta u=0,$ (1)

$\nabla\cdot u=0,$ (2)

where ***u***, *p*, *ρ* and *µ* represent the fluid velocity vector, pressure, density (1.00 g/mL), and dynamic viscosity (0.01 dyne·s/cm²), respectively. We set polyethylene as the material to ensure the convergence of the simulation process while maintaining other properties of the matrix material used in actual fabrication. In small deformations (where the tensile stress does not exceed the yield point), polyethylene typically exhibits nonlinear elastic behavior^3,4^. The Neo-Hookean model was used to describe the material’s stress-strain relationship^5,6^. The governing equations of the device deformation are as follows^7,8^:

 (3)

$W=\frac{C_{1}}{2}(\overline{I_{1}}-3),$ (4)

where *ρ_s_* is the density of the solid, ***u*** represents the displacements of the solid, ***b*** is the body forces applied on the structure, $\boldsymbol{\delta}$ is the Cauchy stress tensor, *W* is the strain energy density, $\bar{\boldsymbol{I}_{1}}$ is the first invariant of the modified right Cauchy-Green deformation tensor. *C_1_* represents the material constant, which is equal to the initial shear modulus *G* of the material. The shear modulus *G* is relative to Young’s modulus (*E* = 1.0 Gpa) and Poisson’s ratio (*γ* = 0.42) as shown below^9,10^:

$G=\frac{E}{2(1+\gamma)}.$ (5). The coupling conditions of the interface between the fluid and solid are velocity continuity and force equilibrium^11,12^ as defined by:

$u_{f}=\frac{\partial u_{s}}{\partial t},$ (6)

$n\cdot\delta_{f}=n\cdot\delta_{s},$ (7)

where ***n*** is the normal vector at the interface, $\boldsymbol{\delta}_{f}$ and $\boldsymbol{\delta}_{s}$ are the stress tensors for the fluid and solid, respectively.

# Supplementary Note S3. Simulation of stress between micro-fins-integrated tip and straight rod under fluidic field

Fig. S2 presents the FSI simulation of the micro-fins-integrated tip in a Y-shaped phantom. Four configurations were examined: (i) the initial undeformed state, (ii) unilateral micro-fins opening on the left, (iii) unilateral micro-fins opening on the right, and (iv) a straight rod representing a commercial guidewire for comparison. At the bifurcation, the flow velocity in the smaller outlet (right branch, 1.18 m/s) exceeds that in the larger outlet (left branch, 0.51 m/s), consistent with ultrasound data from a rabbit femoral artery atherosclerosis model (Fig. S3 - Fig. S5). In state (i) and (iv), this velocity distribution results in a notable pressure differential: average pressure in the left branch: -206.41 Pa, right branch: -90.93 Pa (Fig. S6), which induces a fluidic torque (***τ****_f_*) that guides the micro-fins-integrated tip toward the lower-pressure, narrower branch. Additionally, compared to a straight rod, the micro-fins configuration generates greater hydrodynamic drag towards the branch with higher flow velocity (see Fig. S7 and Table S1 for details). In state (ii) and (iii), the half-opened terminal micro-fins are exposed to elevated pressure (left open: 601.66 Pa, right open: 575.74 Pa). In the absence of an opposing micro-fins to counteract the force, the structure deflects toward the opposite side under fluidic torque (***τ****_f_*), enabling flow-guided steerability and directional maneuverability. Furthermore, the opened micro-fins support the lumen wall and exerts a contact reaction torque (***τ****_s_*) that pushes the stem in the opposite direction. Combined with the gradient-induced drag and the magnetic torque (***τ****_m_*), the tip is more effectively oriented toward the desired direction. This coordinated interplay among the fluidic field, magnetic field, and structural motion enhances overall steerability.

According to the FSI simulation, the stress distributions on both sides of the micro-fins and the straight rod were also analyzed. For micro-fins-integrated tip, the differences between stress 1 and 2 on both sides are 1,161 Pa and 10,199 Pa, generating fluidic torque and drag force that effectively guided the tip into the right branch, which is the pathway with a smaller diameter and higher flow velocity. For the straight rod, the difference in stress 2 on both sides is 1,272, providing only one-eighth the drag force of the tip, as shown in Table S1. This design capability of the micro-fins-integrated tip to discern and respond to variations in flow stress not only enhances its passability efficiency but also underscores its potential in advanced medical applications, where precision and adaptability are critical. The ability to sense flow and adjust accordingly allows for more refined targeting, facilitating smoother passability through complex vascular pathways.

# Supplementary Note S4. Identification of primary design parameter

The drag force on an object in a flow field is primarily influenced by the fluid's properties (such as Reynolds number, *Re*, and drag coefficient, *C_D_*) and the geometry of the object. For micro-fins-integrated tip, the thrust on both the main stem and the micro-fins is calculated, and the total force on the micro-fins-integrated tip is derived by summing the drag force on all parts. For the main stem, the frontal area along the flow direction is given by:

$A_{main}=tw_{1},$ (8)

and the resulting thrust is calculated using:

$F_{main}=\frac{1}{2}C_{D}\rho U^{2}A_{main}=\frac{1}{2}C_{D}\rho U^{2}tw_{1}.$ (9)

For the branches, the upstream surface is the projection of the branch's side facing the flow direction; thus, the thrust on them is calculated using:

$A_{Fin}=td\sin\theta,$ (10)

$F_{Fin}=\frac{1}{2}C_{D}\rho U^{2}A_{Fin}=\frac{1}{2}C_{D}\rho U^{2}td\sin\theta.$ (11)

Assuming the micro-fins-integrated tip consists of (*n*) units, the overall force can be calculated using:

$F_{total}=F_{main}+F_{Fin}=\frac{1}{2}C_{D}\rho U^{2}t(w_{1}+2nd\sin\theta).$ (12)

The drag coefficient (*C_D_*) can be obtained from widely available technical table. The force on the object is directly proportional to the flow velocity, the thickness of the micro-fins-integrated tip, the width of the main stem, the number of magnetic micro-fins units, and the angle and length of the branches. In this research, we fixed the micro-fins units and focused on the effects of *d* and *θ*.

# Supplementary Note S5. Experimental setup for testing drag force of micro-fins-integrated tip with different *d* and *θ* under different flow velocity

The experimental setup for evaluating the drag force of micro-fins-integrated tip with varying *d* and *θ* under different flow velocities is illustrated in Fig. S13. A Y-shaped tube (Table S8 “Phantom 1”) was acquired via 3D printing. The micro-fins-integrated tip was attached to a non-stretchable and flexible thread and placed at the distal end of the tube. The lower branch at the adjacent end is connected to a pulsation pump to apply flow fields with different flow velocities. A load cell is used to pull the guidewire at slow, constant speed of 3 mm/min through the upper branch of the tube, assessing its force response in the flow field. The pulling was to ensure better measurement quality.

# Supplementary Note S6. Going through a Y-shaped phantom with a smaller diameter

When the diameter of the phantom was reduced, the micro-fins start to contact the sidewalls. Micro-fins underwent deformation and experienced frictional resistance, resulting in kinking failure during advancement. However, upon application of a magnetic field, the micro-fins can return to their original configuration and were able to advance smoothly along the flow direction within the phantom. At the bifurcation, kinking may happen. Reapplication of the magnetic field restored the orderly configuration of the micro-fins-integrated tip, enabling guidance toward the desired branch, as illustrated in Fig. S14.

# Supplementary Note S7. Control strategy for the deflection angle of the micro-fins-integrated tip

As shown in Fig. 3j, we used a permanent magnet to generate the ***B*** field. The permanent magnet was placed directly under the micro-fins-integrated tip and was rotated by a step motor which was controlled by a PC. The guidewire was put into a glass tube and we used a pulsation pump to create the fluidic field. The glass tube was immersed in water to prevent the photo distortion caused by water refraction. The position and deflection angle of the micro-fins was detected by an industrial camera which was directly placed above the micro-fins-integrated tip. The images were sent to the PC for further processing.

Due to the high actuation response speed of the magnetic system and the relatively lower computational speed, a discrete Nonlinear Model Predictive Control (NMPC) strategy was designed to regulate the locomotion of the micro-fins-integrated tip based on the dynamic model shown in the section 2 of the main text. The entire bending trajectory was discretized into several segments. In each segment, a second-order polynomial fitting was applied to the angular deformation over time, ensuring smooth transitions and stability at the beginning and end of each bending step.

The tip's position and orientation were extracted in real-time using OpenCV-based image processing and then fed into the NMPC controller as the initial state for receding horizon optimization. The control objective includes minimizing: (1) The position, velocity, orientation, and angular velocity errors at the segmented control points along the tip. (2) The final bending angle error of the entire tip. (3) The rate of change in the magnetic field direction.

The optimization problem is solved using the IPOPT nonlinear solver. To compensate for model mismatch and reduce steady-state error, an additional PI feedback loop is implemented as a correction layer to the controller. Under this control framework, the micro-fins-integrated tip can achieve the desired bending angle within a finite number of control steps. We emphasize that this is just a framework for the closed control of our guidewire and further investigation have to be carried out to fully release its potential.

# Supplementary Note S8. Characterization of Young’s modulus

The process of the Young’s modulus characterization is presented in Fig. S19. The specimens could be simplified as a cantilever beam model. This modeling approach assumes that one end of the specimen is fully constrained at the anchor point, while the opposite, distal end is left free to deflect under applied forces. The applied external force, the length, Young’s modulus, sectional moment of inertia and deflection of the specimen are assigned as *P*, *L*, *E*, *I* and *y(x)*. The total length of the beam is maintained consistently at 𝐿 = 20 mm for all specimens. The deflection differential equation is defined as:

$\frac{d^{2}y(x)}{dx^{2}}=-\frac{M(x)}{E(x)I(x)},$ (13)

where *M(x)* is the torque, which is equal to *Px*). Furthermore, the deflection of the distal end is shown as:

$y(L)=\frac{PL^{3}}{3EI}.$ (14)

The moment of inertia is the moment of inertia of the mass distributed on the section about an axis. For a small area element *dA* at a distance *y* from the neutral axis, the moment of inertia *I* is defined as:

$I=\int y^{2}dA.$ (15)

For a circular section with radius *r*, we calculate its moment of inertia about the neutral axis (the horizontal axis passing through the center of the circle). We use the polar coordinate system to describe a circular section, with the center of the circle as the origin and the radius as *r*. The infinitesimal element *dA* can be expressed as:

$dA=rdrd\theta, y=r\sin\theta_{p},$ (16)

*r* is the distance from the center of the circle to the tiny area element, and *θ_p_* is the polar angle. Finally, the moment of inertia *I* can be calculated by:

$I=\int_{0}^{2\pi} \int_{0}^{r} (r\sin\theta_{p})^{2}rdrd\theta_{p}=\frac{\pi}{4}r^{4}.$ (17)

Hence, the corresponding Young’s modulus and Stiffness can be calculated according to the displacement load curve as:

$E=\frac{PL^{3}}{3Iy(L)},$ (18)

$S=\frac{P}{y(L)}=\frac{3EI}{L^{3}}.$ (19)

# Supplementary Note S9. Fabrication of the stiffness-gradient magnetic connector

To improve stiffness compatibility between the commercial guidewire and the micro-fins-integrated tip, a stiffness-gradient magnetic connector was designed. The fabrication process of Design 1 is shown in Fig. S17. The connector consists of a silicon-PTFE monofilament core and an outer coating made of a PDMS-NdFeB composite. Four different connector designs are presented in this study (Fig. 3m), each incorporating silicon-PTFE monofilaments of varying lengths. In designs 1 and 2, the ends of the silicon-PTFE monofilament are securely bonded to the guidewire using 401 adhesive to ensure robust mechanical attachment. In contrast, designs 3 and 4 omit this bonding step, representing an alternative assembly strategy. Mixture B, as described in Note S1, is coated onto the silicon-PTFE monofilament, resulting in a final outer diameter of approximately 600 µm. The completed connector is then radially magnetized under a uniform magnetic field of 1.8 T.

# Supplementary Note S10. Description of Cosserat rod theory

The simulation here is based on previous work^13,14^. The connector could be modeled by a center line ***r*** and a series of oriented frames of reference ***Q***, where ***Q*** = (***d****_1_*, ***d****_2_*, ***d****_3_*) and is equivalent to the orthonormal triad of unit vectors, ***Q****^-1^* = ***Q***^T^, ***Q***^T^ is the transposition of ***Q***. Hence, vector *x* represents the nature coordinate system (Eulerian frame) and could described in the convected coordinates (Lagrangian frame) as ***X****_r_* = ***Qx***,

$Q\frac{\partial x}{\partial t}=\frac{\partial x_{r}}{\partial t}+w_{r}\times x_{r},$ (20)

$Q\frac{\partial x}{\partial s}=\frac{\partial x_{r}}{\partial s}+\kappa_{r}\times x_{r},$ (21)

where *t* denotes the time and *s* is the center line arc length coordinate in its current configuration, ***w****_r_* is the rod angular velocity in body-convected coordinate system, denoted as $\boldsymbol{w}_{r}=\boldsymbol{Q}\text{vec}[\frac{(\partial\boldsymbol{Q}}{\partial t})\boldsymbol{Q}]$,vec[***A***] denotes the 3-vector associated with the skew-symmetric matrix ***A***. $\boldsymbol{\kappa}_{r}=\boldsymbol{Q}\text{vec}[\frac{(\partial\boldsymbol{Q}}{\partial s})\boldsymbol{Q}]=(\kappa_{1r},\kappa_{2r},\kappa_{3r})^{T}$ is the generalized curvature in body-convected coordinate system. Using the above equations, we could describe the motion of the rod’s position and local frame as shown below:

$\frac{\partial r}{\partial t}=\upsilon.$ (22)

And then the linear balance of each element in Cosserat rod could be expressed as:

$\frac{\partial d_{j}}{\partial t}=(Q^{T}w_{r})\times d_{j},$ (23)

$\frac{\partial(\rho A\upsilon)}{\partial t}=\frac{\partial(Q^{T}n_{r})}{\partial s}+f,$ (24)

Where $\rho$ is the density of the material. Here we use the relative density of different kinds of magnet connectors. *A* is the relative cross-sectional area. ***n****_r_* is the inner force, which can be described as:

$n_{r}=\left(\begin{matrix} \frac{4}{3}GA(\sigma_{1r}-\sigma^{0}_{1r}) \\ \frac{4}{3}GA(\sigma_{2r}-\sigma^{0}_{2r}) \\ EA(\sigma_{3r}-\sigma^{0}_{3r}) \end{matrix}\right).$ (25)

where *E* is Young’s modulus, *G* is the shear moduli, $\boldsymbol{\sigma}_{r}=(\sigma_{1r},\sigma_{2r},\sigma_{3r})^{T}=\boldsymbol{Q}(\frac{\partial\boldsymbol{r}}{\partial s}-\boldsymbol{d}_{3})$ is the normal strain. $\sigma_{1r}^{0},\sigma_{2r}^{0},\sigma_{3r}^{0}$ are the former normal strain. ***f*** is the unit external magnet force of each element. ***f*** has the following relation:

$f=(gradB)M.$ (26)

Where grad***B*** is the spatial gradient of the applied magnetic field, ***M*** is the magnetization vector of the guidewire magnet connectors. With all above relation, the angular momentum balance could be described as:

$\frac{\partial(\rho Iw_{r})}{\partial t}=\frac{\partial\tau_{r}}{\partial s}+\kappa_{r}\times\tau_{r}+Q\frac{\partial r}{\partial s}\times n_{r}+(\rho Iw_{r})\times w_{r}+c_{r}.$ (27)

***I*** is the relative second moment of the area of the magnetic connectors. $\boldsymbol{\tau}_{r}$ is the inner couple which is shown as:

$\tau_{r}=\left(\begin{matrix} \tau_{1r} \\ \tau_{2r} \\ \tau_{3r} \end{matrix}\right)=\left(\begin{matrix} EI_{1}(\kappa_{r1}-\kappa^{0}_{r1}) \\ EI_{2}(\kappa_{r2}-\kappa^{0}_{r2}) \\ GI_{3}(\kappa_{r3}-\kappa^{0}_{r3}) \end{matrix}\right).$ (28)

*I*_1_, *I*_2_, *I*_3_ are the diagonal of ***a***. ***c****_r_* is the external magnet torque of each element in body-converted coordinate system as defined by:

$c_{r}=Q(M\times B).$ (29)

Based on this formulation, we could simulate the deformation of the connector and the result is shown in Fig. S18.

# Supplementary Note S11. Experimental rabbit model

Atherosclerosis is the primary pathological basis of cardiovascular disease, as illustrated in Fig. S29. A widely accepted animal model for studying atherosclerosis involves the combination of balloon-induced vascular injury and an atherogenic diet in New Zealand White (NZW) rabbits, which typically results in the formation of macrophage-derived foam cells in response to high cholesterol and saturated fat intake.

The procedure for establishing the animal model is depicted in Fig. S30. Male NZW rabbits, initially weighing 2.0–2.5 kg, were housed at the Animal Center of West China Hospital. All animals were fed a high-fat diet containing 10% lard, 10% egg yolk, 5% sucrose, and 0.5% cholesterol for four weeks to induce atheroma formation. One week after initiating the atherogenic diet (AD), balloon-induced endothelial injury was performed in the iliac artery via a saphenous artery cutdown. Induction anesthesia was administered by intramuscular injection of pentobarbital (30 mg/kg). To prevent thrombus formation, heparin was intravenously administered at a dose of 100 U/kg prior to the intervention. Under aseptic conditions, a balloon dilation catheter (Boston Scientific, USA) was retrogradely inserted into the iliac artery. The balloon was inflated with air and withdrawn three times along the length of the iliac artery to create endothelial injury. All balloon injury procedures were performed by the same investigator (L.Q.) to ensure consistency. Following the intervention, rabbits continued the high-fat diet for an additional five weeks to promote the development of atherosclerotic plaques and iliac artery stenosis.

# Supplementary Note S12. Discussion on going against the flow direction between guidewire with (a) magnetic and (b) non-magnetic micro-fins-integrated tip.

Our team previously proposed a non-magnetic micro-fin-integrated tip design^15^, which assisted the guidewire in locating the entry point of chronic total occlusions (CTOs). The guidewire introduced in this study not only accomplishes the same task but also demonstrates significantly enhanced functionality, as shown in Fig. 2. It is worth noting that the earlier non-magnetic micro-fin-integrated tip faced limitations in navigating against blood flow. As illustrated in Fig. S33b and S26, when subjected to an opposing flow velocity of 80 cm/s, the prior design collapsed entirely, resulting in functional failure and limiting its applicability in real clinical settings. In contrast, the current design, featuring both the micro-fin-integrated tip and a gradient-stiffness connector, successfully advanced under the same flow condition without failure, as demonstrated in Fig. S33a.

# Supplementary Note S13. Better steerability of the proposed guidewire than conventional magnetic guidewires

The proposed guidewire successfully reached both the superior mesenteric artery and the left renal artery in 9 seconds and 3 seconds, respectively, under the combined assistance of the flow field and magnetic field (Fig. 7b, c). Although the conventional magnetic guidewire also reached these targets (Fig. S35a, b), it required significantly more time, 11 seconds and 24 seconds, respectively. It is worth noting that the comparison paths differ: in Fig. 7, the proposed guidewire followed a top-down trajectory and went along the flow, whereas in Fig. S35, the conventional guidewire advanced bottom-up and went against the flow. Despite this, the comparison still provides meaningful insights into the steerability of the two designs. Specifically, the top-down paths in Fig. 7b, c involve sharper curvatures than the bottom-up paths in Fig. S35a, b, making the task inherently more challenging. Moreover, the conventional guidewire has relatively small fluidic drag, going against or with fluidic flow does not significantly affect its steerability. Therefore, the proposed guidewire demonstrates superior steerability.

# Supplementary Note S14. Evaluation of vascular injury, biocompatibility and hemocompatibility

To ensure biocompatibility, we encapsulated the micro-fins with Parylene C—an FDA-approved conformal coating for implantable devices that is chemically inert and uniformly free of pinholes, and is also used in drug-eluting systems. In line with these standards, our structures were coated with a 0.5-µm Parylene C layer (Specialty Coating Systems, USA). Comprehensive evaluation of vascular injury (Fig. S38), biocompatibility (Fig. S39) and hemocompatibility (Fig. S40) assessments conducted at West China Hospital confirmed excellent performance in both tests.

**S14.1 Parylene-C coating**

A 0.5 µm Parylene-C overlayer was deposited on the micro-fins via atomic layer deposition (TFS-200, Beneq), providing a gas-phase, conformal, and highly uniform film. Coating integrity and uniformity were verified by scanning electron microscopy (Apreo 2C, Thermo Fisher Scientific), as shown in Fig. S41.

**S14.2 Assessment of vascular injury**

Following euthanasia, arterial segments spanning the distal abdominal aorta to the proximal portions of the left and right femoral arteries were excised and fixed in 4% paraformaldehyde. Tissues were then dehydrated, cleared, embedded in paraffin, and serially sectioned at 5 µm. Sections underwent standard hematoxylin–eosin staining, followed by dehydration, clearing, and mounting. Rabbits without guidewire manipulation served as negative controls. Microscopic evaluation focused on endothelial continuity, integrity of the intima, and evidence of hemorrhage, and revealed no signs of vascular injury.

**S14.3 Biocompatibility**

Cytocompatibility was evaluated using human umbilical vein endothelial cells (HUVECs, ATCC) and mouse monocyte–macrophage cells (J774A.1, ATCC). Cells were maintained in Dulbecco’s Modified Eagle Medium (DMEM, Gibco) supplemented with 10% fetal bovine serum (FBS, Gibco) and 1% penicillin–streptomycin (Gibco) in 10-cm dishes. After expansion, 4 × 10⁴ cells were plated per well in 24-well plates (*n* = 3 per group) and incubated for 72 h at 37 °C in a humidified 5% CO₂ environment. Viability and cytotoxicity were determined using the Live/Dead Cell Imaging Kit (R37601, Thermo Fisher Scientific). Fluorescence micrographs were captured on a Leica DMi8 microscope and quantified with Fiji (ImageJ, NIH). Test groups were co-cultured with the micro-fins; untreated cells served as negative controls, and 20% dimethyl sulfoxide (DMSO, Sigma-Aldrich) was used as a positive cytotoxic control.

**S14.4 Hemocompatibility**

Fresh rabbit whole blood was sourced from Chengdu Dashuo Experimental Animal Co., Ltd. For hemolysis and coagulation assays, samples were drawn into tubes containing 3.2% sodium citrate. Blood for smear preparation was collected in heparinized tubes, while EDTA tubes were used for immune-activation tests. All experiments were conducted as three independent replicates using freshly collected blood.

Hemolysis assay: Four milliliters of whole blood were combined with 5 mL of 0.9% NaCl and centrifuged at 1500 rpm for 15 min. The packed erythrocytes were collected and resuspended in 100 mL of 0.9% NaCl. Micro-fins were incubated with this red blood cell suspension in 1.5 mL EP tubes at 37 °C with gentle shaking (60 rpm). Following incubation, the mixture was gently inverted, and 1.5 mL was transferred to a fresh tube and centrifuged at 3000 rpm for 15 min. Subsequently, 200 µL of supernatant was dispensed into a 96-well plate, and absorbance at 540 nm was recorded using a microplate reader (Synergy LX, BioTek). Experimental samples were co-cultured with the micro-fins; 0.2% (v/v) Triton X-100–treated erythrocytes served as the positive control, and cells incubated without micro-fins served as the negative control.

Coagulation assay: Micro-fins were incubated with 1.5 mL of whole blood in EP tubes at 37 °C with gentle shaking (60 rpm) for 60 min. After incubation, plasma was obtained by centrifuging both test and control samples at 3000 rpm for 15 min, then diluted 1:3 with 0.9% NaCl. Commercial kits for PT, TT, FIB, and APTT (SK0080, SK0010, SK0030, SK0100; Beijing Succeeder Technology) were added, and coagulation indices were read on an automated coagulation analyzer (SF-8050; Beijing Succeeder Technology). Experimental samples contained the micro-fins, while negative controls were processed without them.

Assessment of Immune Activation: To assess micro-fins-induced immune responses, rabbit serum was analyzed for complement fragments C3a and C5a using ELISA kits (JM-00607R1, JM-0075R1; Jingmei Biotechnology). Serum was prepared from whole blood collected in pyrogen-free tubes, allowed to clot at room temperature, and centrifuged at 3000 rpm for 10 min. Samples were allocated to a control group (no robot exposure) and an experimental group (co-incubated with the robot). ELISAs were performed per the manufacturer’s instructions, with absorbance read at 450 nm on a microplate reader. C3a and C5a concentrations were derived from standard curves and compared between groups to evaluate complement-mediated activation.


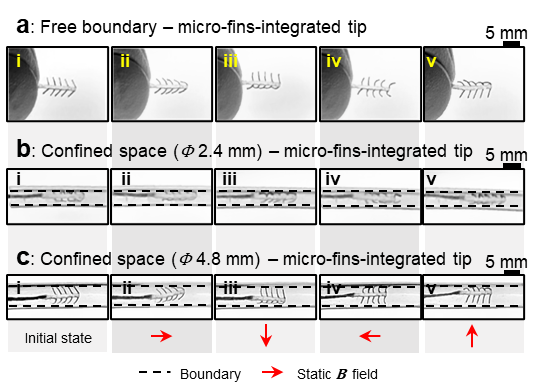


**Fig. S1: Deformation of magnetically controlled bio-inspired micro-fins under *B* field with different boundary conditions. (a)** Deformation of the micro-fins-integrated tip under a free boundary condition. **(b)** Deformation of magnetic micro-fins with a constrained tube, ***Φ*** = 2.4 mm (Phantom 7 in Table S8). **(c)** Deformation of magnetic micro-fins with a constrained tube, ***Φ*** = 4.8 mm (Phantom 2 in Table S8).


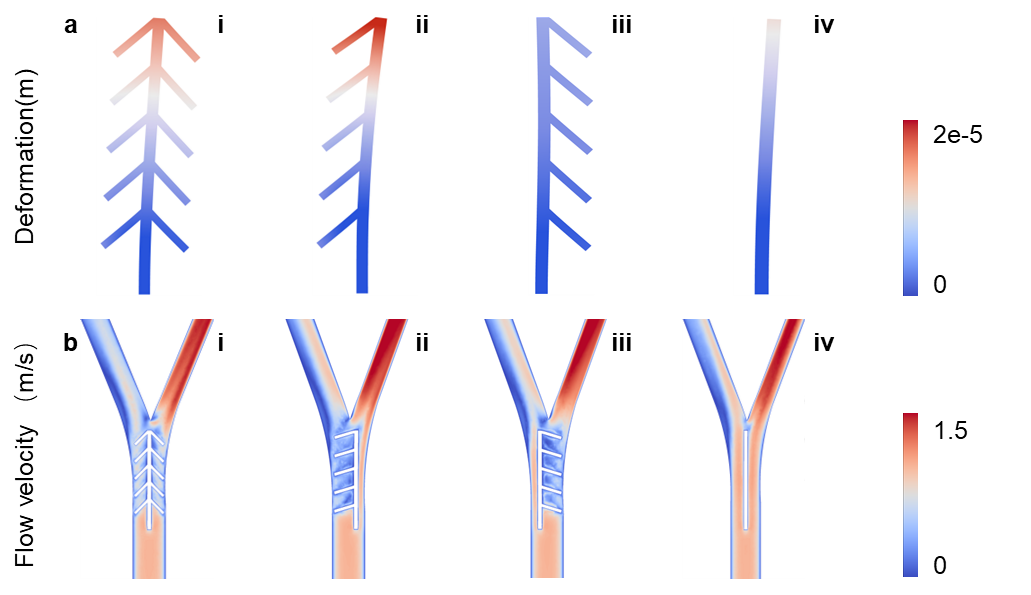


**Fig. S2: Fluid-structure interaction (FSI) analysis. (a)** Deformation and **(b)** flow velocity of the micro-fins-integrated tip (i ~ iii) and a straight guidewire (iv) in a Y-shaped phantom.


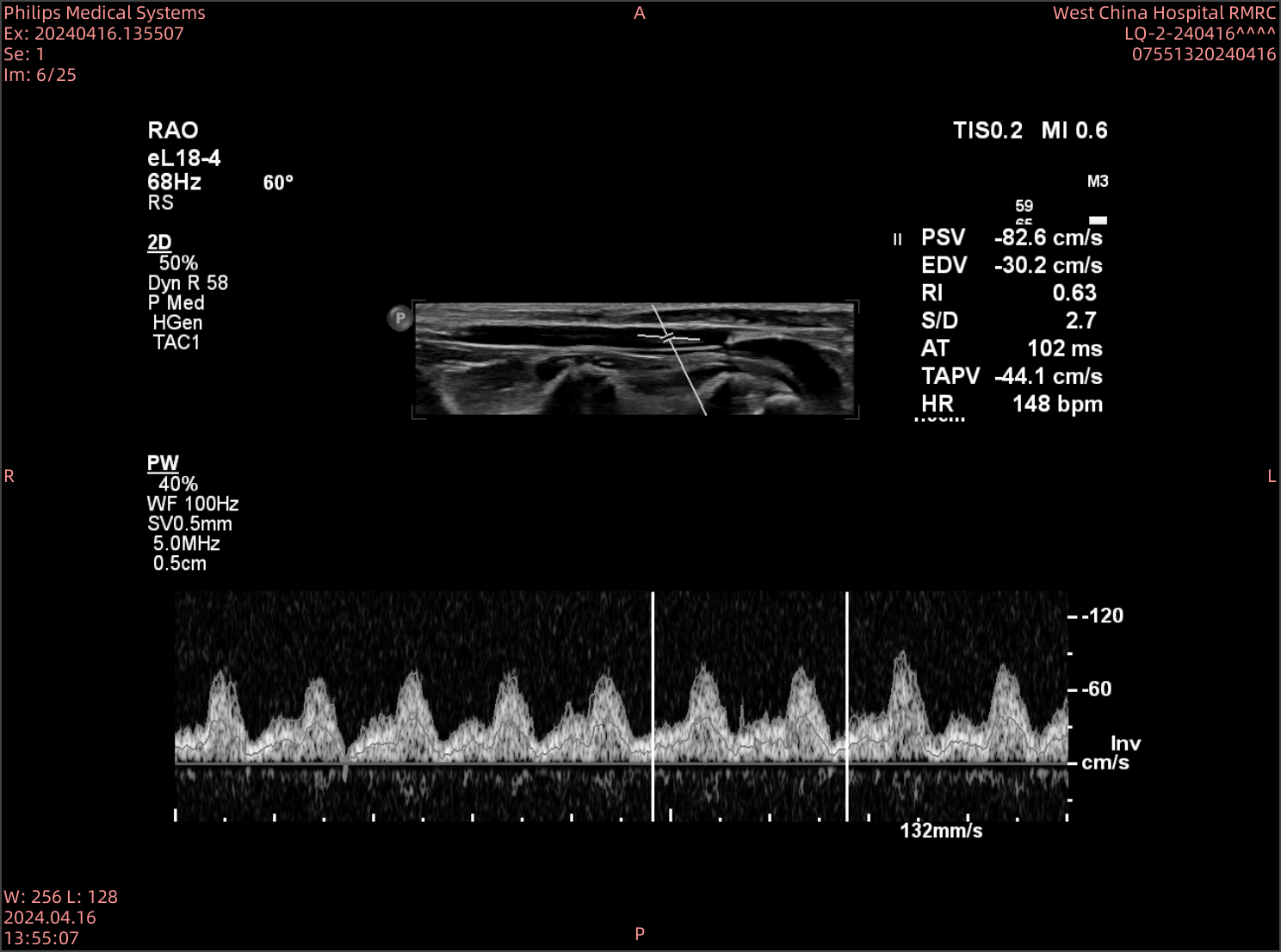


**Fig. S3: Abdominal aorta of the rabbit. The peak flow velocity is 80-90 cm/s.**


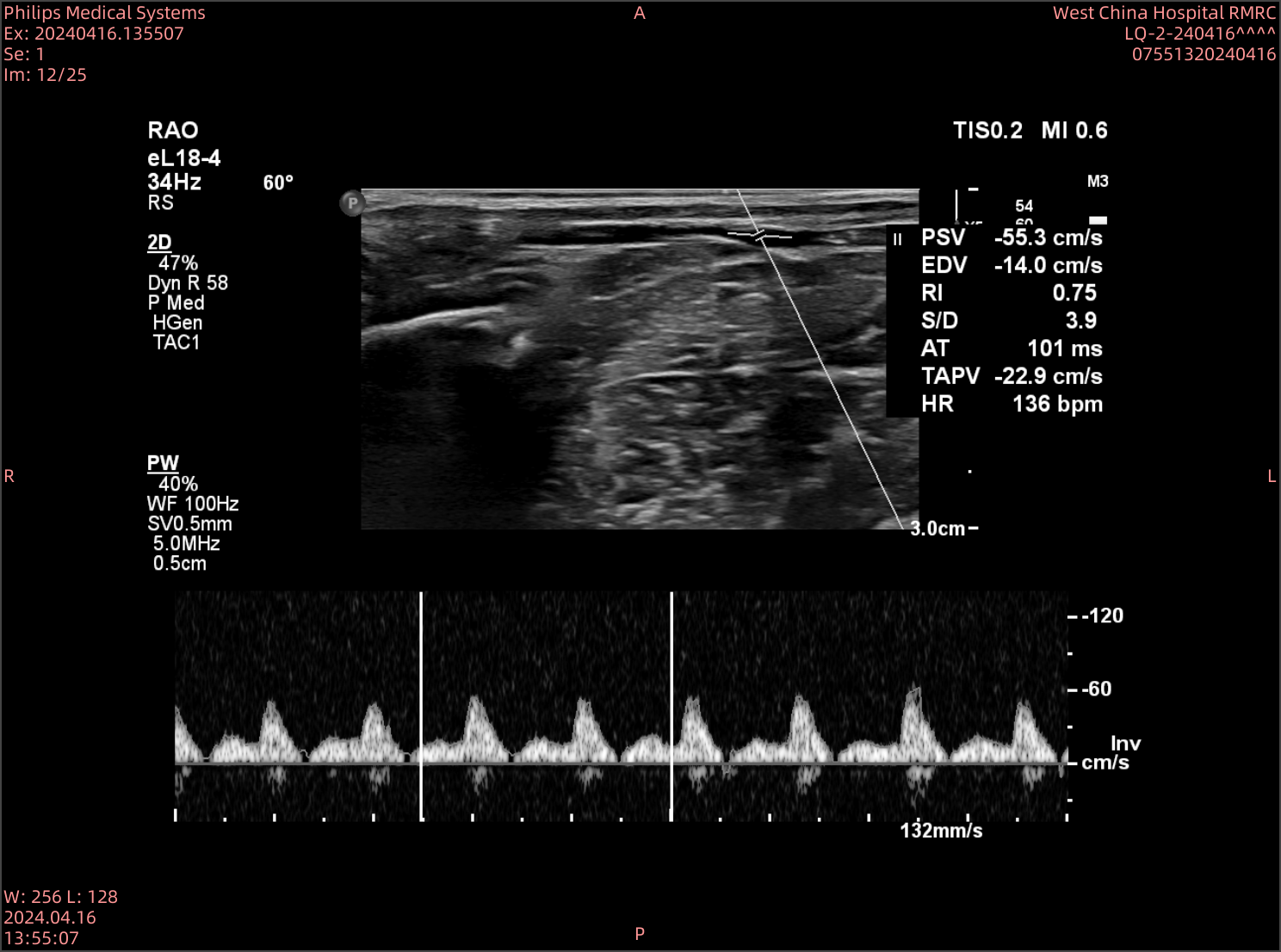


**Fig. S4: Normal femoral artery of the rabbit. The peak flow velocity is 40-50 cm/s.**


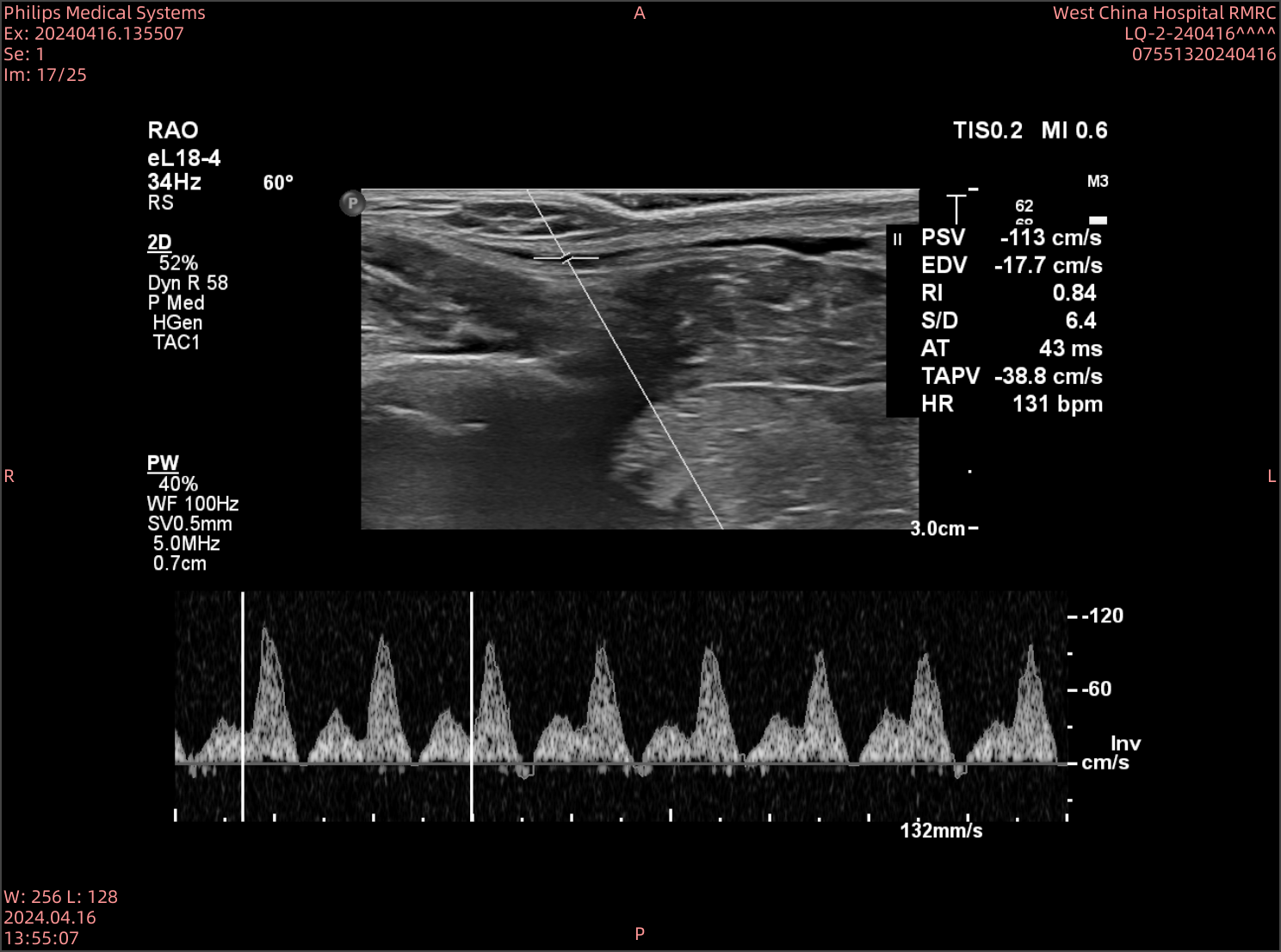


**Fig. S5: Femoral artery with atherosclerosis of the rabbit. The peak flow velocity is 100-110 cm/s.**


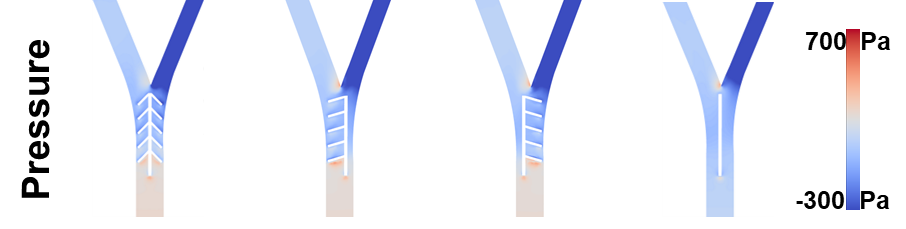


**Fig. S6: FSI analysis of the micro-fins-integrated tip under four actuation modes, focusing on pressure distribution.**

**
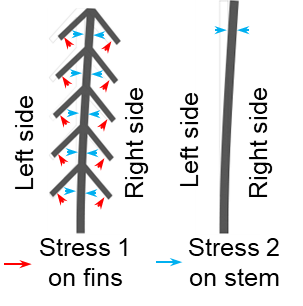
**

**Fig. S7: Comparison of stress between the micro-fins-integrated tip and a straight rod under the fluidic field condition.**

**Table S1 Comparison of stress between micro-fins-integrated tip and straight rod under fluidic field.**

|  | Micro-fins-integrated tip | | Straight rod | |
| --- | --- | --- | --- | --- |
|  | Left side | Right side | Left side | Right side |
| Stress 1 (Pa) | 1035 | 2405 | / | / |
| Stress 2 (Pa) | 3218.3 | 2210.4 | 269.2 | 203.8 |

**
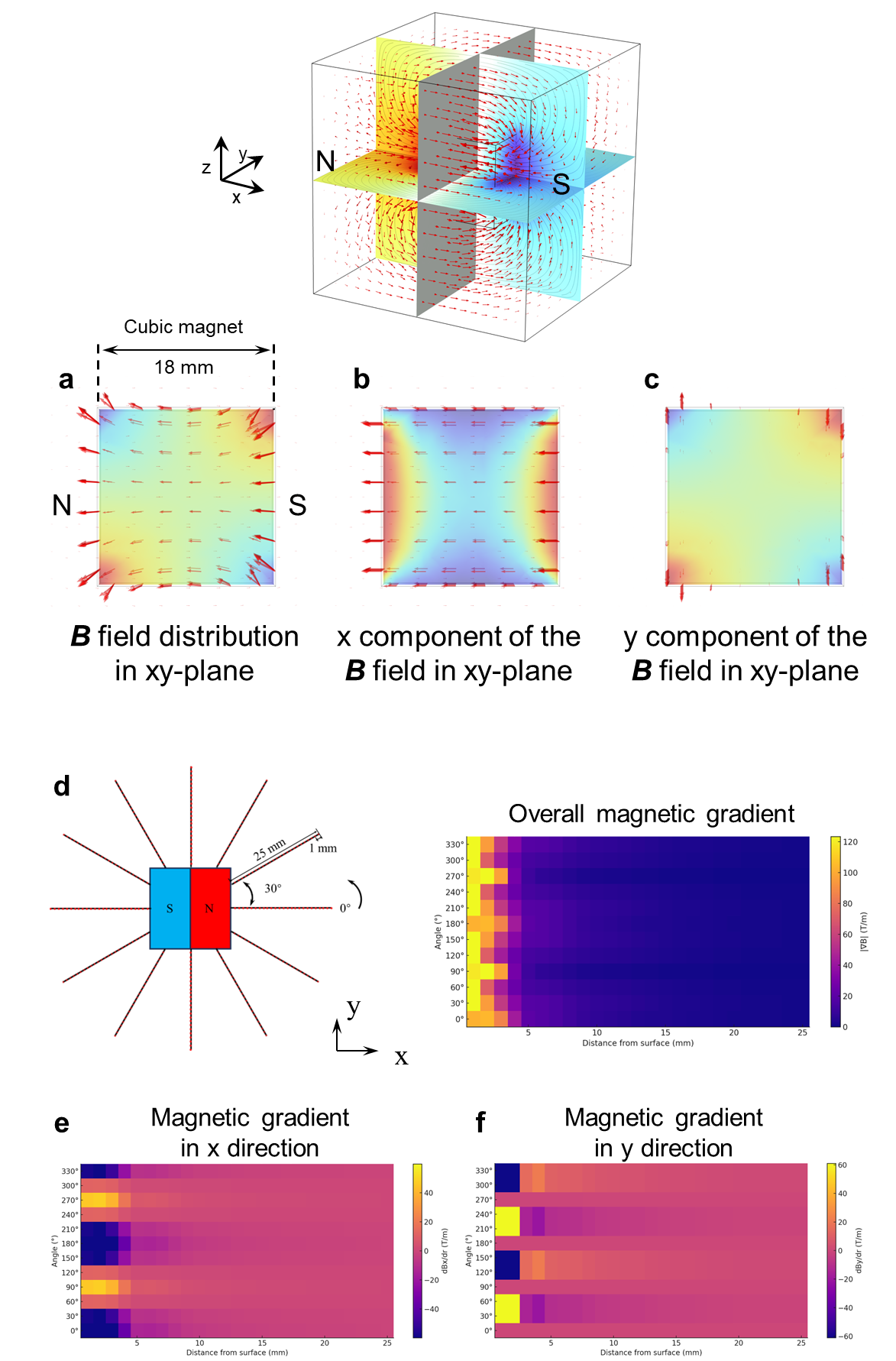
**

**Fig. S8: Magnetic field distribution of the cubic magnet (18 mm×18 mm×18 mm). (a)** Projection of the magnetic field onto the xy-plane. **(b)** x-component of the magnetic field distribution on the xy-plane. **(c)** y-component of the magnetic field distribution on the xy-plane. **(d)** Overall magnetic gradient and component in **(e)** x and **(f)** y direction.

**
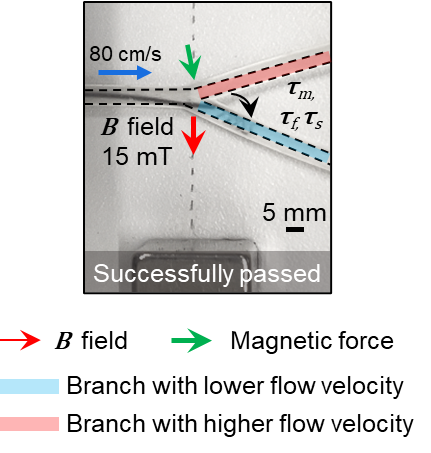
**

**Fig. S9: Going into the lower branch with lower flow velocity.**


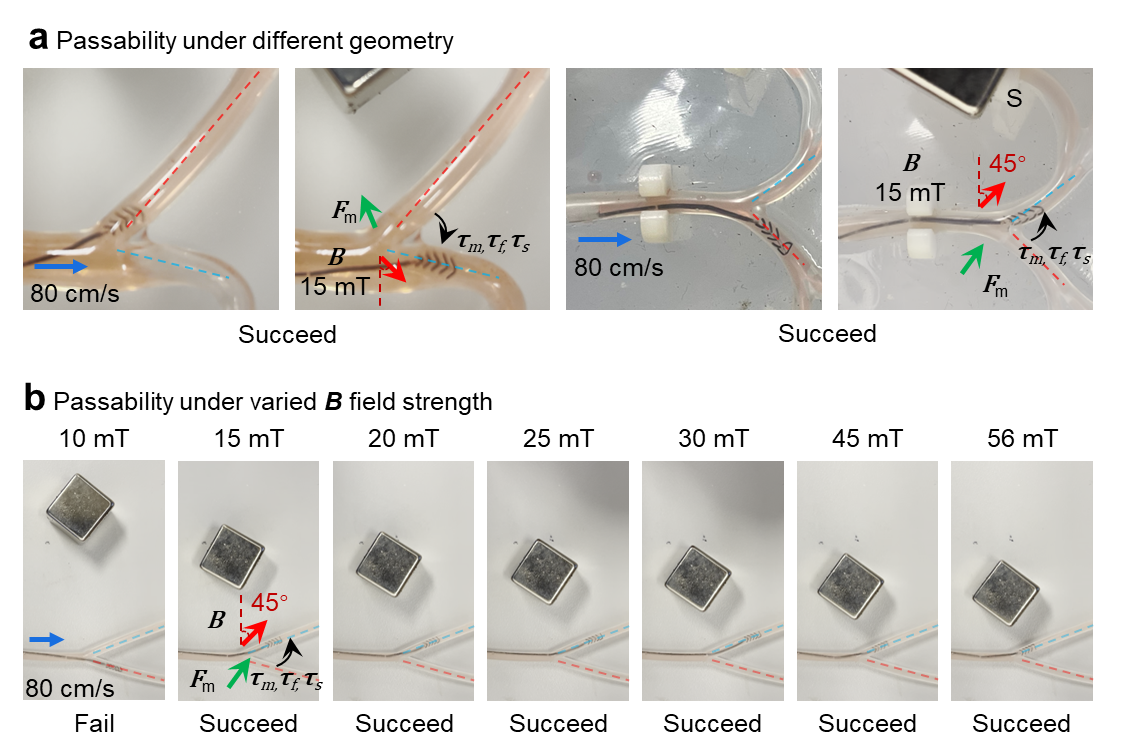


**Fig. S10: Passability of micro-fins under different geometry and *B* field strength.**


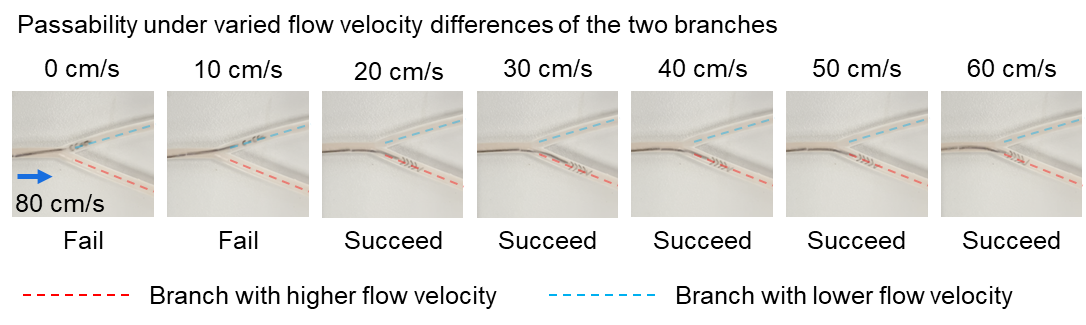


**Fig. S11: Passability under varied flow velocity differences of the two branches.**


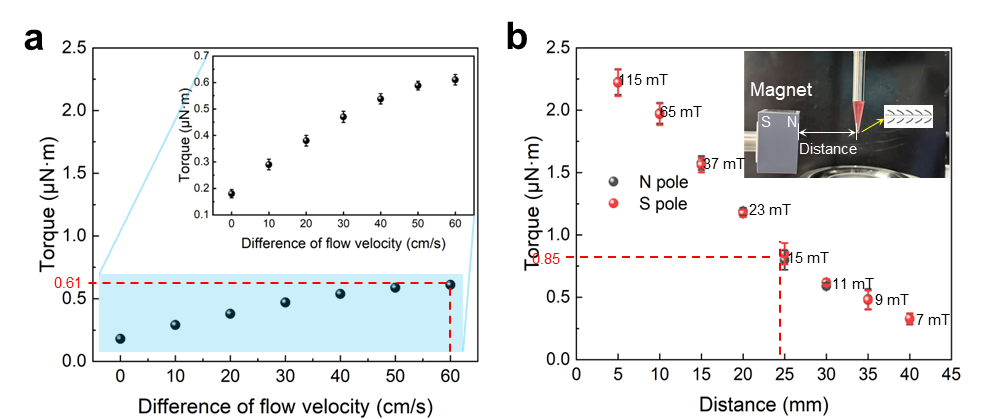


**Fig. S12: Comparisons between torques generated from fluidic field and magnetic field.** Data are presented as mean ± standard deviation with *n* = 5.

**
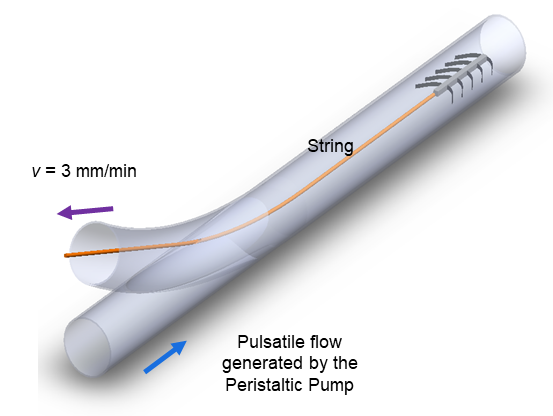
**

**Fig. S13: Experimental setup for measuring the drag force of micro-fins-integrated tip with different *d* and *θ* under different flow velocities.** For better measurement, the guidewire is pulled by a load cell at 3mm/min.


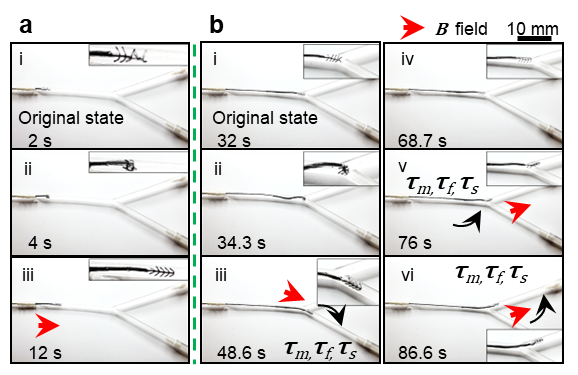


**Fig. S14: Going through Y-shaped phantom with smaller diameter.** (Phantom 4 in Table S8, *Φ_Intlet_* = 2.4 mm, *Φ_Outlet1_* = 2 mm, *Φ_Outlet2_* = 1.5 mm). (a) The micro-fins-integrated tip buckled along the straight pathway without ***B*** field and was restored when ***B*** field applied. (**b**) The micro-fins-integrated tip can buckle at the bifurcation. The configuration could be immediately restored by applying external B field. The tip was subsequently guided into the upper and lower branches.


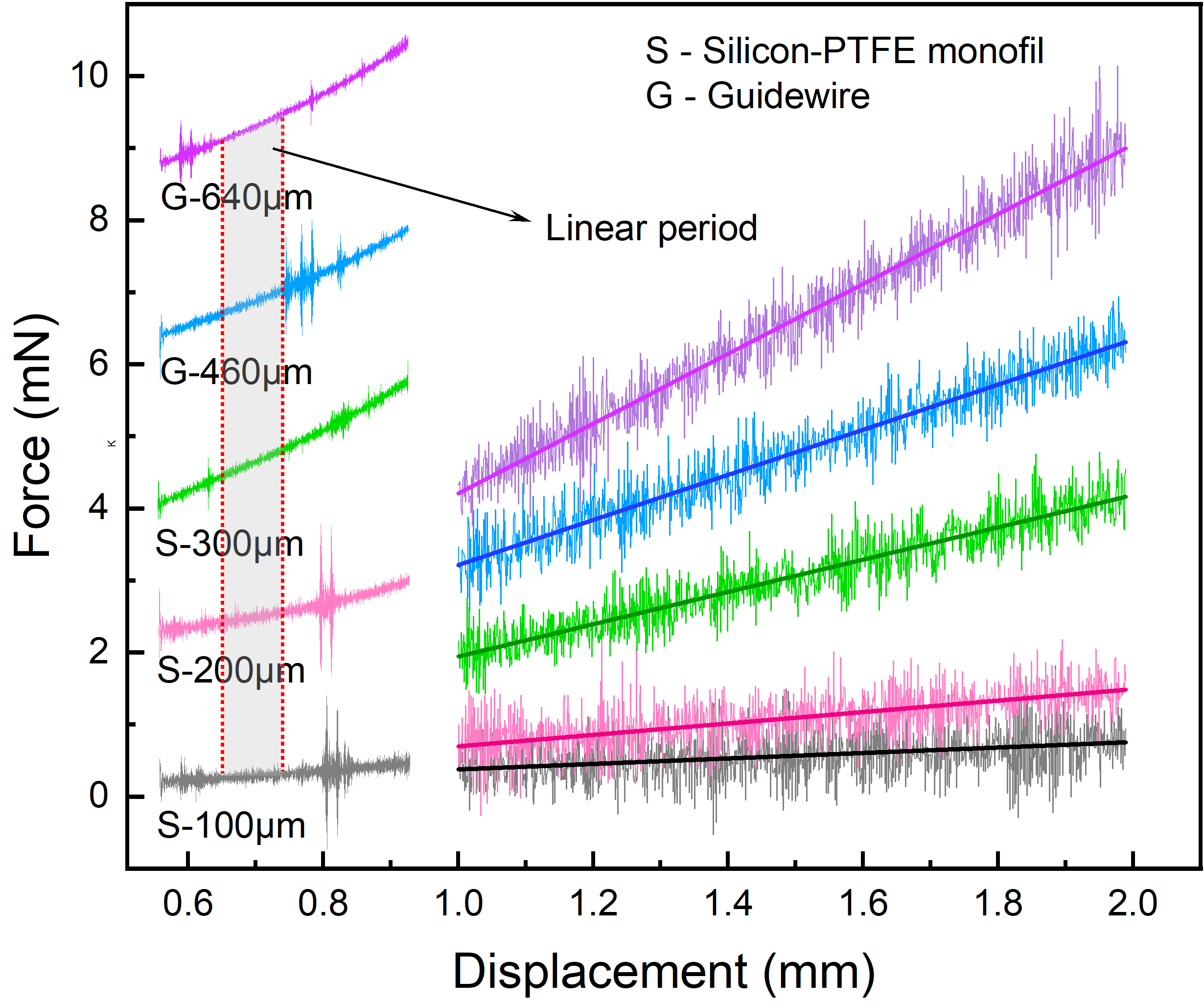


**Fig. S15: The force-displacement curves of silicon-PTFE monofil and guidewire in different diameters. The length of all the specimens was set to be 20 mm.**


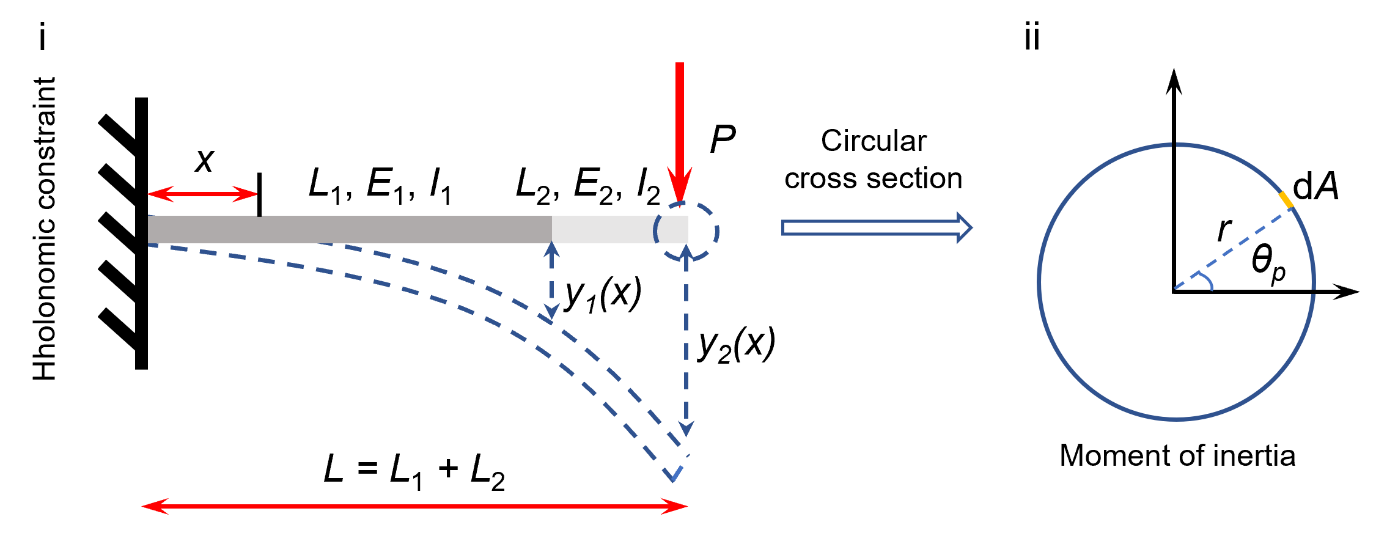


**Fig. S16: Schematic diagram of testing Young’s modulus and Stiffness of silicon-PTFE monofil and guidewire in different diameters.**


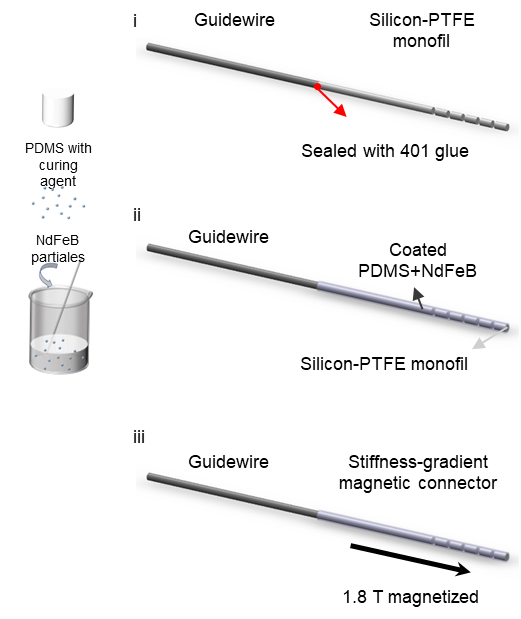


**Fig. S17: Fabrication of the stiffness-gradient magnetic connector.**


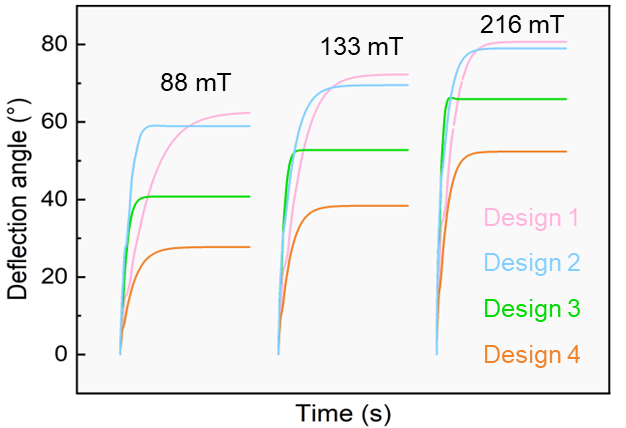


**Fig. S18: Simulation of deflection angles for the above 4 designs under different *B* field (88 mT, 133 mT and 216 mT, corresponding to the stimulating distance of 4 cm, 3 cm and 2 cm).**


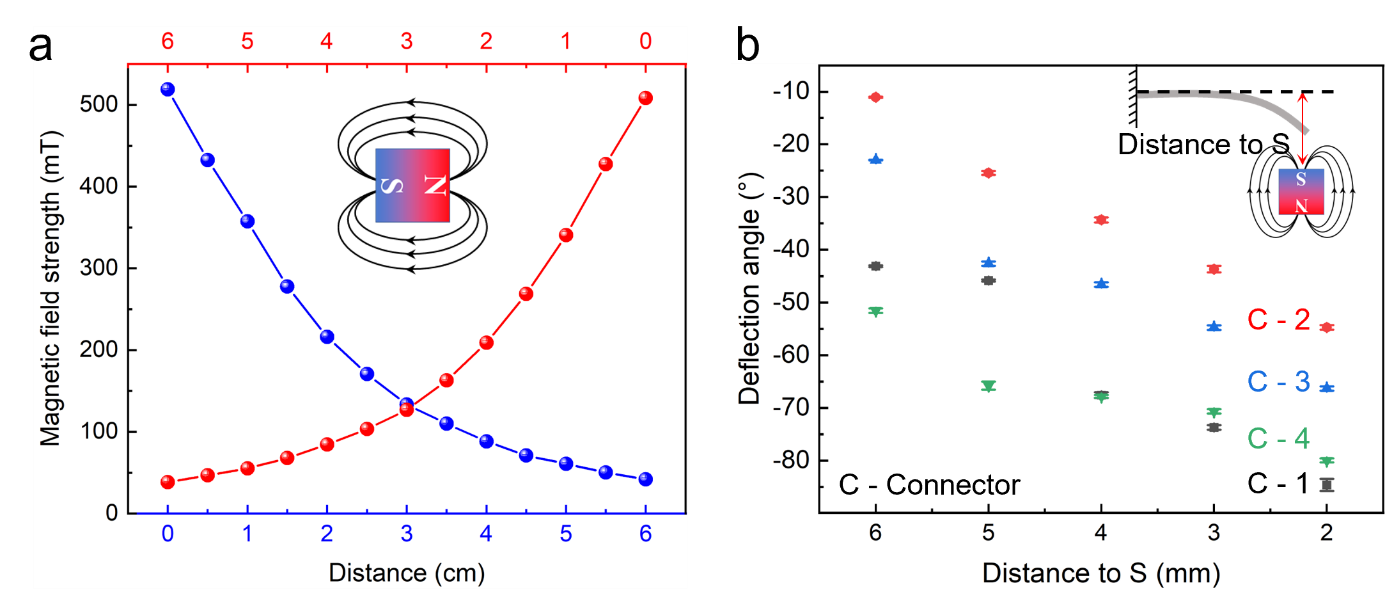


**Fig. S19: Experimental results of deflection angles of all the four designs of stiffness-gradient magnetic connector.** (**a**) Curve of magnetic field strength to distance. When the distance is 2 cm, 3 cm, and 4 cm, the corresponding magnetic field strength are 88 mT, 133 mT and 216 mT. (**b**) The deflection angles of all the four designated stiffness-gradient magnetic connectors. The data in **a**, **b** are presented as mean values ± standard deviation for *n* = 5.


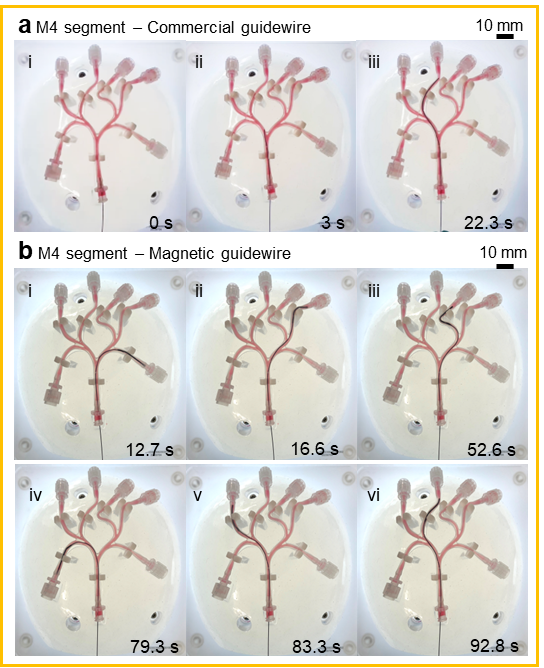


**Fig. S20: *In vitro* experiments of magnetic connector and commercial guidewire reaching M4 segment in a human cerebrovascular phantom.**


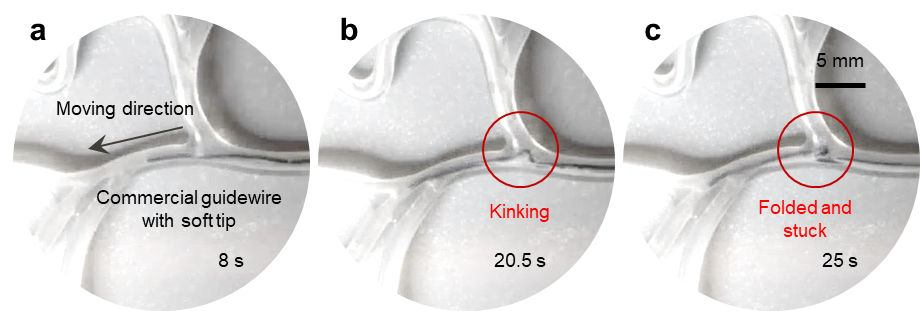


**Fig. S21: Failure to pass the M4 segment using a commercial guidewire with soft tip.** The soft tip buckled at the bifurcation and hindered the advancement of the commercial guidewire.


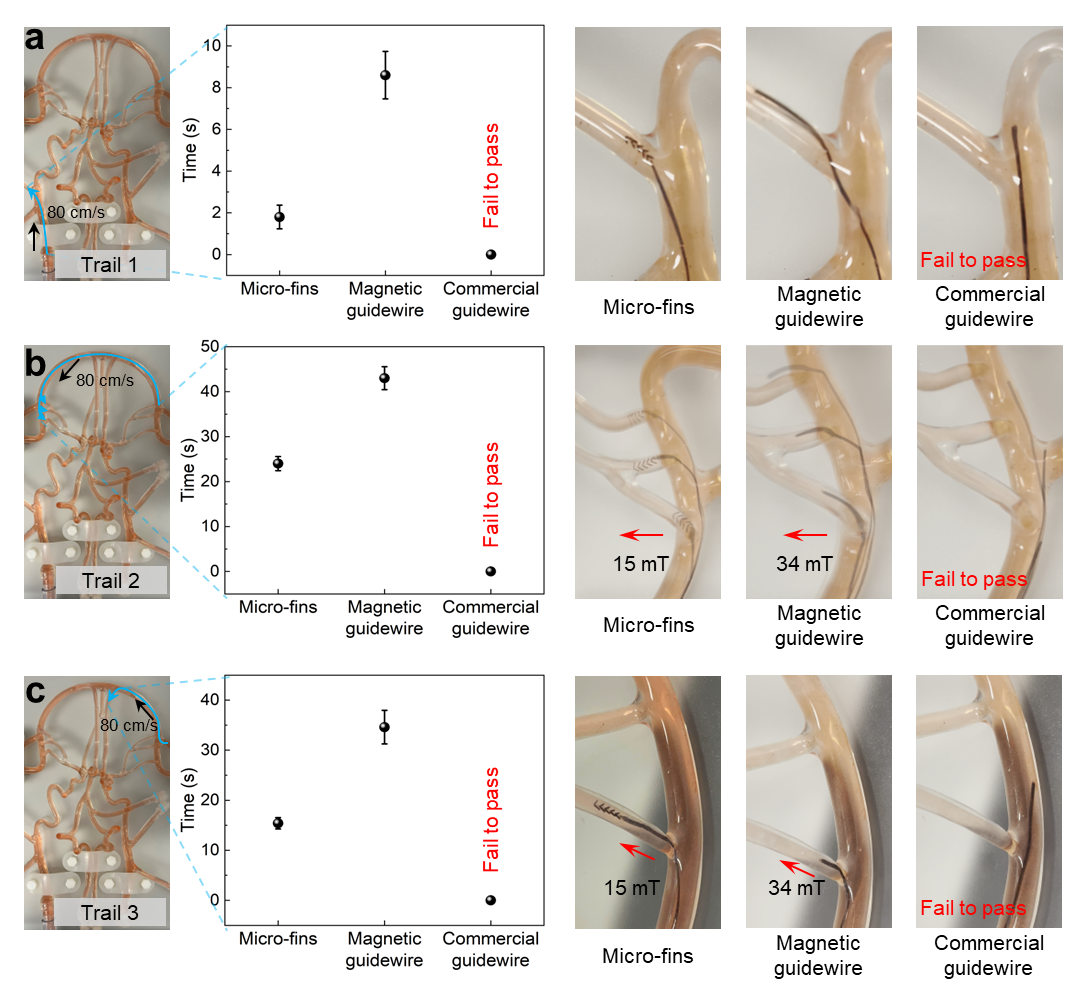


**Fig. S22: Comparison among micro-fins, conventional magnetic guidewire and commercial guidewire on advancing three different target branches of the cerebrovascular model.** Data are presented as mean ± standard deviation with *n* = 5.


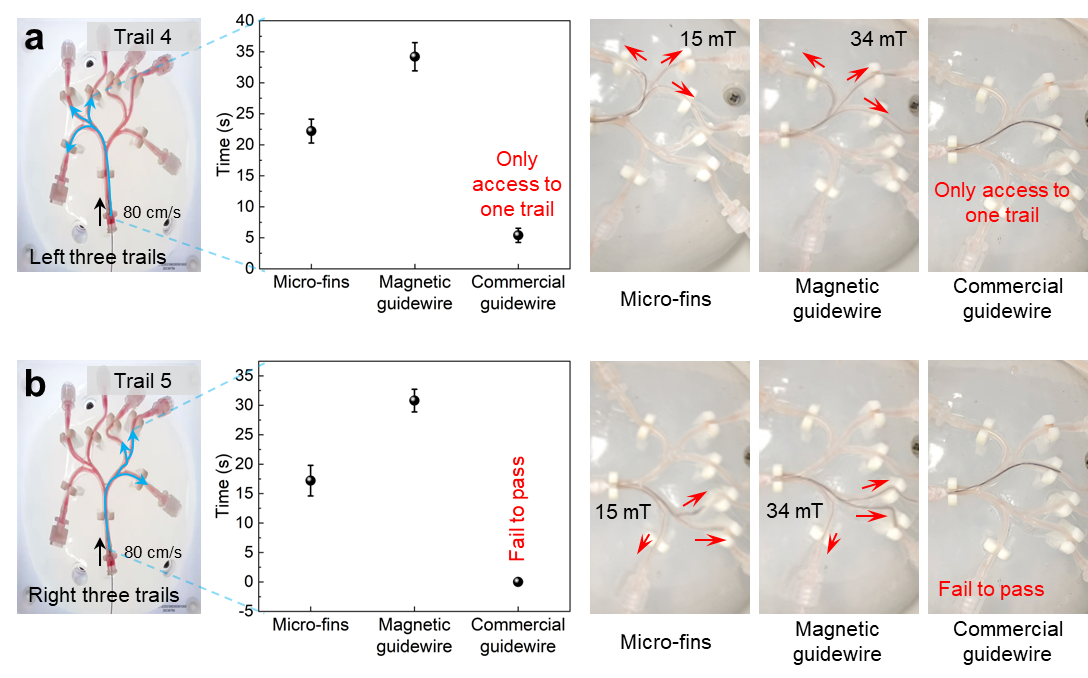


**Fig. S23: Comparison among micro-fins, conventional magnetic guidewire and commercial guidewire on advancing 2 parts of branches of the M4 area.** Data are presented as mean ± standard deviation with *n* = 5.


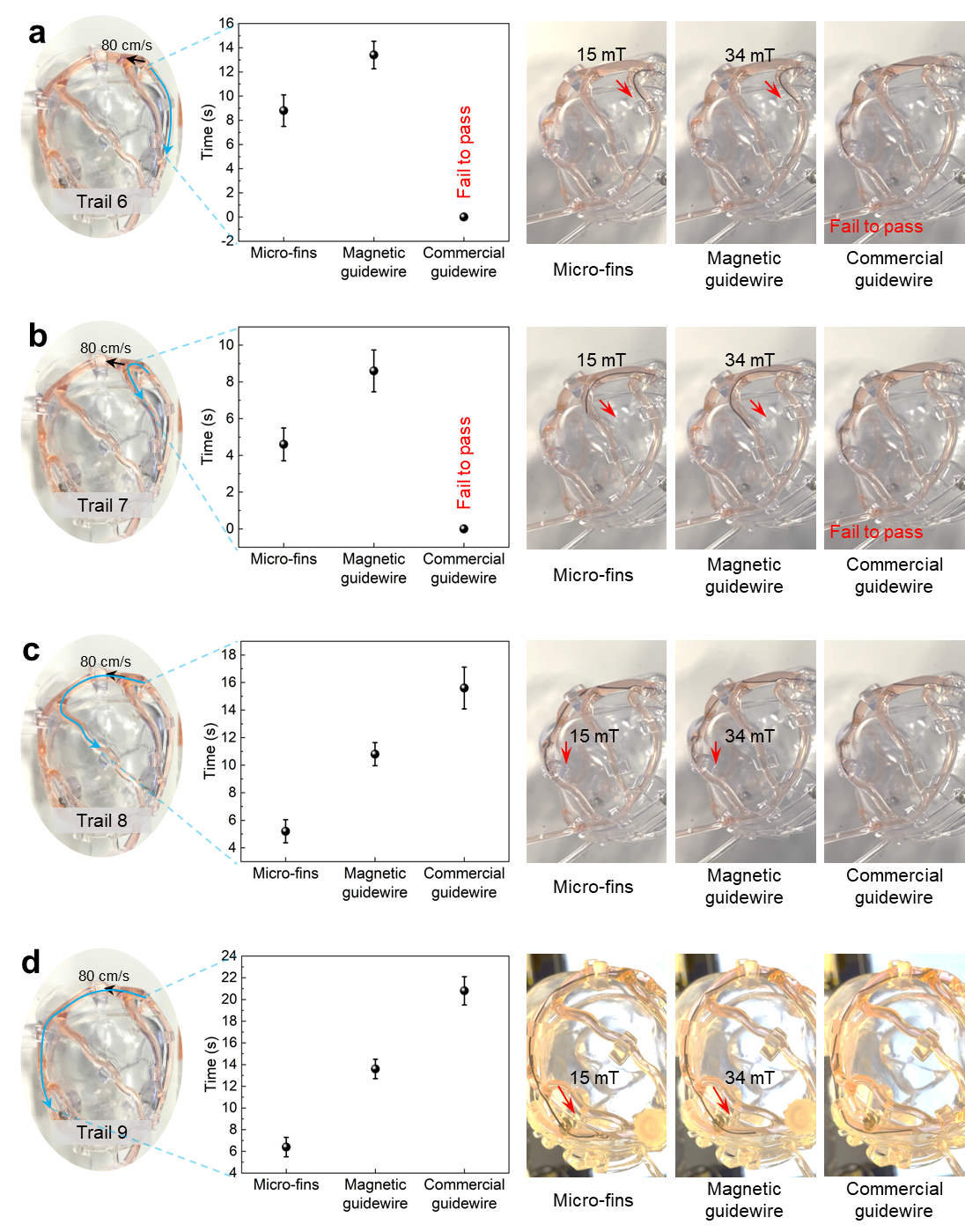


**Fig. S24: Comparison among micro-fins, conventional magnetic guidewire and commercial guidewire on advancing four different target branches of the left part of the cardiovascular phantom.** Data are presented as mean ± standard deviation with *n* = 5.


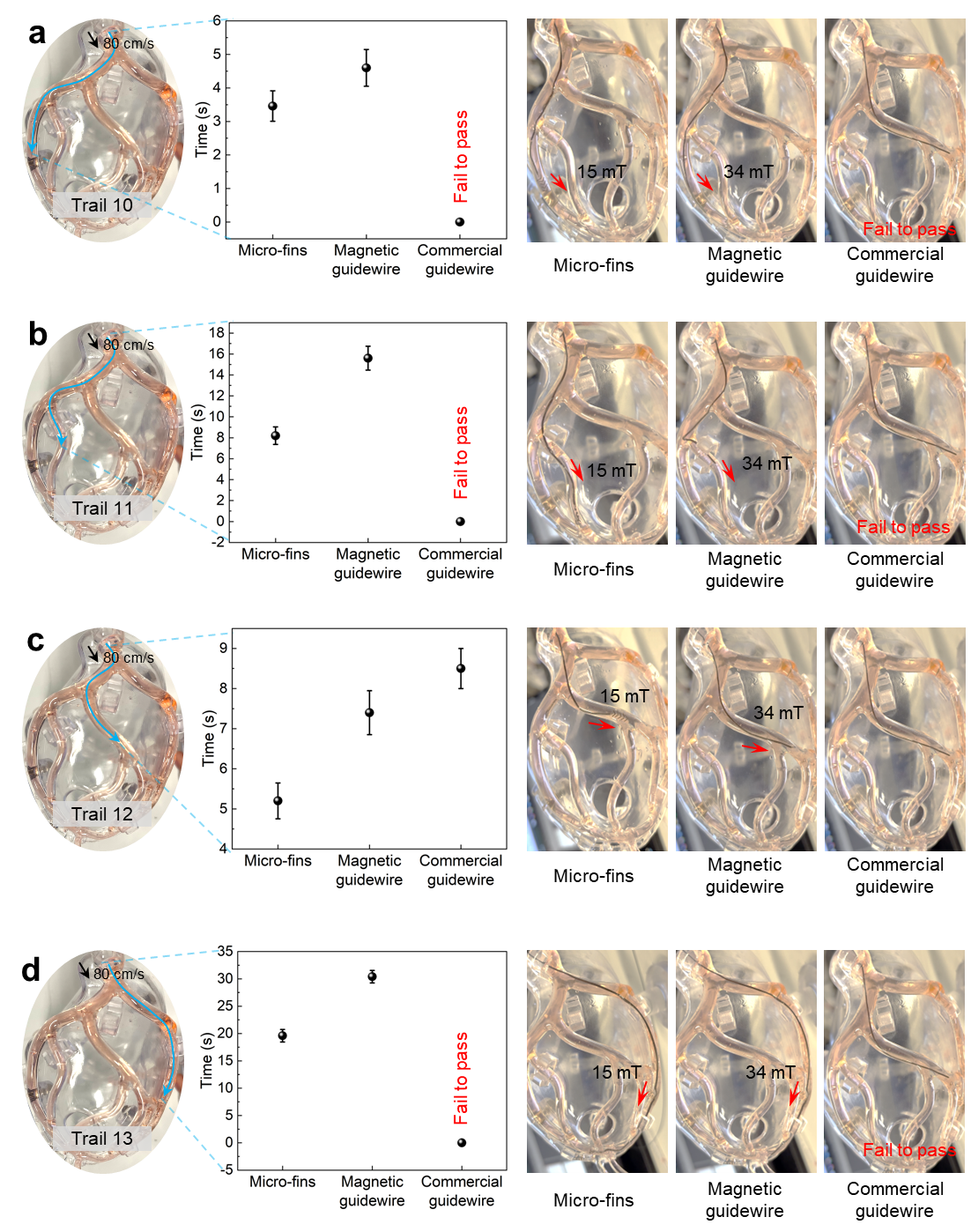


**Fig. S25: Comparison among micro-fins, conventional magnetic guidewire and commercial guidewire on advancing four different target branches of the right part of the cardiovascular phantom.** Data are presented as mean ± standard deviation with *n* = 5.


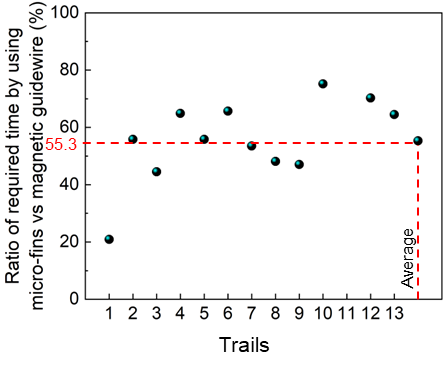


**Fig. S26: Ratio of traversal time by using micro-fins to magnetic guidewire passing all the 13 trails in cerebrovascular and cardiovascular phantoms.**


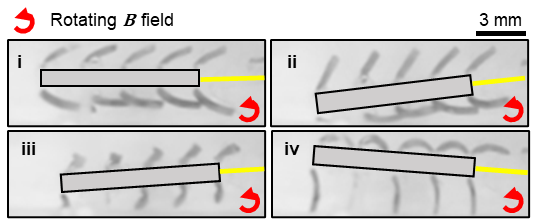


**Fig. S27: Robotic locomotion of micro-fins-integrated tip under a rotating *B* field.**


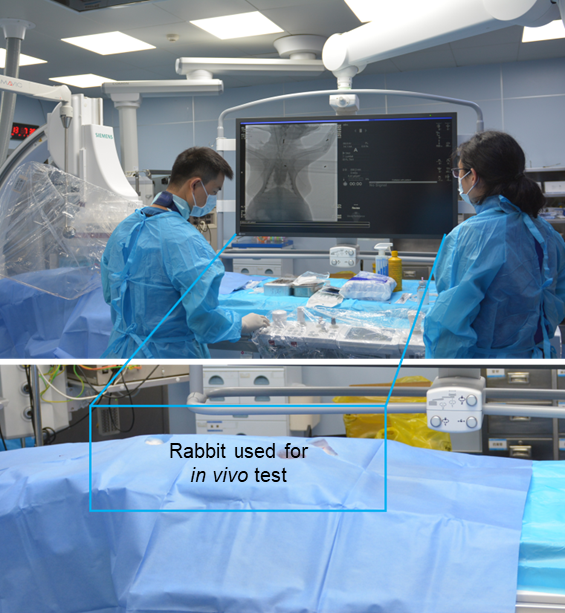


**Fig. S28: *In vivo* experimental set up for a rabbit model.**


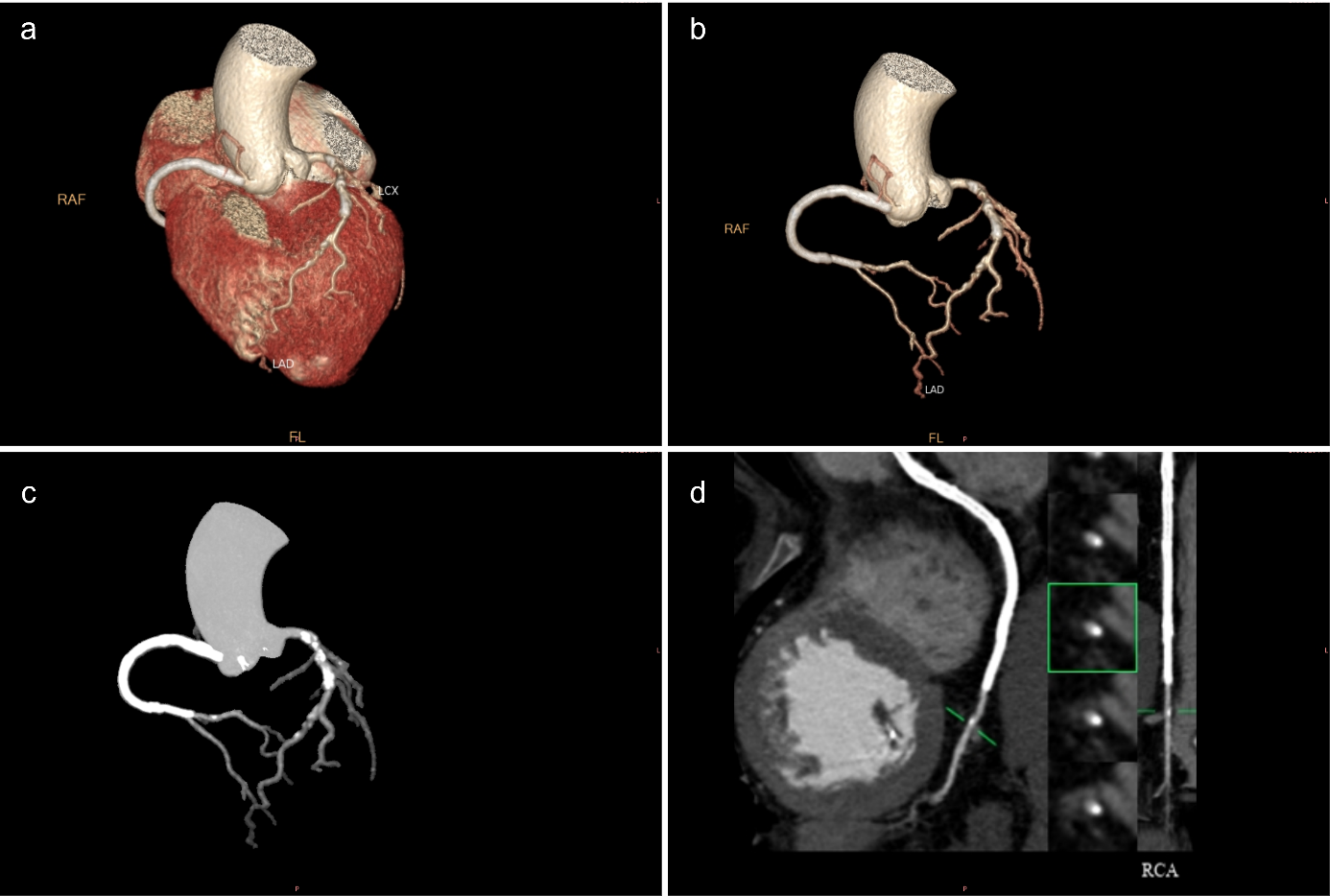


**Fig. S29: The CT scan of the coronary arteries of the human heart showing atherosclerosis.** The white segments indicate atherosclerotic regions, while the grey segments represent normal arteries. To further investigate effective treatments for atherosclerosis, medical research often uses rabbit models to simulate the development of human vascular sclerosis.


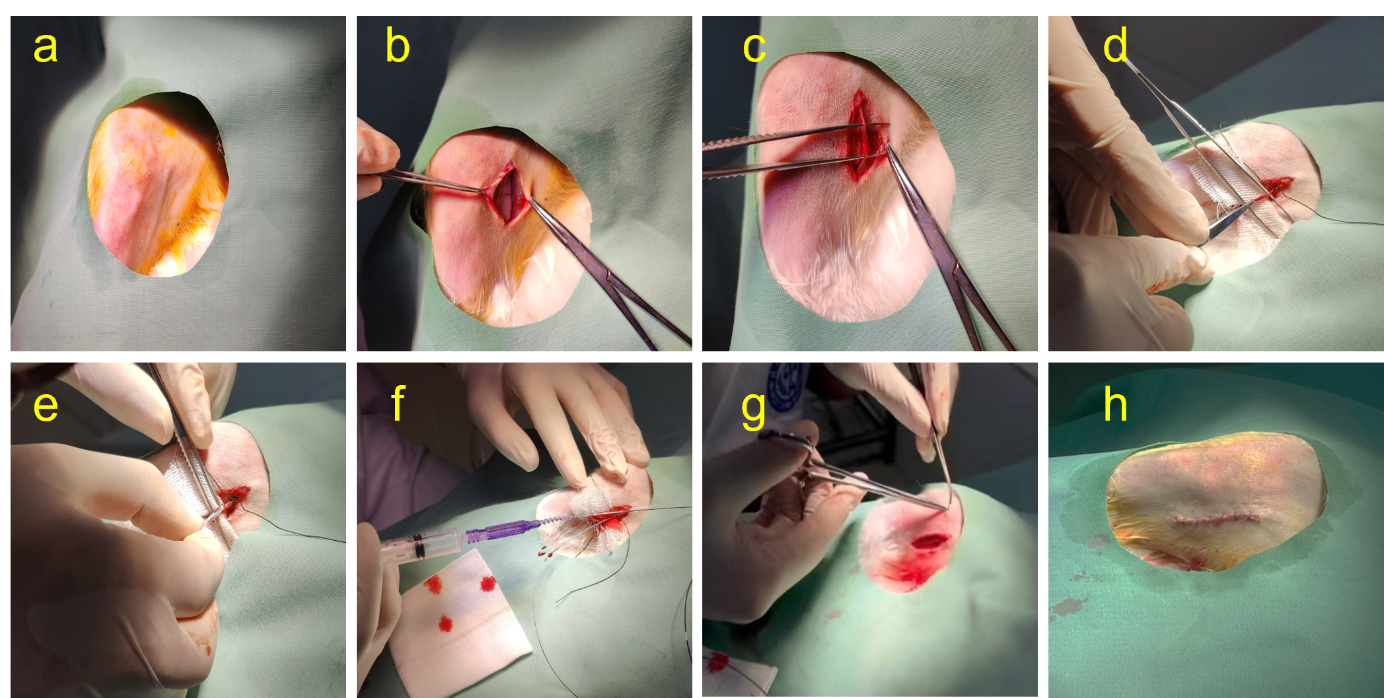


**Fig. S30: Procedure of creating the rabbit model.** (**a**) Swab the surgical site with a suitable disinfectant to clean the skin and remove loose hair. (**b**) Locate the saphenous artery and make a small skin incision of about 1.5 cm using a scalpel. (**c**) Expose a portion of the saphenous artery with small curved forceps without damaging the femoral vein and femoral nerve. (**d**) Lift the saphenous artery using the tied ligature and make a small arteriotomy using micro-scissors. (**e**) Carefully insert the deflated balloon catheter through the incision. (**f**) Advance the balloon to the marked depth (approximately 20 cm), inflate it with air using a 5 mL syringe, and gently withdraw it about 16 cm. Deflate the balloon and repeat this inflation–withdrawal cycle three times to ensure complete endothelial denudation. (**g**) Remove the catheter, immediately tighten the ligature, and close the skin incision using 5-0 suture. (**h**) Disinfect the surgical site with povidone-iodine solution.

**
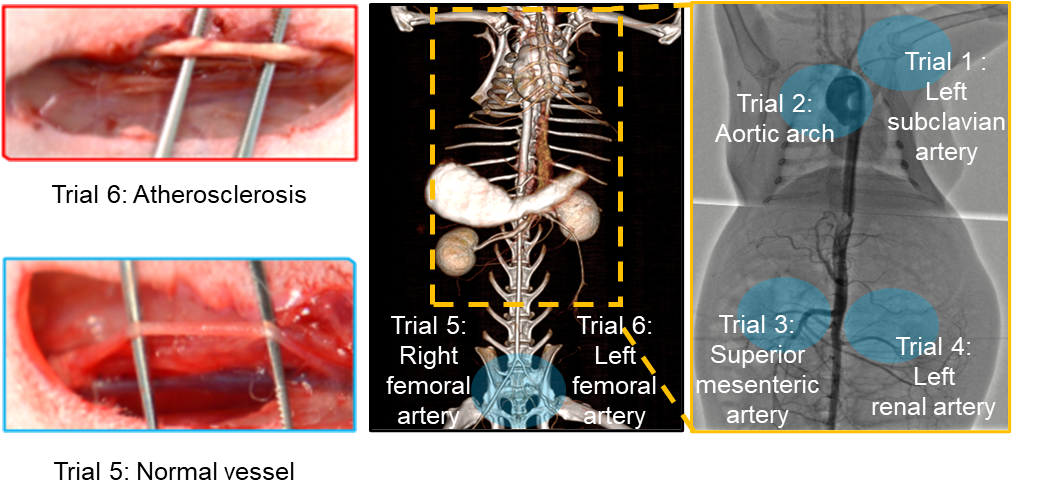
**

**Fig. S31: Reconstructed CTA and DSA images of the blood vessel distribution in a rabbit body.** There are six trials in the following *in vivo* experiments, namely, Trial 1: Left subclavian artery, Trial 2: Aortic arch, Trial 3: Superior mesenteric artery, Trial 4: Left renal artery, Trial 5: Right femoral artery and Trial 6: Left femoral artery.


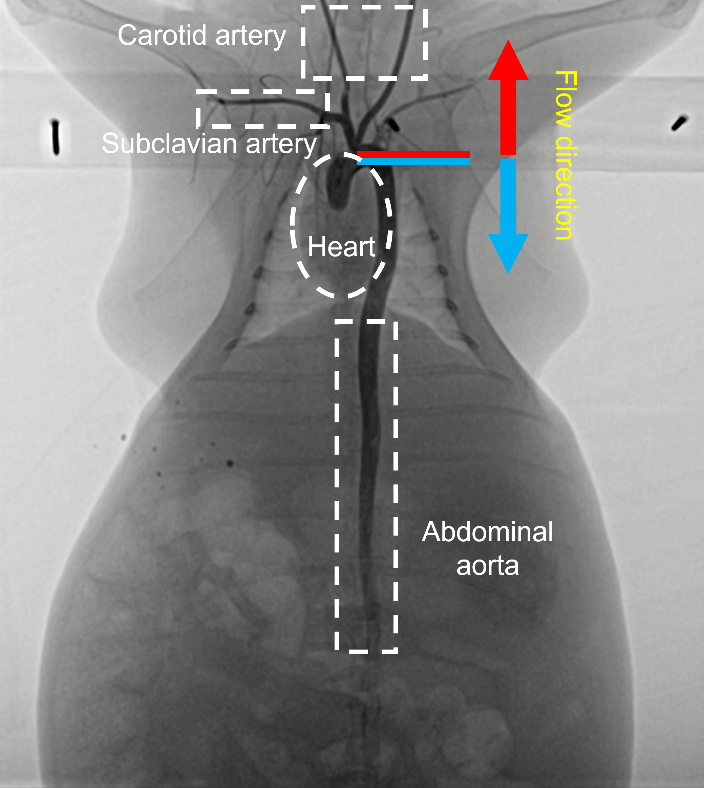


**Fig. S32: DSA image of rabbit vessels.**


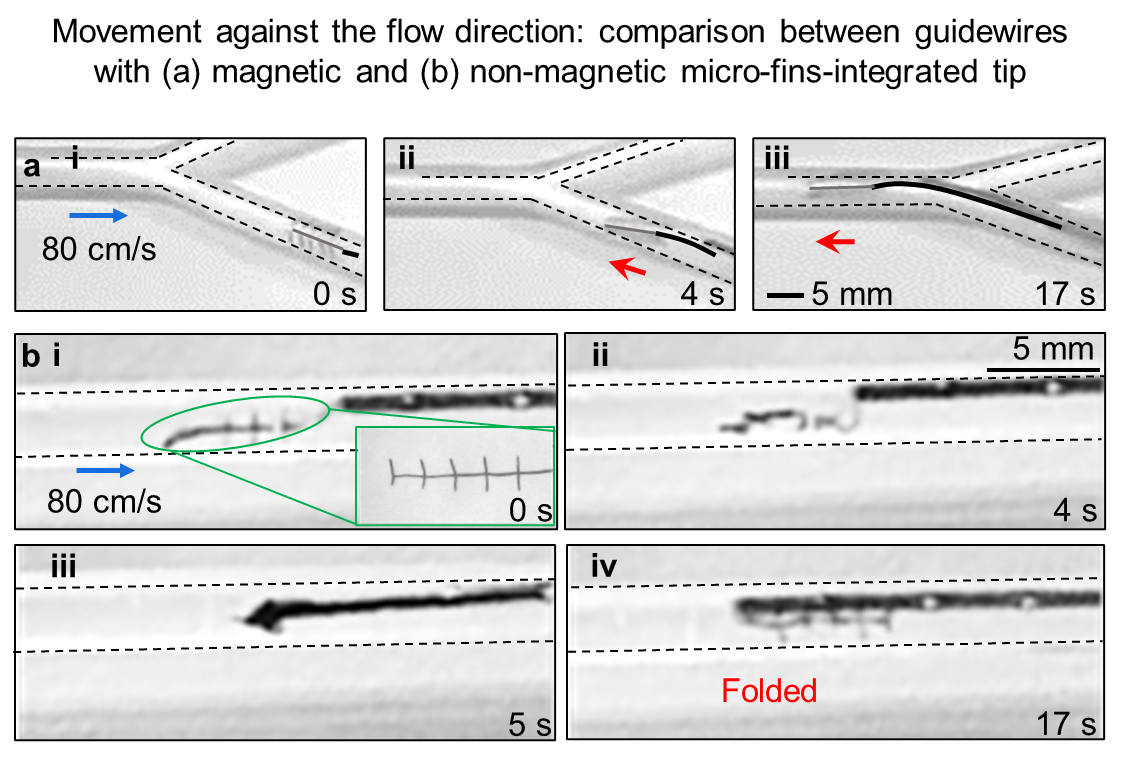


**Fig. S33: Comparison of guidewire movement against the flow direction using (a) current design and (b) non-magnetic micro-fins-integrated tip in our previous work.** The previous design also does not have gradient-stiffness connector.


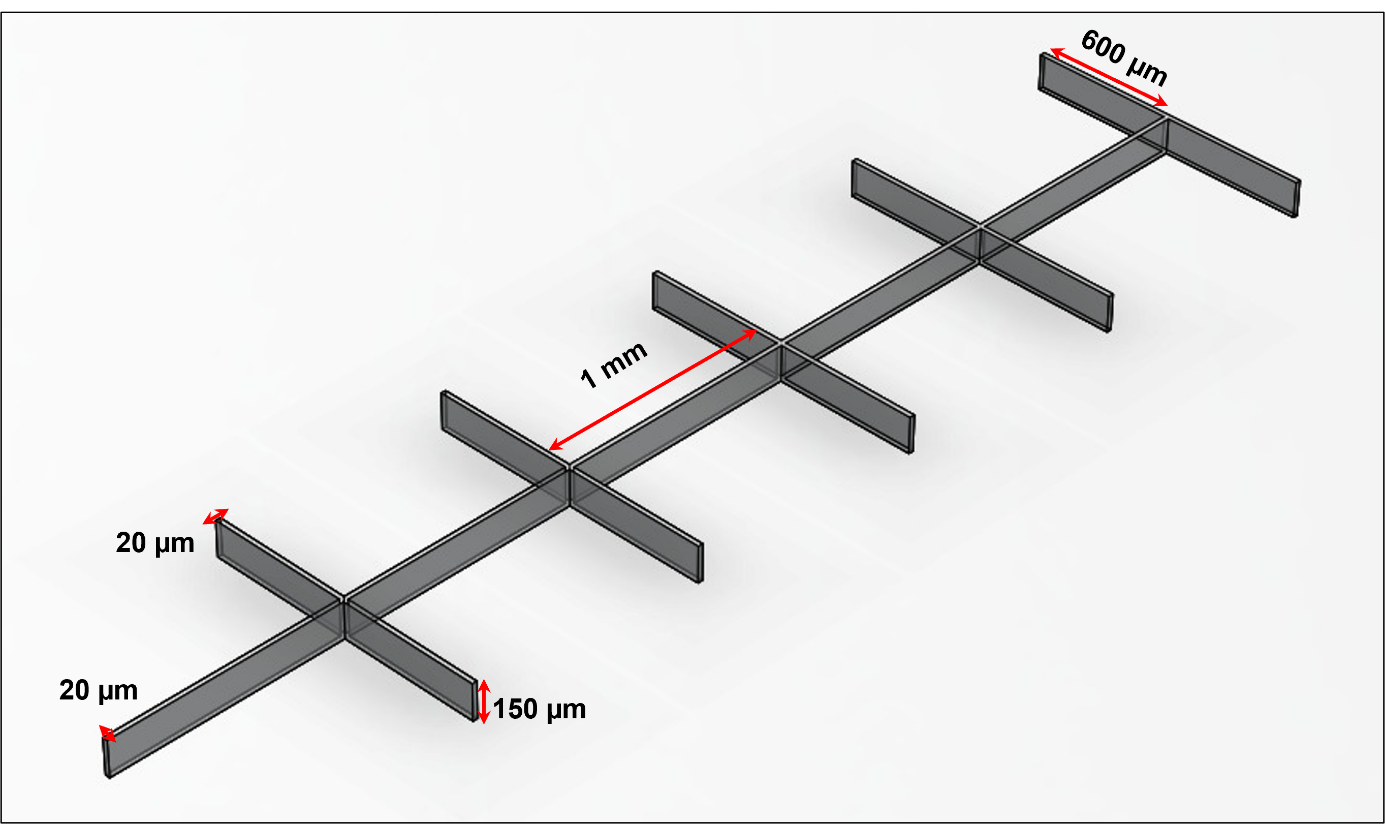


**Fig. S34: Schematic diagram of the non-magnetic micro-fins-integrated tip used in Fig. S25.**


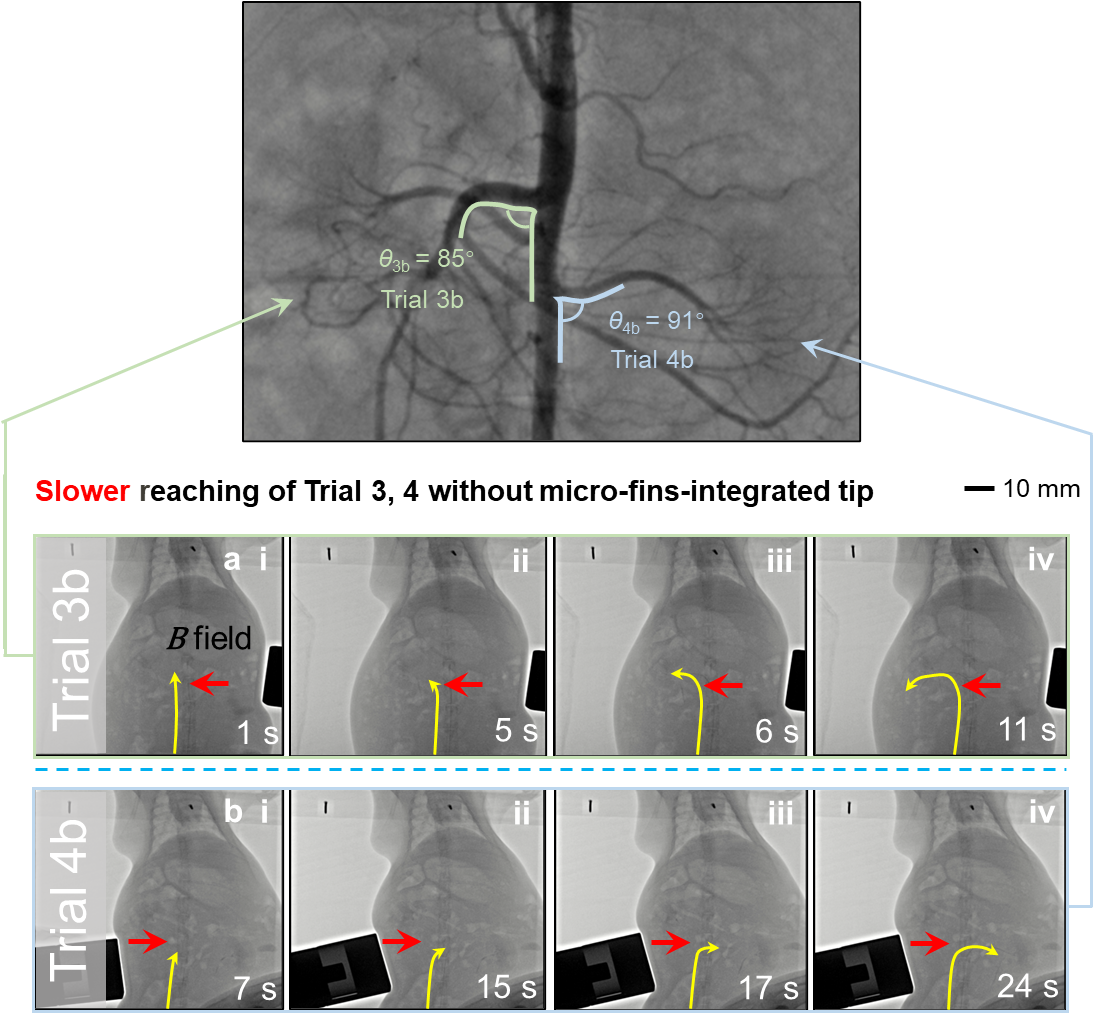


**Fig. S35: The guidewires without micro-fins-integrated tip accessed the superior mesenteric and left renal arteries slower.** Magnetic guidewire without micro-fins-integrated tip going through **(a)** superior mesenteric artery (deflection angle: 85°; total time: 11 s) and **(b)** left renal artery (deflection angle: 91°; total time: 24 s).

**
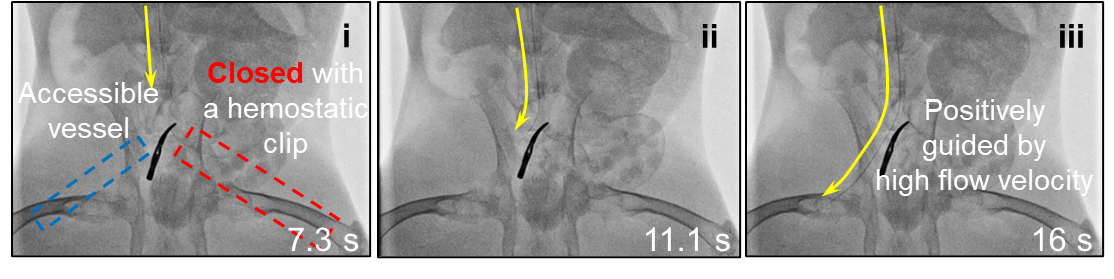
**

**Fig. S36: The micro-fins-integrated tip is guided to the right femoral artery, which exhibits higher flow velocity, when the left artery is occluded by a hemostatic clip.**


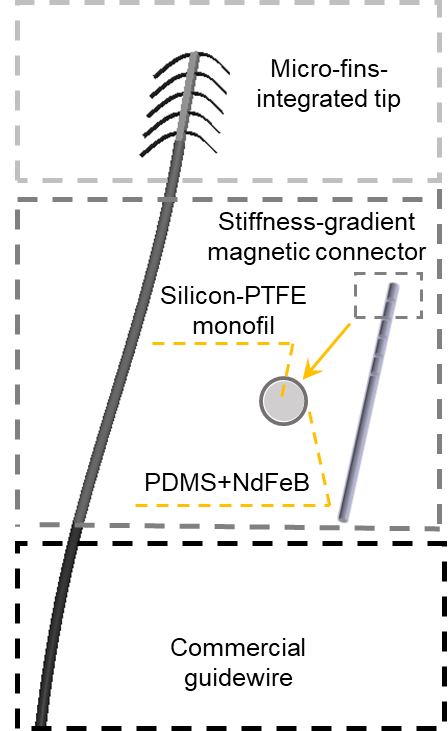


**Fig. S37: Enhanced guidewire with stiffness-gradient magnetic connector and micro-fins-integrated tip.**


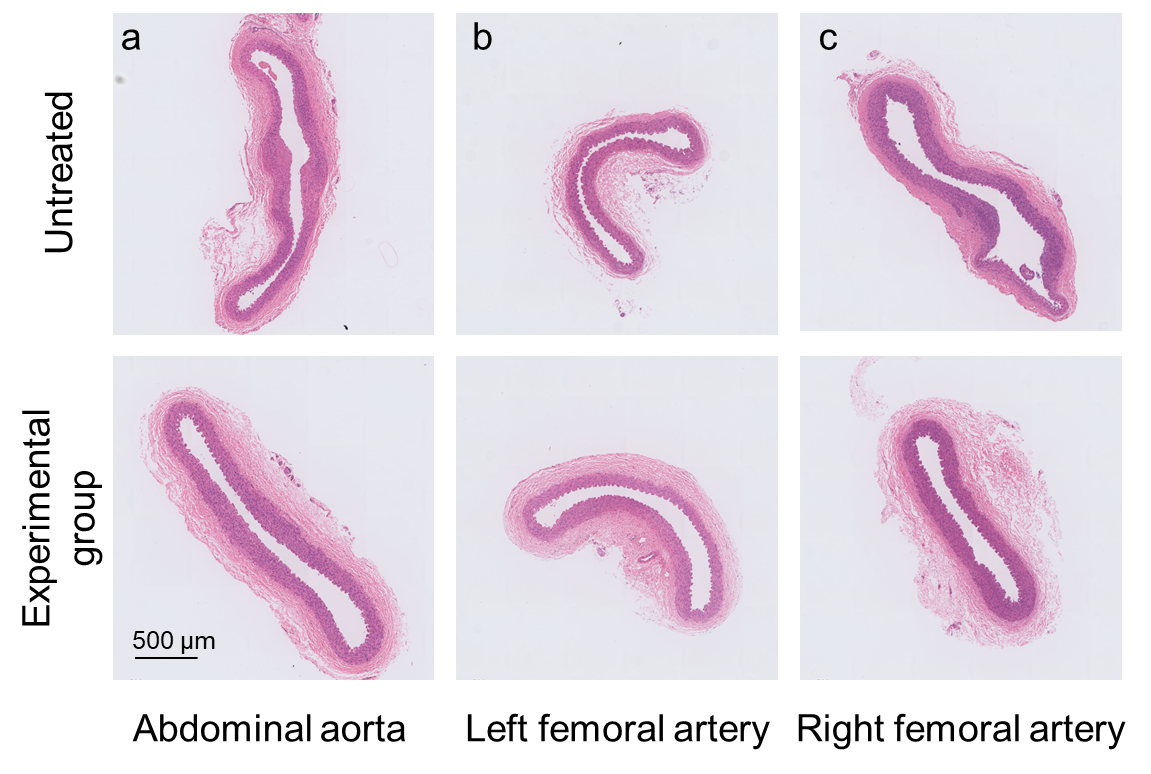


**Fig. S38: Evaluation of vascular injury of (a) abdominal aorta, (b) left femoral artery and (c) right femoral artery after animal experiments.**


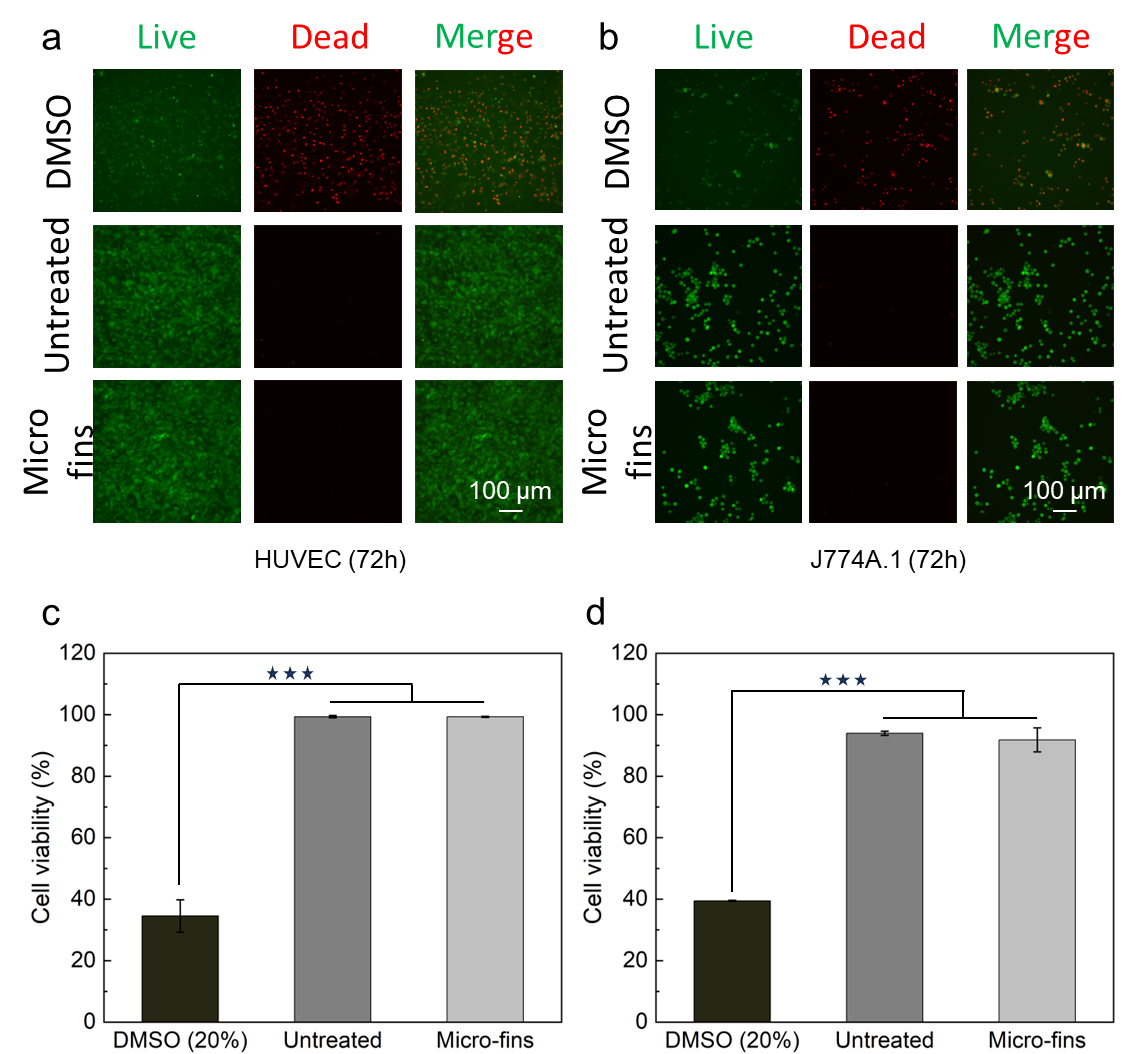


**Fig. S39: Cell viability of Parylene-C-coated micro-fins.** Fluorescence micrographs of (a) HUVECs (ATCC) and (b) J774A.1 murine monocyte–macrophages (ATCC) after 72 h incubation with the coated sample or with 20% DMSO (Sigma-Aldrich). Sample dimensions: 1.5 mm × 1.5 mm × 10 mm.
 Viability of (c) HUVEC and (d) J774A.1, which are quantified using the Live/Dead Cell Staining Kit (R37601, Thermo Fisher Scientific). Negative control: untreated cells (no material); positive control: 20% DMSO. Viability was normalized to 100% for the negative control and 0% for the positive control. Fluorescence images were acquired on a Leica DMi8 microscope. Data are presented as mean ± standard deviation with *n* = 3.


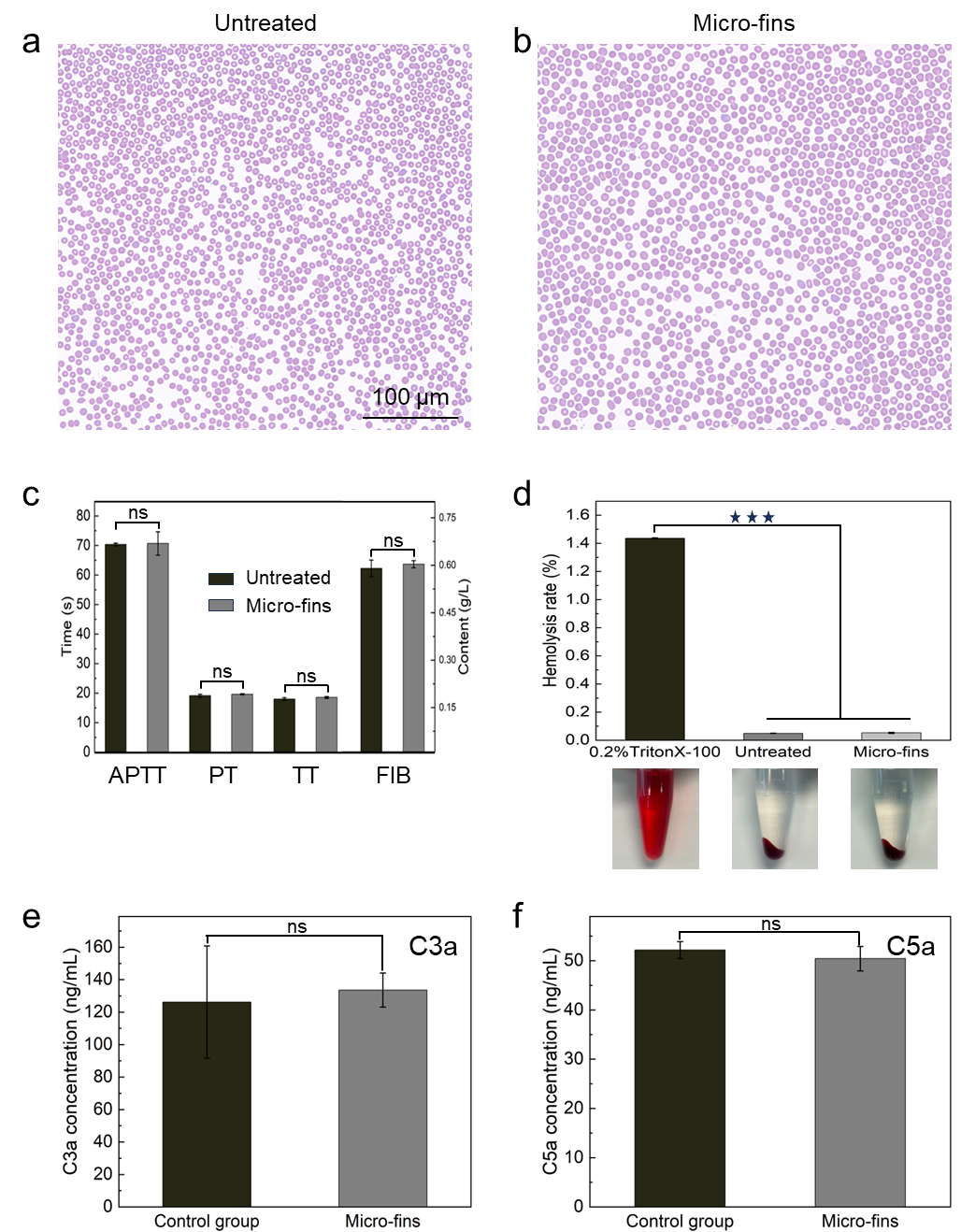


**Fig. S40: Hemocompatibility of Parylene-C-coated micro-fins.** Representative hematoxylin–eosin–stained blood smears showing cell morphology, (a) untreated control and (b) after exposure to the micro-fins. (c) Thrombogenicity assessment by four indices—activated partial thromboplastin time (APTT), prothrombin time (PT), thrombin time (TT), and fibrinogen level (FIB). Whole blood containing 0.2% (v/v) Triton X-100 served as the positive control (defined as 100%), and blood incubated with a Parylene-C–coated glass slide served as the negative control (0%). Blood was diluted 1:3 prior to testing. (d) Hemolysis assay: samples (1.5 mm × 1.5 mm × 10 mm) were placed in 1.5 mL EP tubes with rabbit whole blood and incubated at 37 °C on a horizontal shaker (60 rpm). The negative control contained no sample, and the positive control contained 0.2% (v/v) Triton X-100. Data are presented as mean ± standard deviation with *n* = 3. Immune activation evaluation via complement components **(e)** C3a and **(f)** C5a. Data are presented as mean ± standard deviation with *n* = 9.


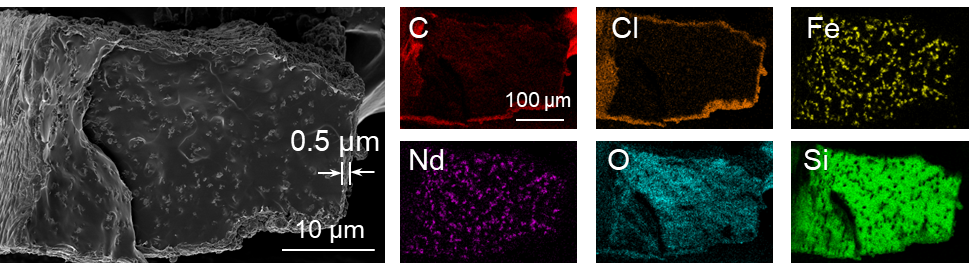


**Fig. S41: SEM image of the micro-fins under Parylene-C and corresponding EDS characterization.**


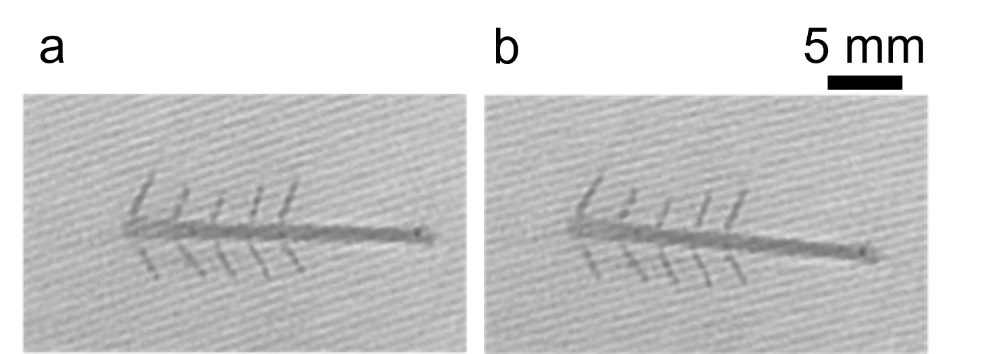


**Fig. S42: DSA image of two micro-fins-integrated tip with contrast agent.**

**Table S2 Comparisons between the proposed magnetic guidewire in this work and reported work in terms of *B* field source, *B* field strength and *B* field alignment.**

| Ref | ***B*** field source | Required ***B*** filed strength | ***B*** field misalignment tolerance/Control precision | Traversal time |
| --- | --- | --- | --- | --- |
| Hwang J et al. 2025 ^16^ | Uniform ***B*** fields by Helmholtz coils | 10 – 40 mT | N.A. | N.A. |
| Dreyfus R et al. 2024 ^17^ | Uniform ***B*** fields by Helmholtz coils | 150 mT | N.A. | N.A. |
| Kim Y et al. 2019 ^18^ | Uniform ***B*** fields by Helmholtz coils | 80 mT | N.A. | N.A. |
| Mao L et al. 2024 ^19^ | Uniform ***B*** fields by Helmholtz coils | 43 mT | N.A. | N.A. |
| Pancaldi L et al. 2020 ^20^ | Uniform ***B*** fields by Helmholtz coils | 8 mT | N.A. | N.A. |
| Yan Y et al. 2023 ^15^ | Gradient ***B*** fields by magnets | 50 mT | N.A. | N.A. |
| Ge T et al. 2023 ^21^ | Gradient ***B*** fields by magnets | 250 mT | N.A. | N.A. |
| Li N et al. 2023 ^22^ | Uniform ***B*** fields by eight-coil electromagnetic system | 120 mT | N.A. | N.A. |
| Kim Y et al. 2022 ^23^ | Gradient ***B*** fields by magnets | 80 mT | Develop strategies on precise control of ***B*** field | N.A. |
| Tiryaki M et al. 2023 ^24^ | Ultrahigh ***B*** fields up to 7-Tesla by preclinical MRI scanner | 7 T | Pose accuracy tunable from 0.47 mm / 2.14° down to 40 µm / 0.15° | N.A. |
| Gharamaleki N et al. 2025 ^25^ | Uniform ***B*** fields by electromagnetic actuation system | 22 mT | Develop strategies on precise control of ***B*** field | N.A. |
| Lu K et al. 2024 ^26^ | Uniform ***B*** fields + Gradient ***B*** fields | 10 mT | Develop strategies on precise control of ***B*** field | N.A. |
| Diller E et al. ^27^ | Uniform ***B*** fields by Helmholtz coils | 8.3 mT | Develop strategies on precise control of ***B*** field | N.A. |
| Li et al. ^28^ | Gradient ***B*** field | N.A. | Max angular control error < 3° (after system calibration) | N.A. |
| Zhang et al. ^29^ | Rotating ***B*** field | N.A. | Programmable RMF tilt γ ∈ [0, π] (0–180°); stability requires maintaining commanded tilt (device operates in 2–8 mT RMF window) | N.A. |
| Erin et al. ^30^ | Uniform ***B*** field + strong programmable ***B*** field gradients | Gradient 3834 mT·m⁻¹ | Force 1.673 N; σ(force) < 0.006 N, uncertainty < 0.02 N (high repeatability of commanded output) | N.A. |
| Peng et al. ^31^ | Uniform ***B*** field by EMA coils | 6 mT | N.A. | ~2 min / 100 cm, femoral access |
| Ligtenberg et al. ^32^ | Gradient ***B*** field | 5 mT | N.A. | Inside renal artery ~ 115 s; reverse swim ~ 125 s; representative trials show renal entry < 60 s |
| Zielasek et al. ^33^ | Uniform ***B*** field by electromagnetic coils | 5-25 mT | N.A. | Median procedure time 105 s (magnetically guided wire) vs 181 s (conventional) in 3D silicone aorta model |
| Wang et al. ^34^ | Uniform ***B*** field | 65 mT | N.A. | ~15–30 min full therapeutic workflow |
| Pancaldi et al. 2025^35^ | Three orthogonal coils re-orienting an internal permanent magnet | 5-6 mT | Catheter tip field-orientation control with a mean angular error of ~1.7° for 0–90° steps and a ~0.36 s rise time for a 90° step. | Completing selective branch entry took ~58 ± 8 s (from vertebral to muscular branches, n = 3) and ~72 ± 15 s (infraorbital artery branches, n = 4).. |
| Yang et al. 2025^36^ | Uniform ***B*** fields + Gradient ***B*** fields | 12-177 mT | N.A. | N.A. |
| This work | Gradient ***B*** fields by magnets | 0 mT  (To branches with higher flow velocity)  Below 15 mT  (For other vascular branches, turning and unbuckling soft guidewire) | Allowed misalignment angle 0° ~ 45° | Allowed full access to major arteries of rabbit model within one minute |

**Table S3 Parameters for designing micro-fins-integrated tip for flow-assisted multi-modal vascular intervention.**

|  | | *d* = 0.5 mm | *d* = 1.0 mm | *d* = 1.5 mm |  | Other |
| --- | --- | --- | --- | --- | --- | --- |
| *θ* = 30 ° | | Sample 1 | Sample 2 | Sample 3 |  | *w*_1_ = 0.3 mm |
| *θ* = 45 ° | | Sample 4 | Sample 5 | Sample 6 |  | *w*_2_ = 0.2 mm |
| *θ* = 60 ° | | Sample 7 | Sample 8 | Sample 9 |  | *t* = 0.15 mm |
|  | *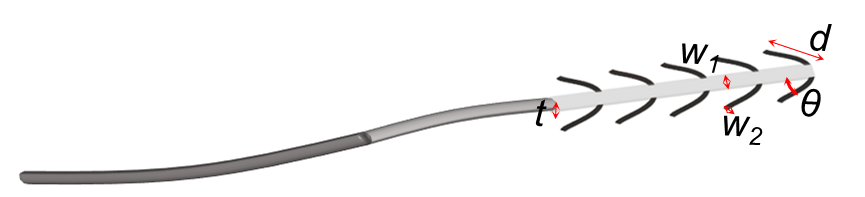*  The *θ* refers to the original fabrication angle rather than the variation under ***B*** field (*θ_d_*, *θ* dynamic) | | | | | |

**Table S4 Comparisons of Young’s Modulus and Stiffness between Silicon-PTFE monofil and Guidewire in different diameters. The lengths of all the testing specimens are 20 mm.**

|  | Young’s Modulus (GPa) | Density (g/cm^3^) | Stiffness (N/m) | Poisson's ratio |
| --- | --- | --- | --- | --- |
| PDMS | 2.6×10-3 | 0.965~0.97 | / | 0.48~0.5 |
| Silicon-PTFE monofil (*Φ* = 100 μm) | 25.6 | 9.5×10-3 | 0.38 | 0.47 |
| Silicon-PTFE monofil (*Φ* = 200 μm) | 3.1 | 1.27×10-2 | 0.73 | 0.47 |
| Silicon-PTFE monofil (*Φ* = 300 μm) | 2.7 | 1.51×10-2 | 3.19 | 0.47 |
| Guidewire (*Φ* = 460 μm) | 0.3 | 4.63×10-2 | 2.03 | 0.3~0.33 |
| Guidewire (*Φ* = 640 μm) | 0.7 | 3.1×10-2 | 4.40 | 0.3~0.33 |

**Table S5 Comparisons between the proposed magnetic guidewire in this work and previous work.**

| Reference | Young's modulus | Allowed misalignment angle | Unfastening buckled guidewire | Steerability by the flow | Advancing against flow direction |
| --- | --- | --- | --- | --- | --- |
| Kim Y et al. 2019 ^18^ | 14 MPa (stiffer segment)  1.4 MPa (softer segment) | N.A. (Uniform magnetic fields by Helmholtz coils) | No | No | N.A. |
| Kim Y et al. 2022 ^23^ | N.A. | N.A. (Using Magnet) | No | No | N.A. |
| Dreyfus R et al. 2024 ^17^ | N.A.  (Gold-coated NdFeB encapsulated in a 50-μm 35D Pebax jacket.) | N.A. (Eight-coiled electromagnetic navigation system) | No | No | N.A. |
| Pancaldi L et al. 2020 ^20^ | 10 MPa (PDMS)  4 GPa (Kapton)  3 GPa (Filament) | N.A. (Uniform magnetic fields by Helmholtz coils) | No | Yes | N.A. |
| Yan Y et al. 2023 ^15^ | ADS: 3.92 ± 0.30 MPa  FDS: 0.50 ± 0.08 MPa | N.A. (Magnet) | No | Yes | N.A. |
| Our work | 2.6 MPa  (Pure PDMS) | 0 ~ 45° (Magnet) | Yes | Yes | Yes (when docked on the commercial guidewire) |

**Table S6 Comparisons of a rabbit model with atherosclerosis in the left femoral artery and human heart's coronary arteries with atherosclerotic.**

|  | Similarities between rabbit femoral artery atherosclerosis model and human cardiovascular atherosclerosis |
| --- | --- |
| **Pathological Features** |  |
| Lipid Deposition | In both models, lipid accumulation occurs within the arterial wall, forming lipid plaques, which are early features of atherosclerosis. |
| Fibrous Cap Formation | As the lesion progresses, a fibrous cap composed of smooth muscle cells and collagen fibers forms over the lipid core. |
| Inflammatory Response | Infiltration of monocytes, macrophages, and lymphocytes, along with the release of inflammatory mediators, can be observed at the atherosclerotic lesion site in both models. |
| **Pathogenesis** |  |
| Hypercholesterolemia | High cholesterol levels, especially elevated low-density lipoprotein cholesterol (LDL-C), are key risk factors for atherosclerosis, observed in both rabbit models and humans. |
| Oxidative Stress | The deposition and effects of oxidized LDL in the arterial wall are crucial mechanisms of atherosclerosis in both models. |
| Endothelial Dysfunction | Damage or abnormal function of endothelial cells increases the risk of atherosclerosis, a common feature in both rabbits and humans. |
| **Progression of Lesions** |  |
| Plaque Rupture | In advanced stages, atherosclerotic plaques may rupture, leading to thrombosis and acute cardiovascular events. Similar phenomena can be observed in both rabbits and humans. |
| Vascular Calcification | As the disease progresses, calcification within the plaques occurs, marking a late stage of atherosclerosis in both models. |

**Table S7 Variables’ explanation used in Supplementary Note S4 and Note S8.**

| **Variables** | **Explanation** |
| --- | --- |
| *ρ* (kg/m^3^) | Fluid density |
| *U* (m/s) | Flow field velocity |
| *C*_D_ | Drag Coefficient |
| *Re* | Reynolds Number |
| *t* (mm) | Thickness of micro-fins-integrated tip |
| *w*_1_ (mm) | Width of micro-fins-integrated tip’s main body |
| *d* (mm) | Length of micro-fins-integrated tip’s branch |
| *θ* (°) | Angle between micro-fins-integrated tip’s branch and main body |
| 2*n* | Number of micro-fins-integrated tip branches |
| *A*_main_ (mm^2^) | Projected Area of micro-fins-integrated tip’s main body |
| *A*_Fin_ (mm^2^) | Projected Area of micro-fins |
| *F*_main_ | Thrust of micro-fins-integrated tip main body |
| *F*_Fin_ | Total thrust of micro-fins |
| *F*_total_ | Thrust of whole micro-fins-integrated tip |
| 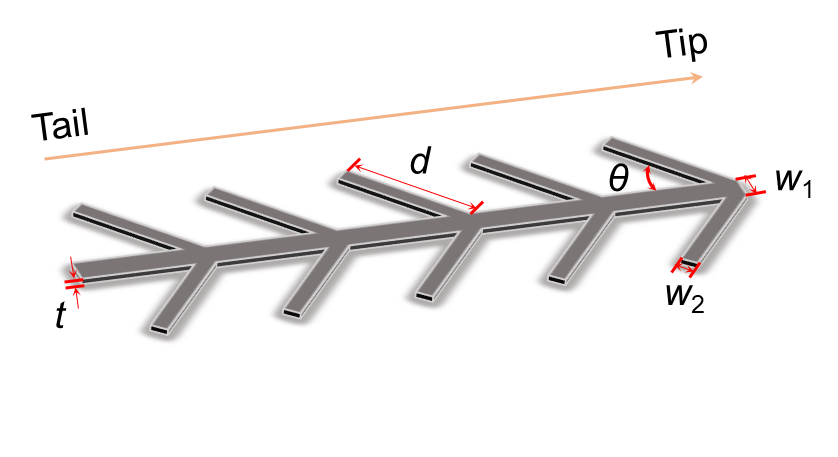 | |
|  |  |
| *x* (mm) | Length from fixed end of cantilever beam |
| *y*(x) (mm) | Cantilever beam deflection at *x* |
| *M*(x) (N/m) | Bending moment of cantilever beam at *x* |
| *E* (GPa) | Young’s Modulus of cantilever beam |
| *I* (mm^4^) | Moment of inertia of cantilever beam section |
| *A* (mm^2^) | Area element |
| *r* (mm) | Radios of cantilever beam section |
| *L* (mm) | Length of cantilever beam |
| *P* (N) | External force exerted on the free end of the cantilever beam |
| *S* (N/m) | Stiffness of cantilever beam |
| 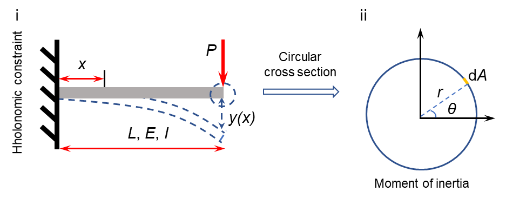 | |

**Table S8 Design of 3D printed phantoms.**

| **Phantom label** | **Design** | **Important parameters** |
| --- | --- | --- |
| **Phantom 1:**  **Y-shaped set up for testing drag force** | **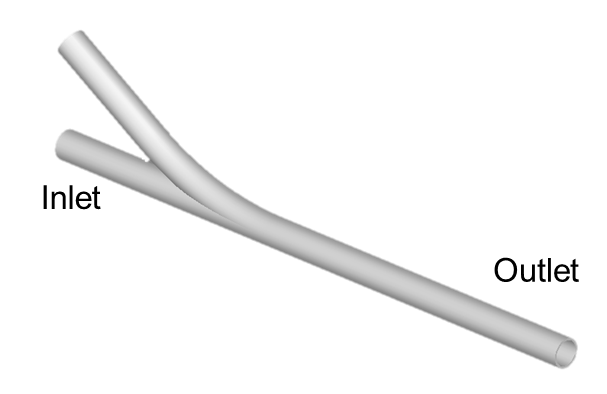** | *Φ*_Intlet_ = 4.8 mm  *Φ*_Outlet_ = 4.8 mm |
|  |  |  |
|  |  |  |
| **Phantom 2:**  **Straight tube** | **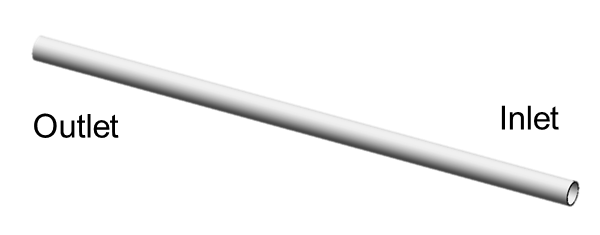** | *Φ*_Intlet_ = 4.8 mm  *Φ*_Outlet_ = 4.8 mm |
|  |  |  |
|  |  |  |
| **Phantom 3:**  **Tapered Y-shaped tube** | **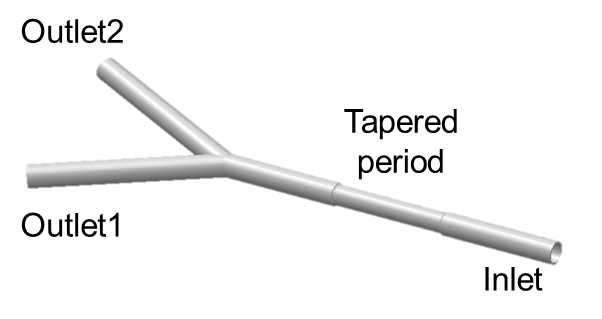** | *Φ*_Intlet_ = 4.8 mm  *Φ*_Tapered peirod_ = 2.4 mm  *Φ*_Outlet1_ = 4.8 mm  *Φ*_Outlet2_ = 3 mm |
|  |  |  |
|  |  |  |
| **Phantom 4:**  **Y-shaped tube with small diameter** | **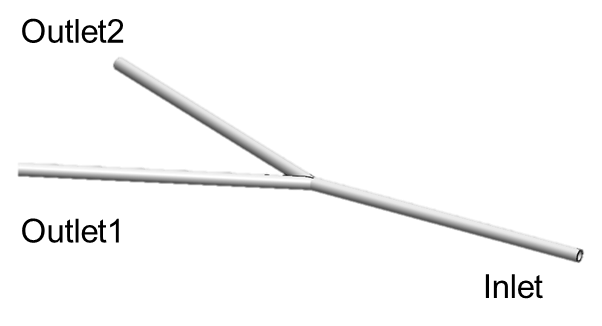** | *Φ*_Intlet_ = 2.4 mm  *Φ*_Outlet1_ = 2 mm  *Φ*_Outlet2_ = 1.5 mm |
|  |  |  |
| **Phantom 5:**  **Y-shaped tube with large diameter** | **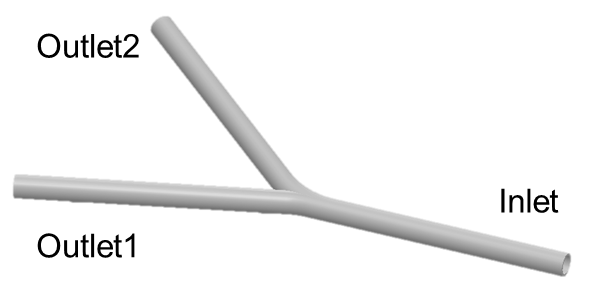** | *Φ*_Intlet_ = 4.8 mm  *Φ*_Outlet1_ = 4 mm  *Φ*_Outlet2_ = 3 mm |
|  |  |  |
|  |  |  |
| **Phantom 6:**  **Curved tubes with different curvatures** | **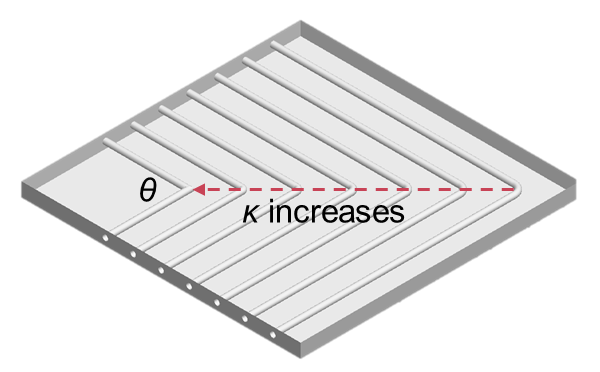** | *θ* **=** 90°  Φ **=** 2 mm  *κ* **=** 0.33 mm^-1^ to 1.3 mm^-1^ |
|  |  |  |
|  |  |  |
| **Phantom 7:**  **Straight tube** | **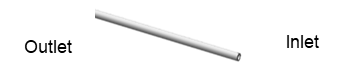** | *Φ*_Intlet_ = 2.4 mm  *Φ*_Outlet_ = 2.4 mm |

**Movie S1:** Enhanced passability under lower magnetic field strength using the micro-fins-integrated tip.

**Movie S2:** Real-time manipulation of the deflection angle of the micro-fins-integrated tip.

**Movie S3**: Locomotion for passing M4 segment of a cerebral vascular model.

**Movie S4:** Going across a tapered tube.

**Movie S5:** Unbuckle the soft guidewire using the micro-fins-integrated tip.

**Movie S6:** *In vivo* manipulation of the magnetic guidewire with micro-fins-integrated tip in a rabbit model.

**Movie S7:** Comparison between the proposed guidewire and commercial guidewire reaching subclavian artery.

**Movie S8:** Comparison between the proposed guidewire and commercial guidewire passing through aortic arch.

**Movie S9:** Comparison of magnetic guidewire with and without micro-fins-integrated tip passing superior mesenteric and renal artery.

# References

1 Chen, X., Cao, H., Li, Y., Chen, F., Peng, Y., Zheng, T. & Chen, M. Hemodynamic influence of mild stenosis morphology in different coronary arteries: a computational fluid dynamic modelling study. Frontiers in Bioengineering and Biotechnology. 12, 1439846 (2024).

2 De Nisco, G., Rizzini, M. L., Verardi, R., Chiastra, C., Candreva, A., De Ferrari, G., D'Ascenzo, F., Gallo, D. & Morbiducci, U. Modelling blood flow in coronary arteries: newtonian or shear-thinning non-newtonian rheology? Computer Methods and Programs in Biomedicine. 242, 107823 (2023).

3 Zhang, C. & Moore, I. D. Nonlinear mechanical response of high density polyethylene. Part II: Uniaxial constitutive modeling. Polymer Engineering & Science 37, 414-420 (1997).

4 Nikolov, S. & Doghri, I. A micro/macro constitutive model for the small-deformation behavior of polyethylene. Polymer 41, 1883-1891 (2000).

5 Abdul-Hameed, H., Messager, T., Ayoub, G., Zaïri , F., Abdelaziz, M. N., Qu, Z. & Zaïri, F. A two-phase hyperelastic-viscoplastic constitutive model for semi-crystalline polymers: application to polyethylene materials with a variable range of crystal fractions. Journal of the Mechanical Behavior of Biomedical Materials 37, 323-332 (2014).

6 Boyce, M. C. & Arruda, E. M. Constitutive models of rubber elasticity: a review. Rubber Chemistry and Technology 73, 504-523 (2000).

7 Diao, Y., Yang, J., Zhang, Y., Zhang, D. & Du, Y. Solving multi-material problems in solid mechanics using physics-informed neural networks based on domain decomposition technology. Computer Methods in Applied Mechanics and Engineering 413, 116120 (2023).

8 Yang, J., Liu, X., Diao, Y., Chen, X. & Hu, H. Adaptive task decomposition physics-informed neural networks. Computer Methods in Applied Mechanics and Engineering 418, 116561 (2024).

9 Fitzgerald, E. R. & Watson, M. Dynamic mechanical properties of polyethylene from 25° to150°C. The Journal of the Acoustical Society of America 32, 584-593 (1960).

10 Khanna, Y. P., Turi, E. A., Taylor, T. J., Vickroy, V. V. & Abbott, R. F. Dynamic mechanical relaxations in polyethylene. Macromolecules 18, 1302-1309 (1985).

11 Xing, J., Price, W. & Chen, Y. A mixed finite–element finite–difference method for nonlinear fluid–structure interaction dynamics. Proceedings of the Royal Society of London. Series A: Mathematical, Physical and Engineering Sciences 459, 2399-2430 (2003).

12 Malvè, M., García, A., Ohayon, J. & Martínez, M. A. Unsteady blood flow and mass transfer of a human left coronary artery bifurcation: FSI vs. CFD. International Communications in Heat and Mass Transfer 39, 745-751 (2012).

13 Dill, E. H. Kirchhoff's theory of rods. Archive for History of Exact Sciences, 1-23 (1992).

14 Langer, J. & Singer, D. A. Lagrangian aspects of the kirchhoff elastic rod. SIAM Review 38, 605-618 (1996).

15 Yan, Y., Wang, T., Zhang, R., Liu, Y., Hu, W. & Sitti, M. Magnetically assisted soft milli-tools for occluded lumen morphology detection. Science Advances 9, eadi3979 (2023).

16 Hwang, J., Kim, B., Jin, C., Lee, G., Jeog, H., Lee, H., Noh, J., Lim, S. J., Kim, J. Y. & Choi, H. Shortwave infrared imaging of a quantum dot‐based magnetic guidewire toward non‐fluoroscopic peripheral vascular interventions. Small 21, 1613-6810 (2025).

17 Dreyfus, R., Boehler, Q., Lyttle, S., Lussi, P. G. J., Chautems, C., Berberat, S. G. J., Seibold, D., Reinehr, N. O. M., Remonda, M. W. L. & Nelson, B. J. Dexterous helical magnetic robot for improved endovascular access. Science Robotics 9, eadh0298 (2024).

18 Kim, Y., Parada, G. A., Liu, S. & Zhao, X. Ferromagnetic soft continuum robots. Science Robotics 4, eaax7329 (2019). .

19 Mao, L., Yang, P., Tian, C., Shen, X., Wang, F., Zhang, H., Meng, X. & Xie, H. Magnetic steering continuum robot for transluminal procedures with programmable shape and functionalities. Nature Communications 15, 3759 (2024).

20 Pancaldi, L., Dirix, P., Fanelli, A., Lima, A. M., Stergiopulos, N., Mosimann, P. J., Ghezzi, D. & Sakar, M. S. Flow driven robotic navigation of microengineered endovascular probes. Nature Communications 11, 6356 (2020).

21 Ge, T. J., Roquero, D. M., Holton, G. H., Mach, K. E., Prado, K., Lau, H., Jensen, K., Chang, T. C., Conti, S., Sheth, K., Wang, S. X. & Liao, J. C. A magnetic hydrogel for the efficient retrieval of kidney stone fragments during ureteroscopy. Nature Communications 14, 2041-1723 (2023).

22 Li, N., Lin, D., Wu, J., Gan, Q. & Jiao, N. Dexterity of concentric magnetic continuum robot with multiple stiffness. (International Conference on Intelligent Robotics and Applications, 329-338 2023).

23 Kim, Y., Genvriere, E., Harker, P., Choe, J., Balicki, M., Regenhardt, R. W., Vranic, J. E., Dmytriw, A. A., Patel, A. B. & Zhao, X. Telerobotic neurovascular interventions with magnetic manipulation. Science Robotics 7, eabg9907 (2022).

24 Tiryaki, M. E., Elmacıoğlu, Y. G. & Sitti, M. Magnetic guidewire steering at ultrahigh magnetic fields. Science Advances. 9, eadg6438 (2023).

25 Gharamaleki, N. L., Kim, D. i., Lee, G., Kim, J. Y. & Choi, H. Magnetic field control using an electromagnetic actuation system with combined air‐core and metal‐core coils. Advanced Intelligent Systems 7, 2640-4567 (2025).

26 Lu, K., Zhou, C., Li, Z., Liu, Y., Wang, F., Xuan, L. & Wang, X. Multi-level magnetic microrobot delivery strategy within a hierarchical vascularized organ-on-a-chip. Lab on a Chip 24, 446-459 (2024).

27 Diller, E. D., Giltinan, J., Lum, G. Z., Ye, Z. & Sitti, M. Six-degrees-of-freedom remote actuation of magnetic microrobots. (Robotics: Science and Systems 2014).

28. Li, R., Wang, Jun., Zhao, X., Liu, Z., Jia, P., Liu, Y., Lin, G., Xu, H., Xiong, J. Small-scale magnetic soft robotic catheter for in-situ biomechanical force sensing. Biosensors and Bioelectronics 270, 116977 (2025).

29. Zhang, M., Yang, L., Yang, H., Su, L., Xue, J., Wang, Q., Hao, B., Jiang, Y., Chan, K., Sung, J., Ko, H., Liu, X., Wang, L., Ming, B., Leung, T., Zhang, L. A magnetically actuated microcatheter with soft rotatable tip for enhanced endovascular access and treatment efficiency. Science Advances 11, 25 (2025).

30. Erin, O., Chen, X., Bell, A., Raval, S., Schwehr, T., Liu, X., Addepalli, P., Mair, L., Weinberg, I., Mercado, Y., Krieger, A. Strong magnetic actuation system with enhanced field articulation through stacks of individually addressed coils. Scientific Reports 14, 23123 (2024).

31. Peng, Q., Wang, S., Han, J., Huang, C., Yu, H., Li, D., Qiu, M., Cheng, S., Wu, C., Cai, M., Fu, S., Chen, B., Wu, X., Du, S., Xu, T. Thermal and Magnetic Dual-Responsive Catheter-Assisted Shape Memory Microrobots for Multistage Vascular Embolization. Research 7, (2024).

32. Ligtenberg, L., Rabou, N., Goulas, C., Duinmeijer, W., Jalfwerk, F., Arens, J., Lomme, R., Magdanz, V., Klingner, A., Rot, E., Nijland, C., Wasserberg, D., Liefers, H., Jonkheijm, P., Arce, A., Warle, M., Khalil, I. Ex vivo validation of magnetically actuated intravascular untethered robots in a clinical setting. Communications Engineering 68 (2024).

33. Zielasek, C., Lussi, J., Viviani, S., Makaloski, V., Kotelis, D., Jungi, S. In vitro renal artery stenting using a steerable guide wire navigated by a mobile electromagnetic field. Journal of Vascular Surgery Cases, Innovations and Techniques 101842 (2025).

34. Wang, B., Wang, Q., Chan, K., Ning, Z., Wang, Q., Ji, F., Yang, H., Jiang, S., Zhang, Z., Ip, B., Ko, H., Chung, J., Qiu, M., Han, J., Chiu, P., Sung, J., Du, S., Leung, T. tPA-anchored nanorobots for in vivo arterial recanalization at submillimeter-scale segments. Science Advances 10, 5 (2024).

35. Pancaldi, L., Gadiri, E., Raub, J., Mosimann, P., Sakar, M. Flow-driven magnetic microcathe for superselective arterial embolization. Science Robotics 10, 107 (2025).

36. Yang, S., Shen, J., He, J., Xia, Z., Zhang, Y., Wang, B. Magnetic slippery microcatheter with artificial cilia for low-friction interventions. Science Advances 11, 42 (2025).
